# Self-grown mycelium in confined geometries as nanofluidic devices

**DOI:** 10.1038/s41467-026-72999-0

**Published:** 2026-05-15

**Authors:** Qilong Cheng, Zhenyuan Niu, Bryce Waller, Bingyu Xia, Pengfei Deng, Yanpei Tian, David M. Warsinger, Zuzanna S. Siwy, Xianming Dai, Gregory Bonito, Tian Li

**Affiliations:** 1 School of Mechanical Engineering, Purdue University, West Lafayette, IN USA; 2 Department of Microbiology, Genetics, & Immunology, Michigan State University, East Lansing, MI USA; 3 Department of Physics and Astronomy, University of California, Irvine, CA USA; 4 Department of Engineering Technology & Industrial Distribution, Texas A&M University, College Station, TX USA; 5 Department of Plant, Soil and Microbial Sciences, Michigan State University, East Lansing, MI USA

**Keywords:** Biomaterials, Mechanical engineering

## Abstract

Precise control of ion and molecular transport at the nanoscale underpins next-generation nanofluidic technologies. However, current approaches such as top-down fabrication and bottom-up assembly remain constrained by cost, scalability, or limited programmability. Fungal mycelium—the largest natural ion transport network in soil—offers a living bio-derived route to nanofluidics. Here, we harness mycelium’s self-growth and hyphal anastomosis to construct nanofluidic structures that autonomously conform to confined geometries. With interconnected fibrous networks, nanoscale porosity, and negatively charged surfaces (−2.8 to −4.1 mC m^−2^), multispecies mycelium generates in situ adaptive pathways through channels, gaps, and open volumes. Specifically, a mycelium-integrated microchannel achieves a pH-gating switch ratio of up to 3.0 and a 55-fold enrichment for dilute cation detection. These results establish the principle that nanofluidic functionality can be biologically grown rather than fabricated, introducing a scalable, sustainable, and geometrically adaptable platform. By bypassing lithography and energy-intensive processing, this bio-derived strategy may enable living and self-organizing ion transport networks with potential applications in sensing, ionic computing, and energy conversion.

## Abstract (web summary)

Nanofluidic technologies require precise control of ion and molecular transport at the nanoscale, yet existing fabrication methods face limitations in cost, scalability, and programmability. Authors demonstrate the use of self-grown fungal mycelium to create adaptable nanofluidic devices that exhibit significant ion transport capabilities.

## Introduction

Nanofluidics, which focuses on fluid transport in channels with characteristic dimensions typically below 100 nm, enables unprecedented control over ions and molecules at the nanoscale^1,2^. At these dimensions, Debye length—the range of electrostatic interactions—becomes comparable to the channel size, resulting in overlapping electric double layers (EDLs) and dominance of surface-governed transport over bulk diffusion^3^. Such geometric confinement gives rise to ion-regulation phenomena, including selectivity^4,5^, rectification^6,7^, and memory effects^8,9^, that are absent in macroscale or even microscale conduits. Consequently, nanofluidic systems hold great promise for applications such as biosensing^10,11^, filtration^4,12^, and energy harvesting^13,14^, where precise ion transport is essential. Positioned at the intersection of physics, chemistry, and biology, nanofluidics serves as a vital platform for advancing the understanding of nanoscale transport^15,16^ and enabling device designs beyond conventional microfluidics^17^.

Multiple material families (e.g., silicon^18,19^, alumina^20,21^, and polymers^8,22^) have been widely explored for nanofluidic devices and have become well-established platforms for investigating nanoscale transport. These systems are commonly fabricated using top-down techniques, such as electron-beam or photolithographic patterning followed by etching to define nanopores or nanochannels in dielectric substrates. While these approaches provide high structural precision and surface control, they typically require complex cleanroom processing, exhibit relatively low fabrication throughput, and are largely confined to rigid planar geometries, limiting scalability and geometric adaptability. In contrast, bottom-up strategies—including the assembly of low-dimensional materials (e.g., graphene slit channels^4,16,23^, carbon nanotube membranes^24,25^, and boron nitride nanochannels^26^) and biomolecular structures such as DNA nanopores^27,28^ and protein nanopores^10^—offer broader material diversity and intrinsic molecular-scale confinement. However, graphene and boron nitride slit-channel devices often rely on mechanically assembled heterostructures with limited geometric programmability, while carbon nanotube membranes and biomolecular nanopores are typically difficult to fabricate in high throughput or to integrate into interconnected nanofluidic transport networks over large or non-planar surfaces.

In many of these nanofluidic platforms, surface-charge-governed ion transport phenomena such as selectivity^4,5^, rectification^6,7^, and pH-dependent conductance^29,30^ have been widely investigated. These effects are commonly observed in charged nanopores^12,22,29^, nanochannels^31–33^, and nanoporous membranes^4,12,34^ used for ion-selective transport. However, these systems are typically based on predefined nanoscale architectures or synthetic nanoporous matrices fabricated through lithography or membrane assembly, which limits their ability to autonomously generate interconnected ion transport pathways within irregular three-dimensional confinements. Overcoming these constraints calls for alternative fabrication paradigms that simultaneously deliver nanofluidic functionality, scalability, and geometric adaptability.

Biomaterials offer a promising opportunity for constructing microfluidic and nanofluidic networks^35–37^ owing to their intrinsic hierarchical architectures and growth-enabled connectivity. A variety of bio-derived or bio-templated transport systems have been explored to guide molecular and ionic transport, including porous membranes fabricated from natural biomaterials^37,38^, transport pathways formed within bacterial biofilms^39,40^, and vascular transport architectures found in plant tissues such as leaf veins^41^. However, most of these approaches rely on static biological templates or processed biomaterials and therefore do not exploit the intrinsic growth capability of living systems to autonomously generate interconnected transport pathways. Here, we introduce fungal mycelium as a living, low-cost, and scalable bio-derived scaffold for constructing nanofluidic pathways that can adapt to confined geometries without lithographic definition of nanoscale features. This capability arises from its bottom-up self-growth, hyphal branching and anastomosis, hierarchical porosity, and negatively charged surfaces.

**Fig. 1:**
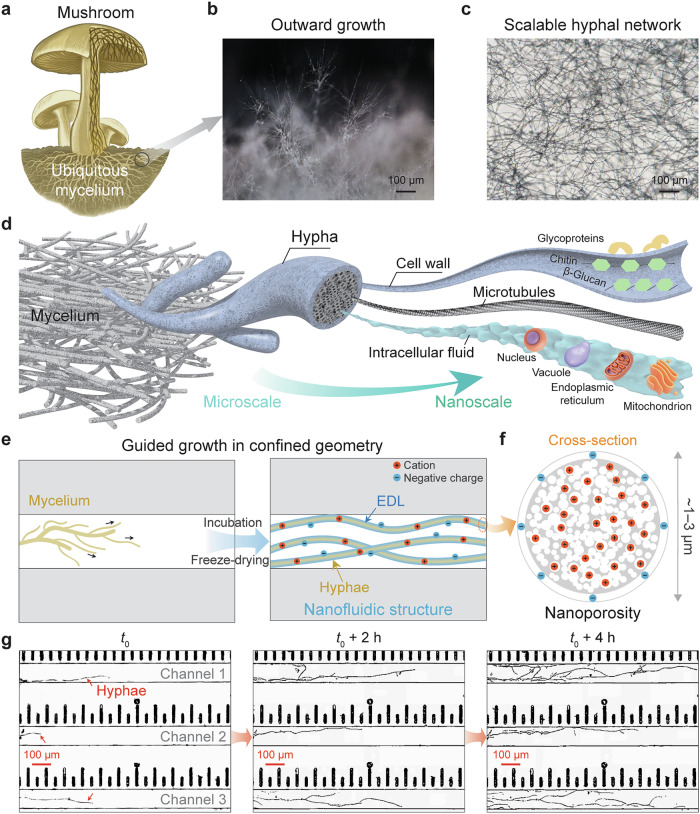
Ubiquitous and scalable mycelium and its guided growth in confined geometries. **a** Schematic of mycelium, the vegetative growth structure of mushrooms. **b** Photo of outward self-growing *Pleurotus ostreatus* mycelium, showing its branching capability. **c** Microscope image of *P. ostreatus* mycelium, featuring a scalable fibrous hyphal network. **d** Schematic showing the hierarchical structure of mycelium from microscale to nanoscale. The microscale hypha contains nanoscale microtubules and various organelles surrounded by intracellular fluid within its cell wall, which become nanoporous structures after freeze-drying. **e** Schematic of guided mycelium growth in a confined geometry, driven by growth resources such as nutrients and oxygen. The extended self-grown hyphae emerge as nanofluidic structures after freeze-drying, forming electric double layers (EDLs) that regulate cations, thus enabling nanofluidics. **f** Schematic of a cross-sectional hypha, showing its nanoporosity. The mycelial cell wall is inherently negatively charged due to the dissociation of functional groups, with nanopores within the hypha serving as pathways for cations. **g** Time-lapse microscope images of *Ganoderma sessile* hyphae growing within microchannels (Supplementary Fig. 3), following previously reported procedures^61,62^, showing their extensibility. The hyphae spread and traverse ~0.67 mm over 4 hours.

Mycelium, the vegetative growth structure of mushrooms (Fig. 1a), is ubiquitous in nature but often overlooked. It is composed of numerous fibrous hyphae, which form a network of thread-like filaments that serve as the main vegetative body of a fungus adapted for acquiring nutrition. Mycelial hyphae possess natural branching (Fig. 1b) and fusion capabilities, known as anastomosis (Supplementary Fig. 2), resulting in a scalable interconnected fibrous network (Fig. 1c). The mycelium network, when supported by appropriate nutrient supplies, can extend remarkably such as *Armillaria ostoyae* that spans 3810 m in Oregon, USA^42^, demonstrating its exceptional scalability. Moreover, mycelium growth is associated with minimal energy consumption^43^, requiring no external energy-intensive processing, which ensures the cost-effectiveness and sustainability of the proposed strategy.

Mycelium inherently exhibits a hierarchical porous structure from microscale to nanoscale, serving as a promising nanofluidic scaffold. As a fibrous network, mycelium consists of countless hyphae that are several micrometers in diameter (Fig. 1d left). Looking closer at a single hypha, it is surrounded by a shell-like cell wall composed mainly of chitin, β-glucan, and glycoproteins^44^, which maintains its physical structure (Fig. 1d right). Within the hypha are one or more mycelial cells with internal microtubules (~10–20 nm)^45^ and various organelles across multiple scales (e.g., nuclei, vacuoles, endoplasmic reticulum, and mitochondria) wrapped in intracellular fluid. The amount and distribution of these organelles vary across different species and even among the cells of the same hyphal strand. After freeze-drying, these solid structures, along with substances in the intracellular fluid, can be retained^46^ and form a nanoporous structure, making mycelium an attractive candidate for nanofluidics.

Leveraging its extensibility and hyphal anastomosis, mycelium can be guided to fill customized confined spaces and grow into desired device architectures (Fig. 1e). The extended hyphae naturally exhibit nanoscale porosity and negatively charged surfaces after freeze-drying, forming EDLs within the hyphae for ion transport regulation (Fig. 1e, f). Tracking the *Ganoderma sessile* hyphae growth within microchannels validates this mycelium-enabled strategy in confines spaces (Fig. 1g and Supplementary Movie 1). Furthermore, mycelium can be inoculated into diverse confined geometries, including channels, gaps, and open volumes, where it grows in situ to form adaptive pathways with pH-gating and rectification properties, showcasing that nanofluidic functionality can be biologically grown rather than fabricated. We also visualize the induced nanofluidic properties via ion staining and demonstrate a highly sensitive method for detecting low-concentration cations. This bottom-up architectural approach enables the formation of nanofluidic networks that autonomously adapt to their environment without requiring external patterning or high-energy processing, offering a fundamentally new route to scalable, sustainable, and geometrically adaptable nanofluidic systems.

## Results

### Multispecies mycelium: a universal nanofluidic scaffold

**Fig. 2:**
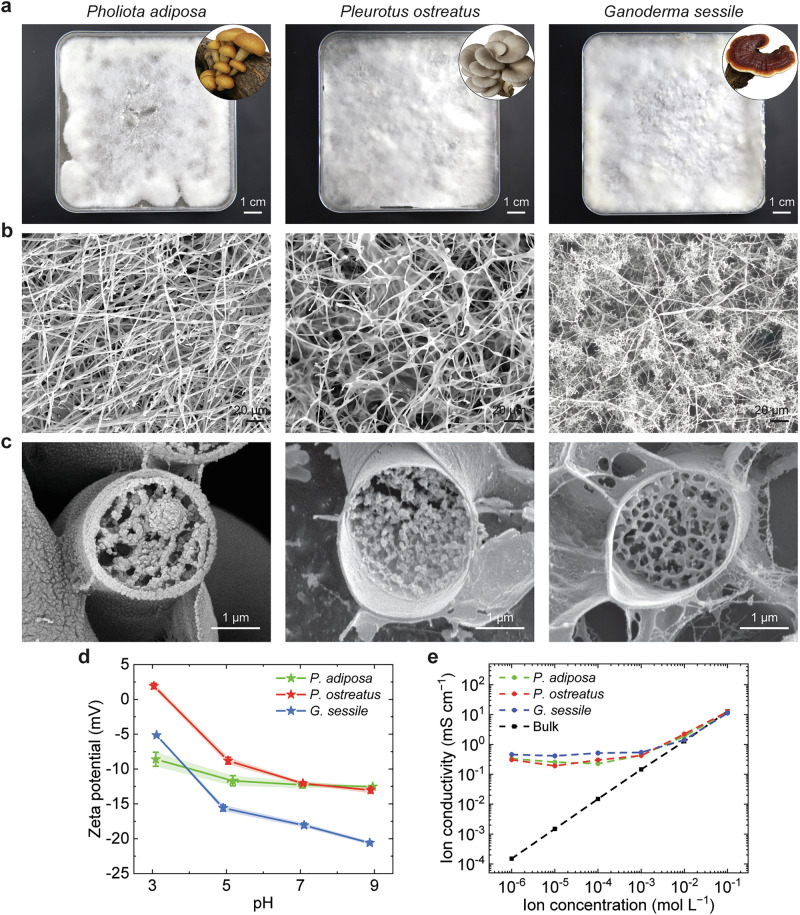
Universal micro-nanoporous structure and nanofluidic properties of multispecies mycelium. Photos (**a**) and SEM images (**b**) of three mycelium species—*Pholiota adiposa*, *Pleurotus ostreatus*, and *Ganoderma sessile*—showing a similar white appearance but distinct morphologies in their microscale fibrous networks. **c** CryoSEM images of a single hypha from the three species reveal a general nanoporous structure within the cell wall, featuring ion pathways for nanofluidic transport. **d** Zeta potential measurements of the three mycelium membranes, demonstrating their universally negative surface charge. **e** Ion conductivity measurements (Supplementary Fig. 14) of the three mycelium membranes show substantially higher conductivity than bulk values at KCl concentrations below 1 × 10^−3^ mol L^−1^, illustrating their universal ion regulation capability. Error bars represent the standard deviation of five samples from independently grown patches.

Natural mycelium grown on solid media appears as white, fluffy, and flocculent structures across multiple species, including *Pholiota adiposa*, *Pleurotus ostreatus*, and *Ganoderma sessile* (Fig. 2a). Although they display a similar white fibrous appearance in optical images (Supplementary Fig. 4), their scanning electron microscopy (SEM) images reveal distinct hyphal morphologies, where *P. adiposa* develops long and straight fibers, *P. ostreatus* forms slightly curved fibers with sheet-like structures, and *G. sessile* displays a mixture of long straight fibers and short coral-like fibers that are only hundreds of nanometers wide (Fig. 2b and zoom-in images in Supplementary Fig. 5). With longer incubation, however, the hyphae of all three species converge towards a similar morphology at the mature stage after one-month growth (Supplementary Fig. 6), illustrating the structural similarity across different mycelium species.

Scaling down to a single hypha reveals a nanoporous structure, as illustrated by scanning electron cryomicroscopy (CryoSEM) images (Fig. 2c). The internal pores at the cross-sections span a wide size range, from tens to hundreds of nanometers (Supplementary Fig. 7), and are enclosed by a sheet-like cell wall that is thinner than 100 nm. Previous studies have reported that the hyphal cell wall itself contains intrinsic nanopores of ~2–3 nm^47^. Together, the larger internal pores and the intrinsic cell-wall nanopores enable the hyphae to function as an interconnected nanoporous network for ion transport.

Mycelium membranes from three species consistently show negative zeta potentials and exhibit the same trend that their zeta potentials decrease with increasing pH (Fig. 2d). Deprotonated acidic groups (e.g., carboxyl and phosphate) in cell wall glycoproteins and polysaccharides^48^ dissociate under physiological pH, yielding a net negative zeta potential. Under neutral conditions of pH = 7, they show zeta potentials ranging from −12 to −18 mV, corresponding to surface charges of −2.8 to −4.1 mC m^−2^ (Supplementary Note 1). KCl electrolyte solutions at different concentrations were used for conductivity measurements throughout this study. The negative surface charge, synergistic with hyphal ion pathways, contributes to surface-mediated K^+^ transport in the mycelial scaffold and confers a common multi-species ion regulation capability (Fig. 2e and Supplementary Figs. 8–10), a typical nanofluidic characteristic where high ion conductivity is observed even at low ion concentrations (<1 × 10^−3^ mol L^−1^).

The ion transport in the mycelial network is primarily associated with nanoconfined channels within the hyphae rather than microscale void spaces between hyphae. The spaces between hyphae observed in Fig. 2b are typically on the order of tens of micrometers, which are substantially larger than the Debye length in dilute electrolytes (typically ~10–100 nm). Such large channels do not support electric double layer overlap and therefore cannot sustain nanofluidic transport. This interpretation is further supported by compression tests, where compressed samples with reduced void volume between hyphae exhibit ion conductivities comparable to those of uncompressed samples (Supplementary Fig. 11). The nanoporous structure within the hyphae is also expected to carry negative charge similar to the hyphal surface, likely arising from cell membrane disruption and the dissociation of functional groups, thereby allowing cation migration within the hyphal interior. In addition, dye-tracing experiments reveal that the mycelial hyphae form an interconnected network supporting continuous ion transport pathways (Supplementary Fig. 12), enabled by hyphal fusion and extracellular matrix components^45,49^. Comparisons between live and freeze-dried mycelium further highlight the role of nanoporosity generated during freeze-drying in enabling nanofluidic ion transport (Supplementary Fig. 13).

This nanofluidic conductivity trend follows the equation for bulk- and surface-governed ion transport:

1 $$\kappa=q({\mu }_{+}+{\mu }_{-})C{N}_{{{{\rm{A}}}}}+2{\sigma }_{{{{\rm{s}}}}}{\mu }_{+}/d$$

where *q* is the ion charge, *μ*_+_ and *μ*_−_ are mobilities of cations and anions, *C* is the ion concentration, *N*_A_ is Avogadro’s number, *σ*_s_ is the surface charge, and *d* is the equivalent nanochannel diameter. At low ion concentrations, the bulk-governed term $q({\mu }_{+}+{\mu }_{-})C{N}_{{{{\rm{A}}}}}$ becomes negligible, while the surface-governed term $2{\sigma }_{{{{\rm{s}}}}}{\mu }_{+}/d$ establishes a plateau, thereby enhancing ion transport beyond bulk behavior and enabling effective ion regulation. It should be noted that this expression was originally derived for an idealized single cylindrical nanochannel. In contrast, mycelium forms a nanoporous scaffold in which ion transport occurs through a distributed hyphal network with multiple parallel pathways rather than a single long nanochannel, resulting in collective transport behavior within this interconnected nanoporous architecture. The conductivity plateaus observed across mycelium species indicate that ion regulation is an emergent and universal property of fungal hyphal networks rather than species-specific, highlighting the potential of mycelial scaffolds as bio-derived platforms for iontronic applications.

### Nanofluidic pathways via guided mycelium growth

**Fig. 3:**
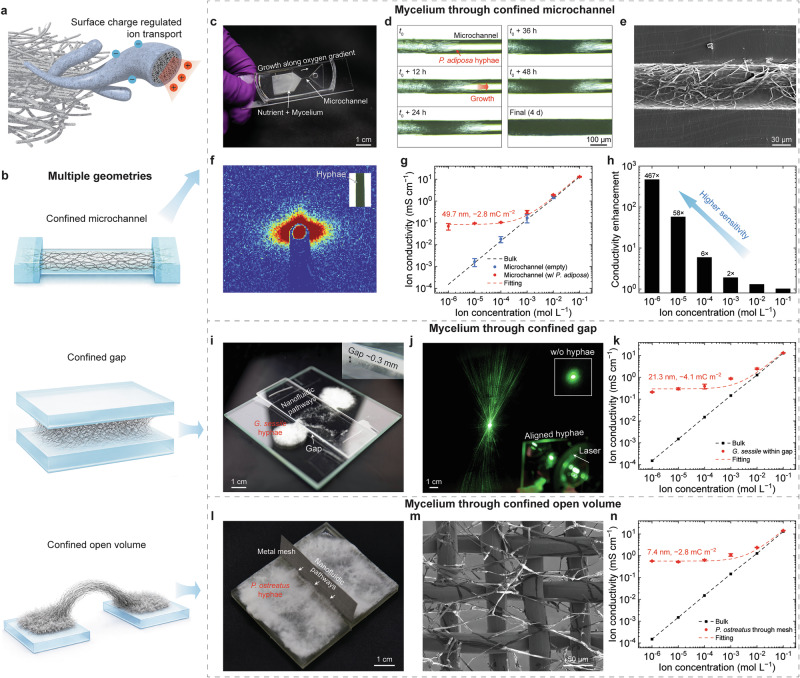
Self-grown mycelium-enabled nanofluidics. **a** Schematic of surface charge regulated ion transport in mycelium. **b** Demonstration of guided mycelium growth forming nanofluidic pathways across multiple device geometries. **c**–**h** *P. adiposa* mycelium through a confined microchannel. **c** Photo of the mycelium-integrated microfluidic device, where one side is filled with nutrients and mycelium spores, allowing mycelium to grow through the interconnecting microchannel following the oxygen gradient. **d** Time-lapse microscope images of mycelium growth through the microchannel. **e** SEM image of the hyphal network in the microchannel. **f** SAXS image of hyphae in the microchannel, showing their alignment. **g** Ion conductivity of the microchannel when empty versus with *P. adiposa* hyphae, showing that the integration of mycelium introduces nanofluidic ion regulation performance. **h** Conductivity enhancement by mycelium. **i**–**k** *G. sessile* mycelium through a confined gap. **i** Photo of mycelium within a gap of ~0.3 mm, establishing nanofluidic pathways between the two sides. **j** Laser diffraction pattern of hyphae on glass, oriented from left to right, showing their alignment. **k** Ion conductivity of the mycelium within the gap, with a conductivity plateau ~0.3 mS cm^−1^. **l**–**n** *P. ostreatus* mycelium through a confined open volume. **l** Photo of mycelium forming nanofluidic pathways through a metal mesh. **m** SEM image of the hyphal network through the mesh. **n** Ion conductivity of the mycelium through the mesh, with a conductivity plateau ~0.6 mS cm^−1^. All error bars represent the standard deviation of measurements from at least three devices fabricated from independently grown patches.

Leveraging its inherent negative surface charge along with natural nanoporosity, the self-growing property of mycelium can be harnessed to spontaneously construct nanofluidic pathways through various complex geometries, including channels, gaps, and open volumes (Fig. 3a, b). Microfluidic devices have been extensively investigated for biological diagnosis and beyond^50,51^, but they lack enhanced capillary effects at the nanoscale and their electroneutrality prevents surface-mediated transport. Here, we demonstrate that, through mycelium incubation in a microchannel, the microfluidic device can be transformed into a nanofluidic one with enhanced conductivity (Fig. 3c–h). This approach differs fundamentally from previously reported nanoporous matrices formed within microscale channels^52–54^, as it relies on a self-grown biological scaffold in which ion transport is governed by charged hyphae rather than by a static synthetic monolith. Using a simple thin copper wire, a microchannel ~88 μm in diameter was constructed (Supplementary Fig. 15), and mycelium grew along the oxygen gradient through the microchannel (Fig. 3c), with time-lapse microscope images tracking the filling process of mycelium inside the microchannel (Fig. 3d).

Mycelium is known to have aerial and submerged forms^55^, both of which are achievable in the microchannel (Supplementary Movies 2–4). It takes ~30 h for aerial mycelium to fill a cross-sectional plane in the microchannel (Supplementary Fig. 16) and several days to traverse the entire microchannel, while submerged mycelium grows ~9 times faster (Supplementary Fig. 17), because the nutrients are supplied directly from the surrounding liquid medium rather than being transported through the hyphal network as in aerial mycelium. Comparable network formation is observed across different mycelium species (*G. sessile* and *P. adiposa*), microchannel geometries (rectangular and cylindrical), and growth environments (aerial, submerged, and mixed), as shown in Supplementary Movies 1–4, indicating reproducible formation of mycelial networks within confined microchannels across a range of growth conditions. SEM images further confirm the interwoven hyphal network in the microchannel (Fig. 3e and Supplementary Fig. 18), which enables nanofluidic ion transport. The hyphae in the channel show partial alignment, as evidenced by small-angle X-ray scattering (SAXS, Fig. 3f) and laser diffraction patterns (Supplementary Fig. 19), reflecting their unidirectional growth from the nutrient side toward the oxygen-rich side.

Thanks to the nanofluidic properties of mycelium, the integrated microchannel exhibits previously absent ion regulation capability (Fig. 3g) under the same KCl electrolyte conditions. The I–V curves are used to derive the conductance (Supplementary Fig. 20), which is further converted to ionic conductivity using the length and diameter of the microchannel. While the empty channel shows the same ion conductivity as the bulk solution, the mycelium-integrated channel displays a conductivity plateau of ~0.1 mS cm^−1^ at KCl concentrations below 1 × 10^−3^ mol L^−1^—a hallmark of nanofluidics. While access resistance may contribute to the overall device resistance, its magnitude is expected to be much smaller than the resistance of the extended mycelial network spanning the millimeter-scale microchannel. This interpretation is further supported by the observation that the device performance is consistent with that of bulk mycelium materials (Fig. 2e).

The conductivity may also depend on the filling density of the hyphal network within the microchannel, which varies with mycelium species, incubation time, and nutrient supply. A lower filling density leads to fewer available ion transport pathways and consequently lower effective conductivity of the microchannel. Precise control of the filling density for biologically grown networks remains challenging and requires further investigation. Moreover, the conductivity does not depend on the growth direction as indicated by the symmetric I–V curves (Supplementary Fig. 20), and is also not expected to depend strongly on the channel geometry. This is because the mycelium serves as the primary ion conduction medium and forms a fibrous network composed of numerous interconnected hyphae, which reduces structural anisotropy and minimizes directional asymmetry in ion transport. When the channel size is significantly reduced and the mycelial network contains only a few hyphae, however, the transport behavior may change, enabling future studies of single-hypha or few-hypha nanofluidic devices.

Using *P. adiposa* with a surface charge of −2.8 mC m^−2^, conductivity modeling indicates that the mycelium-integrated microchannel is equivalent to a 49.7 nm nanochannel in terms of ion transport (Supplementary Note 2). This effective channel size reflects the collective transport behavior of nanopores within the mycelial cell walls (~several nm) and the hyphal interior (~10–100 nm). Meanwhile, the nanofluidic mycelium enhances ion conductivity by up to 467-fold for this microchannel device (Fig. 3h), opening opportunities for sensitive ion detection, and undoubtedly, the strategy is readily applicable to other microfluidic devices.

Beyond channels, nanofluidic pathways can also be constructed within more complex geometries, such as gaps and open volumes, using species other than *P. adiposa* to illustrate the strong versatility of the self-grown mycelium. It is demonstrated that *G. sessile* mycelium can traverse through a gap of ~0.3 mm between glass pieces (Fig. 3i), interconnecting the two open sides. The extended hyphae through the gap show alignment within ~±25° as observed from the laser diffraction pattern (Fig. 3j). The ion conductance of the *G. sessile* network was derived from the I–V measurements (Supplementary Fig. 21) and then converted to ion conductivity using estimated geometric parameters (see Methods and Supplementary Note 3). Similar to the microchannel device with mycelium, the *G. sessile* network exhibits an ion conductivity plateau of ~0.3 mS cm^−1^ at low KCl concentrations, corresponding to an equivalent 21.3 nm nanochannel given the *G. sessile* surface charge of −4.1 mC m^−2^ (Fig. 3k). The sensitivity of the inferred nanochannel size to the assumed geometric parameters is further discussed in Supplementary Note 4.

Furthermore, *P. ostreatus* mycelium is utilized to establish an aerial suspended hyphal network between two nutrient reservoirs through a metal mesh with holes ~37 μm (Fig. 3l), serving as volumetric nanofluidic pathways. The hyphae crawl on the metal wires and penetrate through the mesh (Fig. 3m), demonstrating the mycelial capability to attach and climb. The nonlinear conductance derived from the I–V measurements (Supplementary Fig. 22), together with the ion conductivity of the *P. ostreatus* hyphal network (Fig. 3n), further demonstrate the universal nanofluidic ion regulation capability of mycelium. Conductivity modeling indicates an equivalent nanochannel size of 7.4 nm, consistent with the observation that *P. ostreatus* contains a higher proportion of smaller nanopores (Supplementary Fig. 7). The estimation of the geometric parameters of the nanofluidic pathways and the corresponding sensitivity analysis are provided in Supplementary Notes 3–4.

Moreover, the *P. ostreatus* mycelium can penetrate an enclosed cage made of metal mesh (Supplementary Fig. 23), further validating its breakthrough capability. Beyond the aforementioned geometries, mycelium also possesses the capability to self-assemble into membranes and foams (Supplementary Fig. 24), enabling greater control over the dimensionality of the resulting structures. Therefore, by leveraging the mycelial self-growing behaviors, mycelium-based nanofluidic devices have been developed with geometric adaptability and demonstrated nanofluidic performance comparable to nanochannels, thereby eliminating the conventional need for energy-intensive processing.

### Mycelium-integrated device performance and visualization

**Fig. 4:**
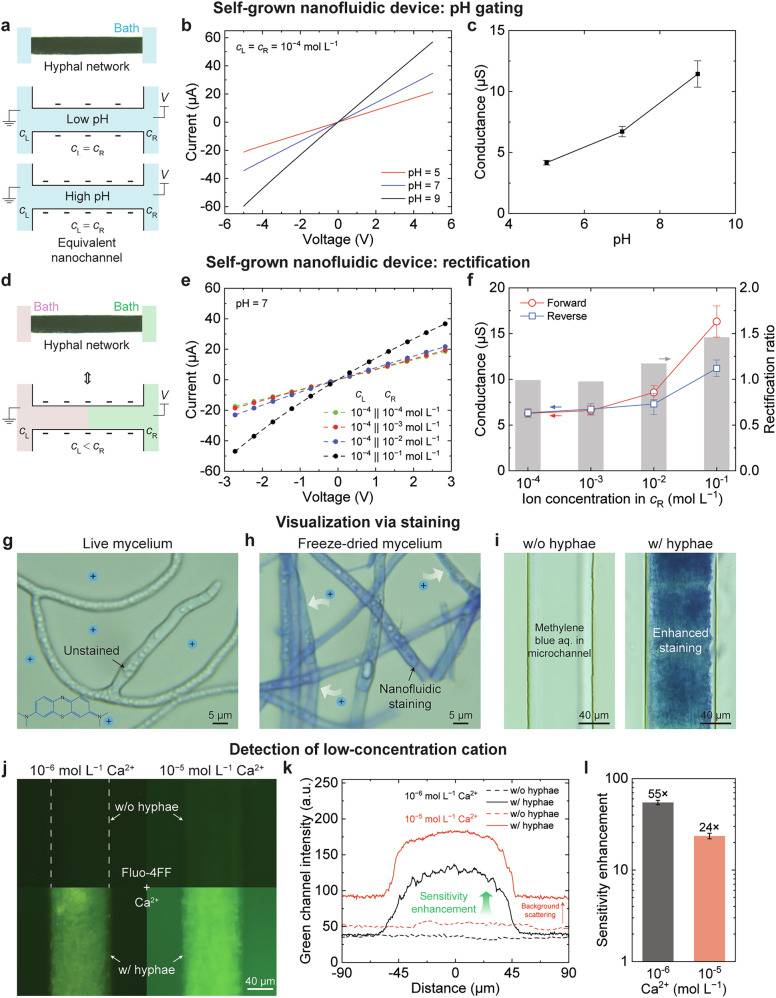
Performance and visualization of self-grown nanofluidic devices. **a**–**c** pH gating of a mycelium-integrated device. **a** Schematic illustrating that the negative surface charge can be tuned via pH. **b** Current–voltage response under varying pH. **c** The resultant conductance modulated by pH. **d**–**f** Rectification via asymmetric bath concentrations. **d** Schematic, **e** current–voltage response, and **f** the resultant rectification ratio induced by the bath concentration gradient. Microscope images of live (**g**) and freeze-dried (**h**) *P. adiposa* mycelium stained with a cationic dye (1 × 10^−4^ mol L^−1^ methylene blue solution), where only the freeze-dried sample exhibits nanofluidic staining within the hyphae. **i** Mycelium-integrated microchannel device shows enhanced staining due to hyphal attraction of the cationic dye. **j**–**l** Nanofluidic mycelium for sensitive cation detection. **j** The microchannel device with hyphae produces much stronger fluorescence when detecting Ca^2+^ due to nanofluidic Ca^2+^ enrichment, using the calcium indicator Fluo-4FF (1 × 10^−6^ mol L^−1^) that emits green light. **k** Radial profile of green channel intensity for low-concentration Ca^2+^ detection (10^−6^ and 10^−5^ mol L^−1^), extracted from processed images, indicating that the integrated hyphae clearly enhance the green signal. **l** Sensitivity enhancement derived from the green channel intensity (Supplementary Note 5). Error bars represent the standard deviation of measurements from three devices fabricated from independently grown patches.

Mycelium’s self-growing and self-adaptable capability allows it to fill confined spaces, unlocking opportunities for nanofluidic devices. Utilizing the inoculated hyphal network, the mycelium device exhibits typical nanofluidic properties, including pH gating (Fig. 4a–c) and concentration-asymmetric rectification (Fig. 4d–f). The zeta potential and surface charge of mycelium are dependent on pH conditions (Fig. 2d). Therefore, tuning pH can regulate the surface charge of the mycelium-integrated device. A higher pH facilitates the dissociation of functional groups in mycelial glycoproteins and polysaccharides, leading to a higher negative surface charge (Fig. 4a) and regulating ion transport, as observed from the current–voltage response (Fig. 4b). The resultant conductance increases by 3.0-fold as pH varies from 5 to 9 (Fig. 4c), demonstrating pH-gating switch behavior.

Further, under asymmetric bath concentrations, the mycelium device can also act as a nanofluidic diode (Fig. 4d). The different bath concentrations create different chemical potentials at the two ends of the device, resulting in diode-like current behavior (Fig. 4e), which is absent in the control device without mycelium (Supplementary Fig. 25). When one side is fixed at 1 × 10^−4^ mol L^−1^ and the other is increased from 1 × 10^−4^ mol L^−1^ to 1 × 10^−1^ mol L^−1^, the current increases and displays asymmetric diode responses, with the negative current (forward) exceeding the positive current (reverse). The forward and reverse conductances exhibit a modest rectification ratio of 1.46 ± 0.06 (Fig. 4f and Supplementary Fig. 26), which is consistent with that observed in bulk mycelium (Supplementary Fig. 27). This rectification arises from the 1000-fold electrolyte concentration asymmetry across the device combined with surface-charge-governed ion transport within the mycelial scaffold. The I–V curves do not show nonlinearity associated with concentration polarization, likely because ion transport occurs through a distributed mycelial network rather than a single long nanochannel. The interconnected hyphal pathways provide multiple parallel ion transport routes with relatively large effective diameters, which reduces the formation of strong ion depletion zones and suppresses the development of concentration polarization, resulting in moderate rectification. The rectification also does not show a dependence on the growth direction (Supplementary Fig. 28), which is attributed to the fibrous network of numerous interconnected hyphae that provide multiple ion transport pathways.

In addition to the concentration asymmetry, the rectification ratio shows a slight increase with applied voltage and pH (Supplementary Figs. 29–30), consistent with enhanced asymmetric ion transport under a stronger electric field and increased surface charge within the mycelial scaffold. Although the rectification ratio is moderate, this bio-derived material platform may be particularly suited for applications that benefit from adaptive or bio-integrated ionic transport^11,56,57^, including ion gating, environmental sensing, and living-material-based ion regulation, where moderate rectification combined with structural adaptability can enable functional regulation of ion transport. The demonstrations of pH gating and rectification validate that nanofluidic functionality can be biologically grown rather than fabricated, leveraging the self-growing and self-adaptable nature of mycelium.

Besides electrical responses, the mycelial network can also be readily visualized using cationic dyes due to nanofluidic ion enrichment, which helps its application in multiple scenarios such as biosensing and diagnosis. Immersed in 1 × 10^−4^ mol L^−1^ methylene blue solution, a small molecular cationic dye, live mycelium with retained cellular structure is unstained (Fig. 4g), as the blue cation cannot penetrate the intact cell membrane and may also be reduced to its colorless form by cellular metabolism^58^. In contrast, freeze-dried mycelium shows a staining effect, which we call “nanofluidic staining” (Fig. 4h). A washout test, in which the blue staining was largely removed after rinsing, suggests that the dye accumulation arises from transient ion enrichment within confined pathways rather than irreversible surface adsorption (Supplementary Fig. 31). After freeze-drying, the mycelial scaffold retains negatively charged surfaces and a porous structure, which can promote enrichment of cationic dye molecules under nanoconfinement. The intrinsic porosity of the mycelial network further facilitates dye penetration along the filamentous hyphae, resulting in uniformly stained mycelium. The mycelium-integrated microchannel successfully inherits this property, which can be further used for detection. For example, while the microchannel cannot detect methylene blue as a conventional microfluidic device, the introduction of nanofluidic mycelium concentrates the methylene blue cations, resulting in an enhanced staining effect (Fig. 4i).

To demonstrate the potential of nanofluidic mycelium for low-concentration cation detection, we chose Ca^2+^ and a fluorescent calcium indicator (Fluo-4FF). Ca^2+^ detection is crucial in cellular signaling^59^ and cardiac physiology^60^. The microchannel without mycelium shows no or faint green fluorescence when exposed to low Ca^2+^ concentrations of 10^−6^ mol L^−1^ and 10^−5^ mol L^−1^ (Fig. 4j top). However, the hyphae in the microchannel greatly enhance the fluorescent effect due to their nanofluidic ion enrichment capability (Fig. 4j bottom), compared with the control condition containing Fluo-4FF but no Ca^2+^ (Supplementary Fig. 32), thus expanding the detection range of Fluo-4FF. Wider-field images show uniform fluorescence enhancement in regions containing the mycelial network (Supplementary Fig. 33), while higher-magnification images show that the dye signal is distributed across the hyphae width rather than being confined to the outer boundary (Supplementary Fig. 34), consistent with local cation enrichment within nanoconfined hyphal pathways. The nanofluidic behavior is further supported by pH-dependent measurements probing the surface charge effect, where a more negative surface charge leads to stronger cation attraction (Supplementary Fig. 35). The enhancement is unlikely to originate from ion adsorption, as the fluorescence diminishes after a washout step (Supplementary Fig. 36), indicating reversible ion accumulation.

Notably, the background light intensity in the region outside the microchannel may increase due to optical scattering from the strong fluorescence within the microchannel. To address this issue, background subtraction was performed to extract the true fluorescence signal (Supplementary Note 5). The acquired fluorescent images were processed to extract the green color channel that Fluo-4FF emits (see Methods), and the radial profiles clearly demonstrate that the presence of hyphae remarkably enhances the green channel intensity (Fig. 4k), with enhancements of 55 ± 3-fold and 24 ± 2-fold for the two low concentrations (Fig. 4l). Further analysis shows that the signal-to-noise ratio (SNR) increases from 2.3 to 99.7 and from 3.5 to 79.0 for the two dilute Ca^2+^ solutions, respectively, due to the nanofluidic hyphal network (Supplementary Table 1). Correspondingly, the effective limit of detection (LOD) is improved by approximately one order of magnitude (Supplementary Note 5). It is envisioned that mycelium can enable sensitive detection of low-concentration cations through its nanofluidic ion enrichment.

## Discussion

This work demonstrates self-grown fungal mycelium as a self-adaptable material platform for nanofluidics. Mycelium, with its intrinsic negatively charged surfaces and hierarchical porosity as nanoscale transport pathways, offers a biological bottom-up scaffold with high structural adaptability, enabling scalable fabrication of nanofluidic networks (up to 0.167 mm h^−1^) through bio-derived manufacturing. By simply inoculating mycelium into confined spaces such as conventional microchannels, the self-grown mycelium transforms passive conduits into an active ion-regulating medium with pH-gating, diode properties, and ion enrichment at low concentrations, thereby enhancing visualization and broadening the cation detection range. Beyond microchannels as promising ion cables, the self-adaptable mycelium also exhibits the versatility to form membranes and even scaffolds, constructing nanofluidic pathways for complex geometries. Unlike conventional nanofabrication, which requires energy-intensive lithography or etching, mycelium spontaneously generates stable functional nanofluidic architectures under ambient conditions, with pronounced scalability, cost-effectiveness, and sustainability.

Looking forward, the growth-driven nature of mycelium opens opportunities for programmable nanofluidic architectures guided by channel geometry, nutrient gradients, or environmental stimuli. By tailoring microchannel dimensions comparable to the hyphal diameter, it may become possible to guide the growth of individual hyphae through confined geometries (Supplementary Fig. 37), enabling single-hypha nanofluidic devices for studying ion transport within individual biological conduits. Furthermore, such ion transport networks could be designed using microfluidic platforms to guide mycelial growth, enabling pathways with tunable connectivity and hierarchical structure. These capabilities suggest a new paradigm in which nanofluidic systems are not only fabricated but biologically grown and spatially organized, allowing adaptive ion transport pathways to emerge within complex microfluidic or biological environments.

This work establishes mycelium as a sustainable strategy for realizing ion regulation systems and as a living and adaptable platform bridging biology and nanotechnology. Beyond advancing fundamental nanofluidics, this approach demonstrates that nanofluidic functionalities can be grown rather than fabricated, offering a facile and scalable scheme for bio-integrated technologies. The self-grown mycelium is expected to be invaluable for iontronic systems, providing combined electrochemical and optical readouts as well as the possibility to electrically address various circuit components, thereby unlocking new opportunities in biosensing, bioelectronics, and energy harvesting.

## Methods

### Materials

Malt extract agar, malt extract, yeast extract, potassium chloride, calcium chloride, methylene blue, hydrochloric acid, and potassium hydroxide were purchased from Sigma Aldrich. Mycelium liquid cultures of *Pholiota adiposa*, *Pleurotus ostreatus*, and *Ganoderma sessile* were obtained from The Mushroom Lab @ Amazon.com. Fluo-4FF pentapotassium salt was purchased from ThermoFisher Scientific. Polydimethylsiloxane (PDMS, Dow Sylgard 184 silicone elastomer) was purchased from Ellsworth Adhesives. Glass slides and stainless steel mesh were purchased from Amazon.

### Mycelium nutrients

Both solid and liquid media were used for mycelium incubation. The solid medium was prepared by dissolving 5 g malt extract agar in 100 g deionized water at 80 °C, then cooling to form a hydrogel before use. The liquid medium was prepared by dissolving 4 g malt extract and 16 g yeast extract in 1 L deionized water, followed by sterilization in an autoclave.

### Mycelium membrane fabrication

Mycelium liquid culture was inoculated onto the solid malt extract agar medium and incubated for several days to form a white membrane. The mycelium grown on the solid medium was first frozen in a –20 °C freezer for 24 h and subsequently freeze-dried using a LABCONCO FreeZone 2.5 lyophilizer at –57 °C under a pressure below 20 Pa for over 24 h. The dried mycelium was then peeled off to obtain pure free-standing mycelium membranes, which were used for material characterization, zeta potential, and ion conductivity measurements.

### Mycelium-integrated nanofluidic device fabrication

The microchannel was fabricated by casting PDMS onto a copper wire (~88 μm in diameter and several centimeters in length). The two ends of the wire were wrapped with thermal adhesive tape (0.3 mm thick) to form small reservoirs, while the unwrapped section (~1 cm long) served as the microchannel template. After curing, the patterned PDMS was peeled off from the copper wire and bonded to a glass slide via oxygen plasma after trimming and hole drilling. Approximately 30 μL of solid or liquid nutrients were then added to one reservoir of the customized PDMS microfluidic device, and 10 μL of mycelium liquid culture was inoculated to enable growth through the microchannel for subsequent conductivity testing (Supplementary Fig. 15). The samples were cultivated for a sufficiently long incubation time (>10 days) to ensure a dense hyphal network within the microchannel, thereby maintaining measurement consistency.

As for the other two configurations, the gap was created by placing aluminum wires (0.3 mm in diameter) as spacers between a glass sheet (75 mm × 75 mm) and a glass slide (75 mm × 25 mm). Approximately 1 mL of solid nutrients were placed outside the gap, and liquid nutrients were placed within the gap to allow mycelium to grow from one side to the other, constructing nanofluidic pathways through the gap. The volumetric mycelium-integrated device was fabricated by placing solid nutrients into two discrete tanks (5 cm × 3 cm × 0.5 cm) separated by a stainless steel mesh (holes ~400 mesh) and inoculating 100 μL of mycelium liquid culture, enabling mycelium to grow through the mesh and form nanofluidic pathways between the two tanks. All devices were freeze-dried at –57 °C under a vacuum below 20 Pa for over 24 h to preserve nanoporosity within the mycelial hyphae for nanofluidic ion transport.

### Imaging and microscopy

Photos were acquired using a Canon EOS 5DSR camera. Microscope images were acquired using an OMAX microscope and a Nikon Eclipse Ti-E inverted microscope. SEM and CryoSEM images were acquired using a Thermo Scientific Apero 2. The pore size *D*_pore_ was estimated from SEM images by measuring the pore area *A*_pore_ and assuming an equivalent circular pore, where ${A}_{{{{\rm{pore}}}}}=\pi /4\times {D}_{{{{\rm{pore}}}}}^{2}$. The pore areas were extracted from the SEM images using ImageJ, and the pore size distribution was obtained using kernel density estimation of the calculated pore size data.

Fluorescent images were acquired using an Olympus BX-51 microscope equipped with a DP-71 camera and a U-MWB2 filter cube (blue excitation/green emission), with an exposure time of 5 s and ISO200 sensitivity. The mycelium-integrated microchannel was washed first to remove residual ions and then pre-soaked with Ca^2+^ solution at a specific concentration for one hour. Fluorescent images were acquired at the center of the microchannel immediately after introducing 1 × 10^−6^ mol L^−1^ Fluo-4FF solution without any applied voltage bias. Image analysis was performed using ImageJ. The image color was split into RGB channels and converted into grayscale images. The green channel was used for analysis because Fluo-4FF emits green fluorescence when bound to Ca^2+^. The region of interest (ROI) was defined as a line spanning the microchannel with a width equal to twice the microchannel diameter (Supplementary Fig. 38). Intensity profiles extracted from five such lines were averaged to obtain the green channel intensity without further normalization.

### Zeta potential and ion conductivity measurements

The zeta potentials of mycelium membranes immersed in 1 × 10^−2^ mol L^−1^ KCl solution were measured using a SurPASS 3 streaming potential analyzer. At the target pH values, streaming potentials were recorded and converted to zeta potentials via the Helmholtz-Smoluchowski equation under the predefined software settings. Three samples cultivated from independent patches were measured, and the error bars represent the standard deviation.

To measure ion conductivity, all samples were freeze-dried to preserve the nanostructured porosity of the hyphal network and eliminate metabolic activity associated with living cells. The samples were washed with deionized water to remove residual ions until the conductivity of the rinsed water was lower than 20 μS cm^–1^. This step minimizes the influence of residual nutrients, pre-existing ions, and materials released from the hyphae that could otherwise introduce uncontrolled variables and mask the intrinsic nanofluidic transport behavior (Supplementary Fig. 39). For microchannel samples, deionized water was introduced at both ends of the channel and the liquid was repeatedly removed using Kimwipes to facilitate flushing. The process was repeated until the conductivity of the deionized water at both ends of the microchannel was below 20 μS cm^–1^.

The electrical response was measured using linear sweep voltammetry (LSV) on a BioLogic VSP potentiostat. After a rest time of 5 s, the potential was scanned from –2.0 V to 2.0 V at a scan rate of 100 mV s^–1^. Ag/AgCl electrodes with a diameter of 4 mm were used (Supplementary Fig. 14). The samples were fully immersed and equilibrated in the KCl electrolyte for at least 24 h prior to measurement to ensure complete electrolyte exchange and avoid delayed ion response (Supplementary Fig. 40), such that the system reached steady-state ion transport before electrical characterization. The conductance *G*, the inverse of resistance, was determined from Ohm’s law, and the ion conductivity *κ* was calculated by

2 $$\kappa=\frac{GL}{A}$$

where *L* is the sample length and *A* is the sample cross-sectional area. The conductance was extracted from the linear slope of the I–V curves from –2 to 2 V. Error bars represent the standard deviation calculated from measurements of at least three devices from independently grown patches. The bulk ion conductivity in Fig. 3g was measured by testing the microchannel filled with KCl solution without any mycelial hyphae. The measured values agree well with the theoretical bulk ion conductivity of KCl solutions at different concentrations^37^ (dashed lines in Figs. 2e, 3g, k, and n).

For mycelium grown through confined gaps and open volumes, the geometric parameters (length and area) of the nanofluidic pathways are not well-defined or directly measurable, so a constant value of *L*/*A* is assumed such that the sample conductivity at 0.1 mol L^−1^ matches the corresponding bulk value (800 cm^–1^ for Fig. 3i and 900 cm^–1^ for Fig. 3l). The assumed values fall within the estimated *L*/*A* range: 326–1270 cm^–1^ for Fig. 3i and 446–1420 cm^–1^ for Fig. 3l (Supplementary Note 3). In addition, a sensitivity analysis of the *L*/*A* values on the inferred conductivity and equivalent nanochannel size is provided (Supplementary Note 4). As a result, qualitative conclusions based on conductance trends are robust and independent of geometric assumptions, whereas quantitative results (ion conductivity and inferred nanochannel size) are model-dependent and rely on the assumed *L*/*A* values. In contrast, the microchannel configuration does not require this *L*/*A* estimation because the channel length and cross-sectional area can be directly measured.

In the pH-gating tests, the pH of the bath KCl solutions was adjusted by adding 0.1 mol L^−1^ HCl or KOH solution. In the rectification tests, KCl solutions of different concentrations were added to the two ends of the microchannel device. The I–V curves were measured from –3 to 3 V with a voltage step of 0.5 V. The forward and reverse conductances were extracted as the linear slopes of the I–V curves under negative and positive bias, respectively, and the rectification ratio was defined as the ratio between these two conductances. Each I–V measurement was repeated three times for each device, and the reported values correspond to the averaged results. Error bars represent the standard deviation obtained from measurements on three independent devices fabricated from separately grown batches.

## Supplementary information

Supplementary Information

DescriptionofAdditionalSupplementaryFiles

Supplementary Movie 1. Submerged G. sessile in microchannel.mp4

Supplementary Movie 2. Aerial P. adiposa in microchannel.mp4

Supplementary Movie 3. Submerged P. adiposa in microchannel.mp4

Supplementary Movie 4. Aerial and submerged P. adiposa in microchannel.mp4

Transparent Peer Review file

## Source data

Source data

## Data Availability

The data that support the findings of this study are available from the corresponding author upon request. Source data are provided. Source data are provided with this paper.
