# Supplementary material for: Self-grown mycelium in confined geometries as nanofluidic devices
Source: Nat Commun. 2026 May 15;17:6461. doi: 10.1038/s41467-026-72999-0 (PMC13376398; doi:10.1038/s41467-026-72999-0)
Supplement: Supplementary file 1 — Supplementary Information [file 41467_2026_72999_MOESM1_ESM.pdf]

Supplementary Information

Self-Grown Mycelium in Confined Geometries as Nanofluidic Devices

Qilong Cheng[1†], Zhenyuan Niu[1†], Bryce Waller[2], Bingyu Xia[1], Pengfei Deng[1], Yanpei Tian[1], David Warsinger[1], Zuzanna Siwy[3], Xianming Dai[4], Gregory Bonito[2,5], Tian Li[1*]

1 School of Mechanical Engineering, Purdue University, West Lafayette, IN 47907, USA
2 Department of Microbiology, Genetics, & Immunology, Michigan State University, East Lansing, MI 48824, USA
3 Department of Physics and Astronomy, University of California, Irvine, Irvine, CA 92697, USA
4 Department of Engineering Technology & Industrial Distribution, Texas A&M University, College Station, TX 77843, USA
5 Department of Plant, Soil and Microbial Sciences, Michigan State University, East Lansing, MI 48824, USA
[*]Correspondence: tianli@purdue.edu
[†]Equal contribution: Qilong Cheng, Zhenyuan Niu

Table of Contents

Supplementary Notes: Supplementary Notes 1–5

Supplementary Figures: Supplementary Figures 1–40

References in Supplementary Information

Supplementary Notes

Supplementary Note 1. Derivation of surface charge from zeta potential

The zeta potentials ζ of three mycelium membranes (*P. adiposa*, *P. ostreatus*, and *G. sessile*) were measured to be from −12 to −18 mV under neutral conditions (pH = 7) at a KCl concentration C of 1×10^{-2} mol L^{-1}, which corresponds to surface charges σ_s of −2.8 to −4.1 mC m^{-2} according to the relation[1]:

$$\sigma_s = \frac{\varepsilon\varepsilon_0\zeta}{\sqrt{\varepsilon_0 k_B T/(Cq^2)}} \tag{S1}$$

where ε is the dielectric constant, ε_0 is the free space permittivity, k_B is the Boltzmann constant, T is the temperature, and q is the charge. Notably, the denominator is also known as the Debye length.

Supplementary Note 2. Conductivity modelling of bulk- and surface-governed ion transport

The ion conductivity κ within a nanochannel contains volumetric diffusion and EDL-governed surface ion transport, which can be described as[2]:

$$\kappa = q(\mu_+ + \mu_-)CN_\mathrm{A} + 2\sigma_\mathrm{s}\mu_+ / d \tag{S2}$$

where q is the ion charge, μ_+ and μ_- are mobilities of cations and anions, C is the ion concentration, N_A is Avogadro's number, σ_s is the surface charge, and d is the equivalent nanochannel diameter. The first term refers to bulk ion diffusion, while the second term refers to EDL-governed surface ion transport. The ion mobilities are determined by the Nernst-Einstein relation $\mu = {qD}/{k_\mathrm{B}T}$, where D is the diffusion coefficient of ions[3].

At low ion concentrations, the first term vanishes, resulting in a nearly constant conductivity plateau corresponding to the second term, which can be utilized to model the equivalent nanochannel size. At high ion concentrations, the bulk ion diffusion gradually takes over, producing a linear relationship between conductivity and concentration. The determination of the equivalent nanochannel size is achieved by minimizing the sum of relative errors of conductivities for low concentrations (10^{-6}, 10^{-5}, 10^{-4} mol L^{-1}).

Supplementary Note 3. Estimate for L/A values

In Figure 3i and Figure 3l, mycelium grows through confined gaps and open volumes, where the geometric parameters (length L and area A) of the nanofluidic pathways are not well-defined or directly measurable. This makes it difficult to derive exact ion conductivities. Therefore, an estimate of L/A is provided based on geometric considerations and literature-reported properties of mycelium networks.

For the gap configuration (Figure 3i), the length of the nanofluidic pathway corresponds to the width of the glass slide (L = 2.54 cm). The total cross-sectional area within the 0.3 mm gap is A_{total} = 0.3 mm × 1.3 cm = 0.039 cm^2. However, mycelium forms a sparse filamentous network and does not occupy the entire cross-section. Previous studies[4, 5] report bulk densities of mycelium networks on the order of 50–200 kg m^{-3}, while individual hyphae—containing 80–90% water—have densities close to that of water (1000–1100 kg m^{-3}). This suggests that the mycelium network has a filling ratio f around 5–20%. Based on this estimate, the effective conductive cross-sectional area is $A_{\mathrm{eff}} = A_{\mathrm{total}} \times f$ = 0.002–0.0078 cm^2. Consequently, the corresponding L/A range is 326–1270 cm^{-1}. The nominal value used in the conductivity calculation, 800 cm^{-1}, falls within this physically reasonable range.

For the open volume configuration (Figure 3l), the length of the nanofluidic pathway corresponds to the tilted distance between the two agar tanks. The horizontal separation between the two tanks is 0.5 cm, and the hyphae grow at a titled angle ~30°–45°, giving a conductive pathway length L = 0.58–0.71 cm. To estimate the effective cross-sectional area, we count the number of mesh grids N_{grid} through which the hyphae pass: N_{grid} ~ 1500–1800. Each grid contains hyphae number N_{hyphae} = ~5–10, with a hyphae diameter ~3 μm as estimated from the SEM image (Figure 3m). Based on this estimate, the effective conductive cross-sectional area is $A_{\mathrm{eff}} = N_{\mathrm{grid}} \times N_{\mathrm{hyphae}} \times \pi/4 \times (3\ \mu\mathrm{m})^2$ = 0.0005–0.0013 cm^2. Consequently, the corresponding L/A range is 446–1420 cm^{-1}. The nominal value used in the conductivity calculation, 900 cm^{-1}, falls within this physically reasonable range.

Supplementary Note 4. Sensitivity based on *L*/*A* values

The ion conductivity κ was calculated from $\kappa = \frac{GL}{A}$. Therefore, for a given measured conductance *G*, the inferred conductivity κ (including the plateau magnitude) increases linearly with the assumed *L*/*A* value. Meanwhile, the extracted equivalent nanochannel size based on the relation $\kappa = q(\mu_{+} + \mu_{-})CN_{\mathrm{A}} + 2\sigma_{\mathrm{s}}\mu_{+} / d$ changes accordingly.

To evaluate this sensitivity, we provide results calculated using multiple *L*/*A* values within the estimated bounds (Supplementary Figure 1). For the gap configuration, data are provided for *L*/*A* = 400 cm^{-1}, 800 cm^{-1} (nominal), and 1200 cm^{-1} within the estimated range of 326–1270 cm^{-1}. For the open volume configuration, data are provided for *L*/*A* = 500 cm^{-1}, 900 cm^{-1} (nominal), and 1400 cm^{-1} within the estimated range of 446–1420 cm^{-1}. The resulting equivalent nanochannel sizes remain within the same order of magnitude across the estimated geometric range.

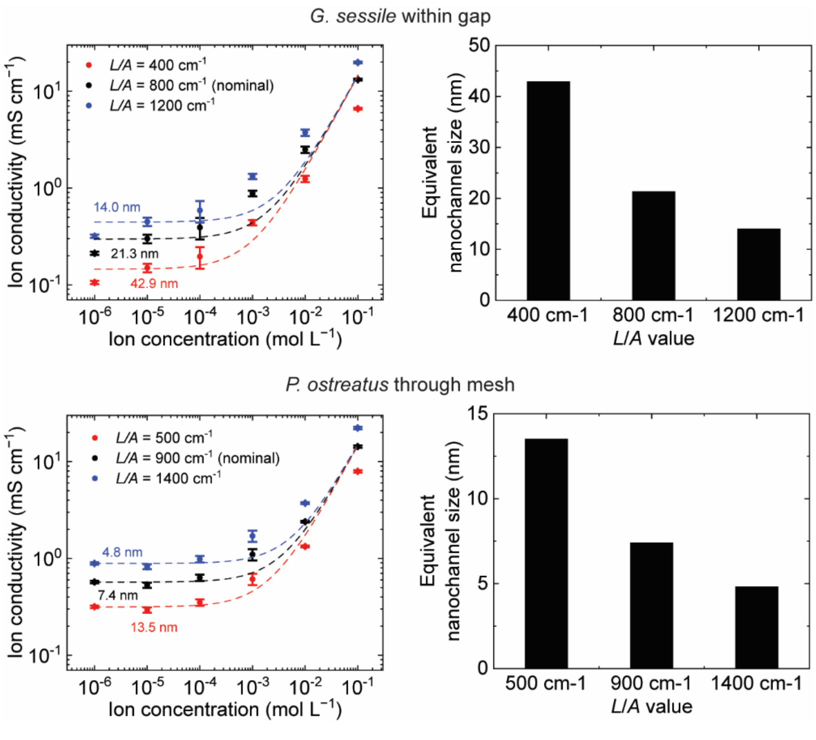

Supplementary Figure 1. Sensitivity analysis based on estimated range of the geometric parameter *L*/*A*. The ion conductivity varies linearly with the *L*/*A* value, while the equivalent nanochannel size is inversely proportional to the *L*/*A* value as indicated by Equation S2. However, the results remain within the same order of magnitude across the estimated geometric range. Error bars come from Figure 3k and Figure 3n.

Supplementary Note 5. Analysis of fluorescent images

The dilute Ca^{2+} solution is confined within the microchannel. Therefore, the fluorescence is expected to originate exclusively from the microchannel region. However, at a Ca^{2+} concentration of 10^{-5} mol L^{-1} with Fluo-4FF (Figure 4j bottom right), the region outside the microchannel also appears bright. This apparent background elevation arises from optical scattering and out-of-focus fluorescence associated with the strong fluorescence inside the microchannel rather than from genuine fluorescence outside the channel. To quantitatively analyze the fluorescence induced by nanofluidic Ca^{2+} enrichment, the measured green channel intensity in each image is expressed as

$$I_{\text{reading}} = I_{\text{dark}} + I_{\text{optical background}} + I_{\text{true fluorescence}} \tag{S3}$$

where I_{dark} represents the camera dark signal, $I_{\text{optical background}}$ accounts for the background elevation caused by optical scattering of in-channel fluorescence, and $I_{\text{true fluorescence}}$ corresponds to the genuine Ca^{2+}-dependent fluorescence signal. Within a given image, regions inside and outside the microchannel are sufficiently close to share the same I_{dark} and $I_{\text{optical background}}$. Consequently, the difference between the average green channel intensities measured inside the microchannel and in the off-channel background directly yields $I_{\text{true fluorescence}}$. The within-microchannel value corresponds to the maximum intensity measured within the microchannel region, while the outside-of-microchannel value corresponds to the average intensity measured in the background region. The enhancement in the background-subtracted net fluorescence intensity ($I_{\text{true fluorescence}}$, with hyphae vs. without hyphae) is used to quantify the sensitivity enhancement as summarized in Supplementary Table 1.

Furthermore, the signal-to-noise ratio (SNR) is defined as

$$\text{SNR} = I_{\text{true fluorescence}}/\sigma_{\text{background}} \tag{S4}$$

where $\sigma_{\text{background}}$ is the standard deviation of the background intensity. The presence of the nanofluidic hyphal network increases the SNR from 2.3 to 99.7 and from 3.5 to 79.0 for the two dilute Ca^{2+} solutions, respectively. At 10^{-5} mol L^{-1} Ca^{2+} concentration, the SNR is only slightly above 3 without hyphae, whereas the SNR is significantly improved in the presence of hyphae even at a lower concentration of 10^{-6} mol L^{-1}. This result indicates that the mycelial scaffold improves the effective limit of detection (LOD) by approximately one order of magnitude.

Supplementary Table 1. Green channel intensity for four cases in Figure 4j–l

Green color intensity (a.u.)		Within-microchannel	Out-of-microchannel	True fluorescence	Enhancement	Background noise	SNR
10^{-6} mol L^{-1}	w/o hyphae	37.3	35.5	1.8	55×	0.79	2.3
	w/ hyphae	136.6	38.0	98.6		0.99	99.7
10^{-5} mol L^{-1}	w/o hyphae	54.1	50.2	3.9	24×	1.13	3.5
	w/ hyphae	184.0	92.1	91.9		1.16	79.0

Supplementary Figures

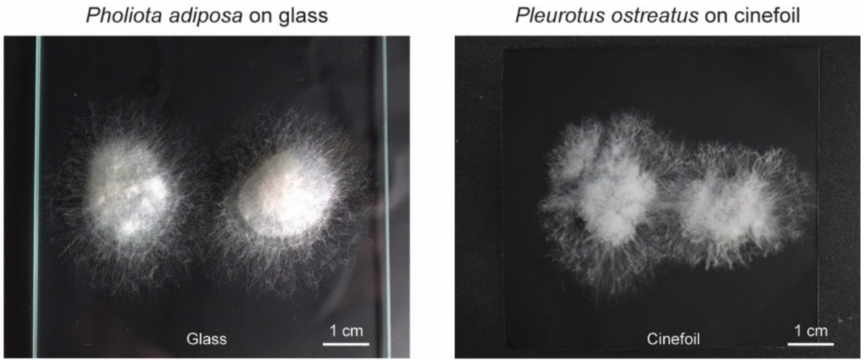

Supplementary Figure 2. Hyphal fusion of mycelium. Mycelium growing from two agar-based nutrient sources spreads out and fuses via through hyphal entanglement and fusion, also known as anastomosis. The two substrates (glass and cinefoil) demonstrate the mycelium's adaptability to different surfaces.

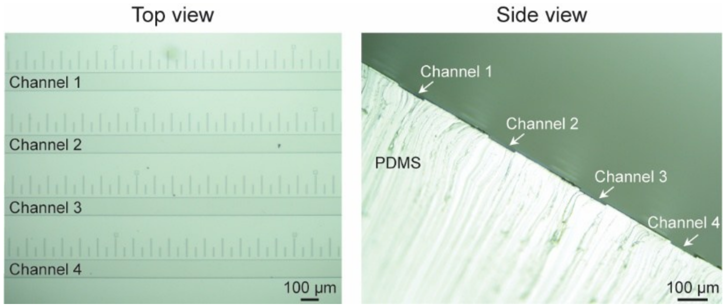

Supplementary Figure 3. Top-view and side-view microscope images of the microchannels in a microfluidic device, fabricated using a silicone template.

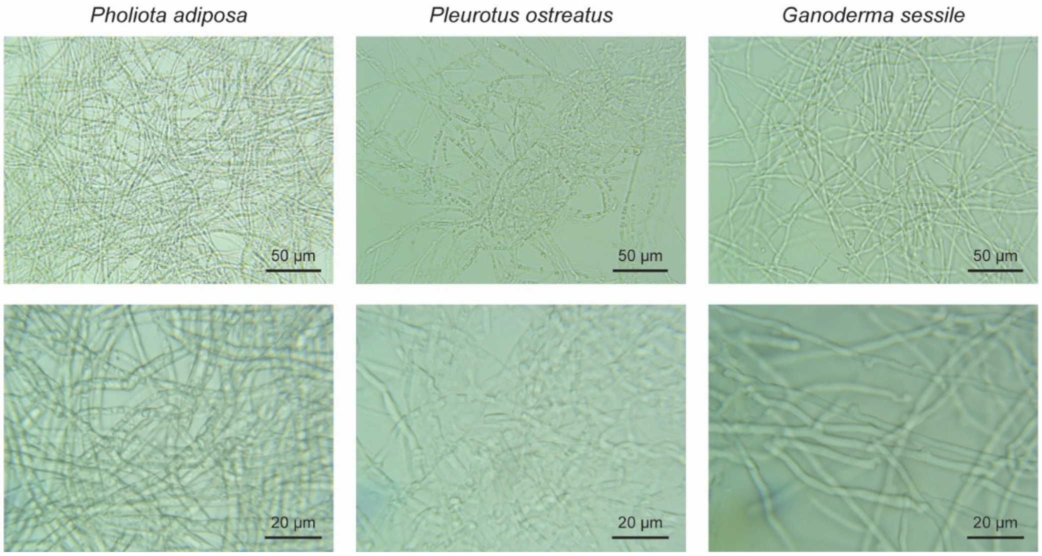

Supplementary Figure 4. Microscope images of three mycelium species in the live state, showing a universal fibrous network with fiber diameters ranging from 1 μm to 4 μm.

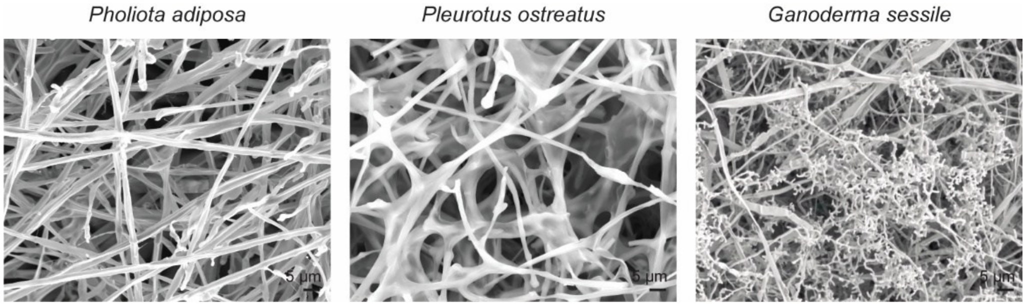

Supplementary Figure 5. Zoom-in SEM images of three mycelium species, showing clearly distinct hyphal morphologies.

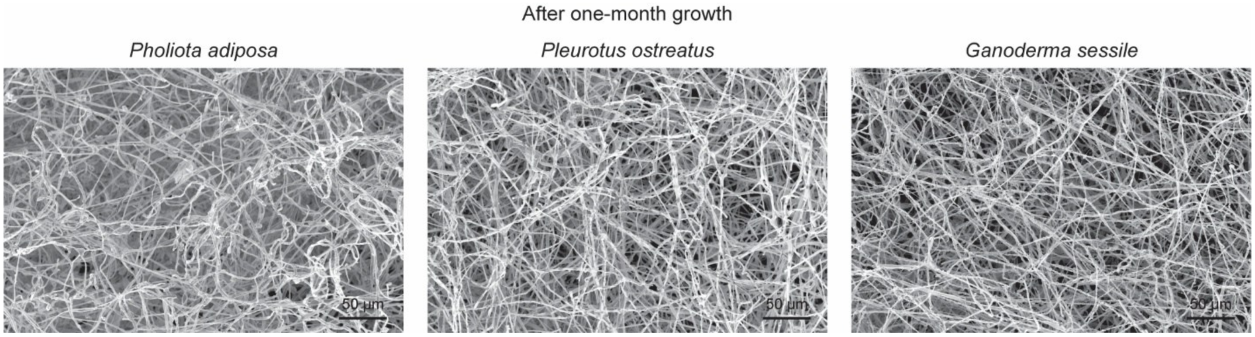

Supplementary Figure 6. SEM images of three mycelium species at the mature stage after one-month growth, showing no obvious differences in hyphal morphologies.

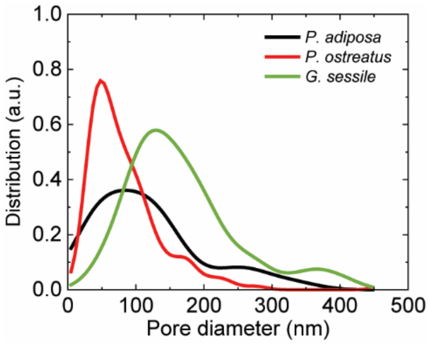

Supplementary Figure 7. Pore size distribution of hyphal cross-sections from three mycelium species, demonstrating their nanoporous properties. The distribution is derived from SEM images in Figure 2c using ImageJ.

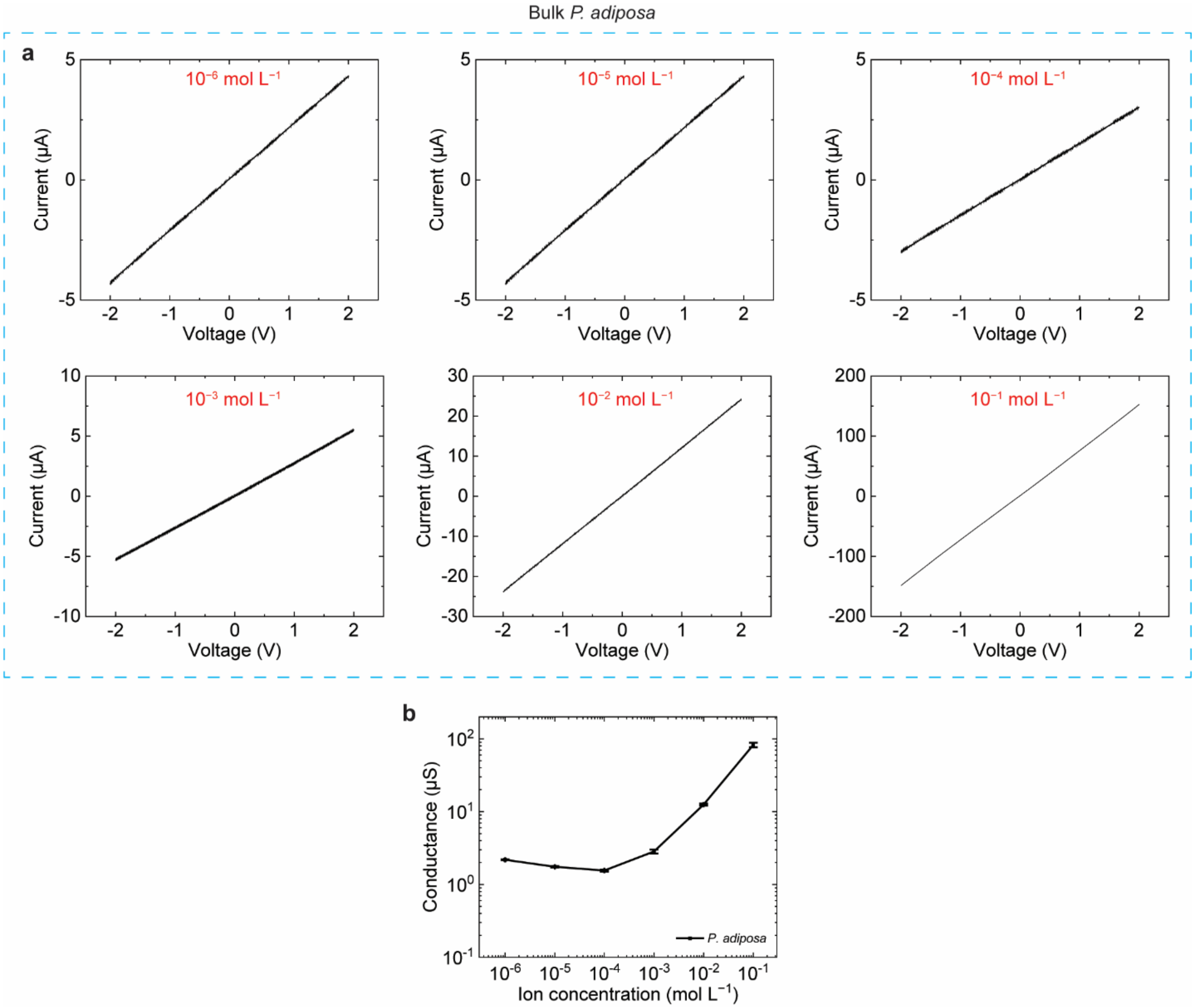

Supplementary Figure 8. I–V characterization of the bulk *P. adiposa* mycelium. (a) Representative I–V curves. (b) Conductance derived from the I–V curves. Full raw data are provided in the source data file. Error bars represent the standard deviation of five samples from independently grown patches.

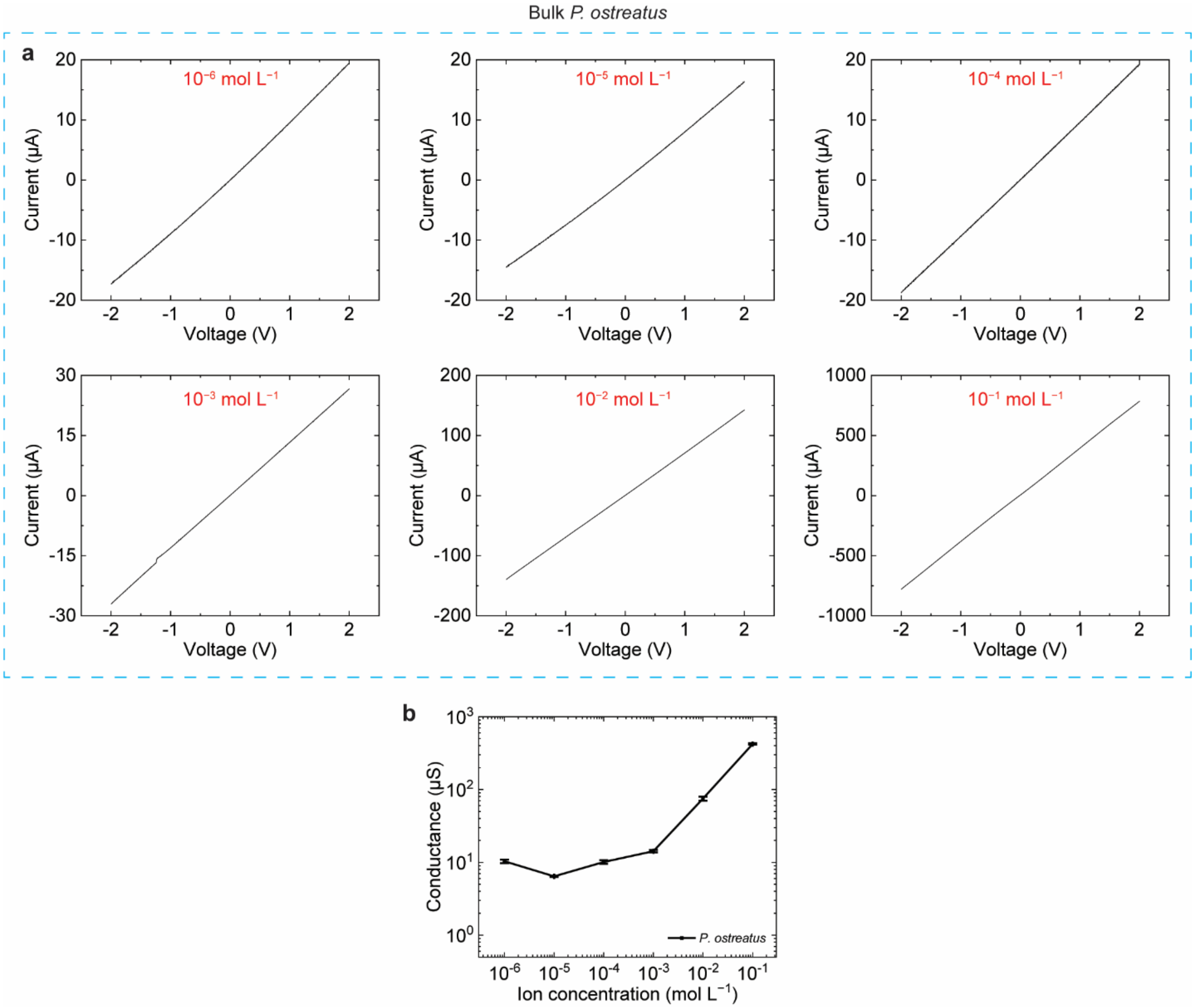

Supplementary Figure 9. I–V characterization of the bulk *P. ostreatus* mycelium. (a) Representative I–V curves. (b) Conductance derived from the I–V curves. Full raw data are provided in the source data file. Error bars represent the standard deviation of five samples from independently grown patches.

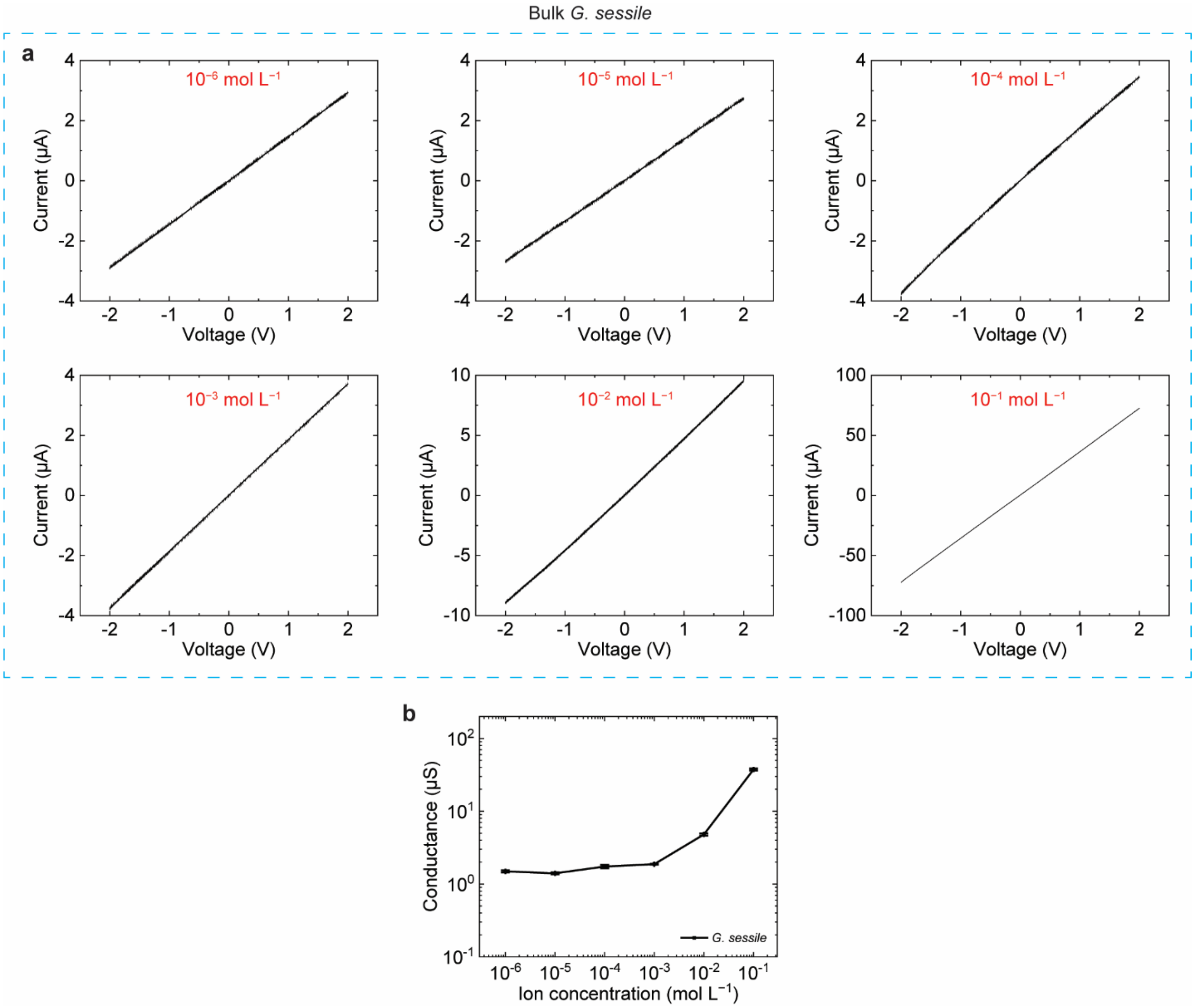

Supplementary Figure 10. I–V characterization of the bulk *G. sessile* mycelium. (a) Representative I–V curves. (b) Conductance derived from the I–V curves. Full raw data are provided in the source data file. Error bars represent the standard deviation of five samples from independently grown patches.

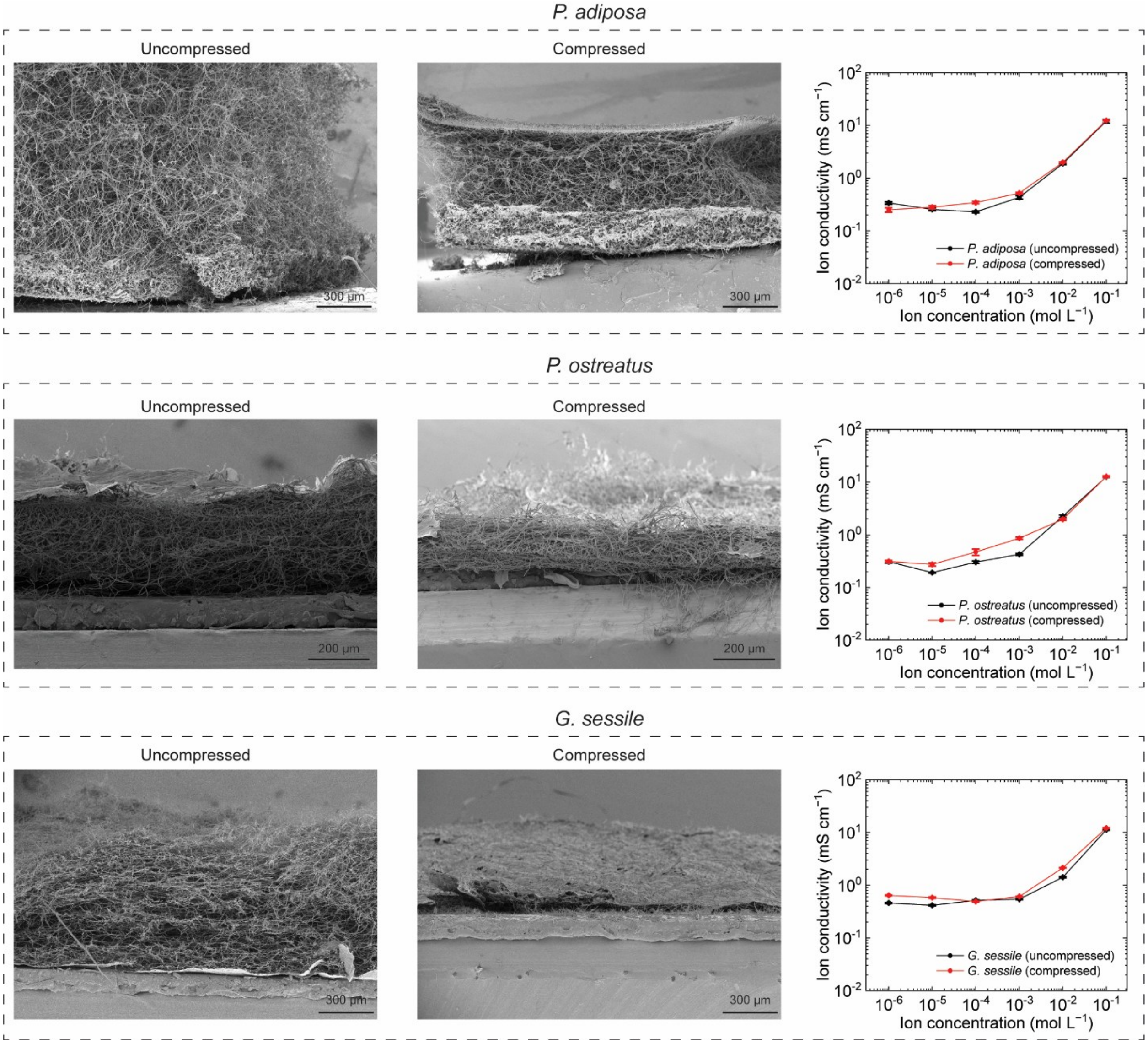

Supplementary Figure 11. SEM images of original (uncompressed) and compressed mycelium samples, together with the corresponding ion conductivity measurements. The compressed samples were subjected to a pressure of 6.9 MPa for 1 min, resulting in reduced void volume between hyphae. The comparable conductivity observed for uncompressed and compressed samples suggests that the ion transport primarily occurs through the nanoconfined hyphal network rather than through microscale void spaces between adjacent hyphae. Error bars represent the standard deviation of measurements from three samples.

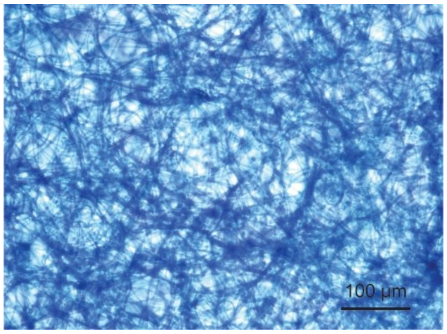

Supplementary Figure 12. Optical microscope image of the *P. adiposa* hyphal network stained with 1×10^{-4} mol L^{-1} methylene blue solution for dye-tracing experiments. The uniform staining indicates that the mycelial hyphae form an interconnected network with continuous transport pathways.

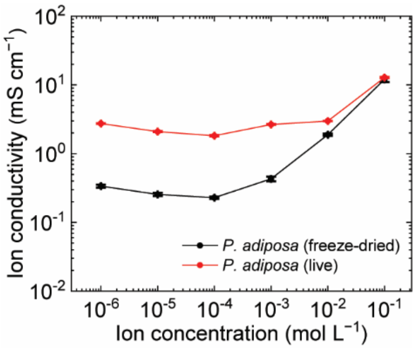

Supplementary Figure 13. Ion conductivity of *P. adiposa* mycelium measured in live and freeze-dried states. Freeze-drying preserves the nanostructured porosity of the hyphal network while removing intracellular fluids, ruling out metabolically driven transport processes and enabling clearer observation of nanofluidic ion transport through the mycelial network. Error bars represent the standard deviation of measurements from three samples.

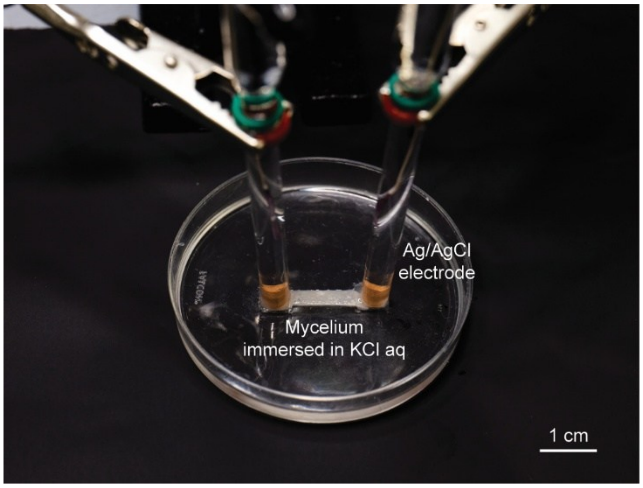

Supplementary Figure 14. Experimental setup for ion conductivity measurements. The mycelium samples were first washed to remove residual ions and then immersed in a KCl solution at a specific concentration to measure their ion conductivity.

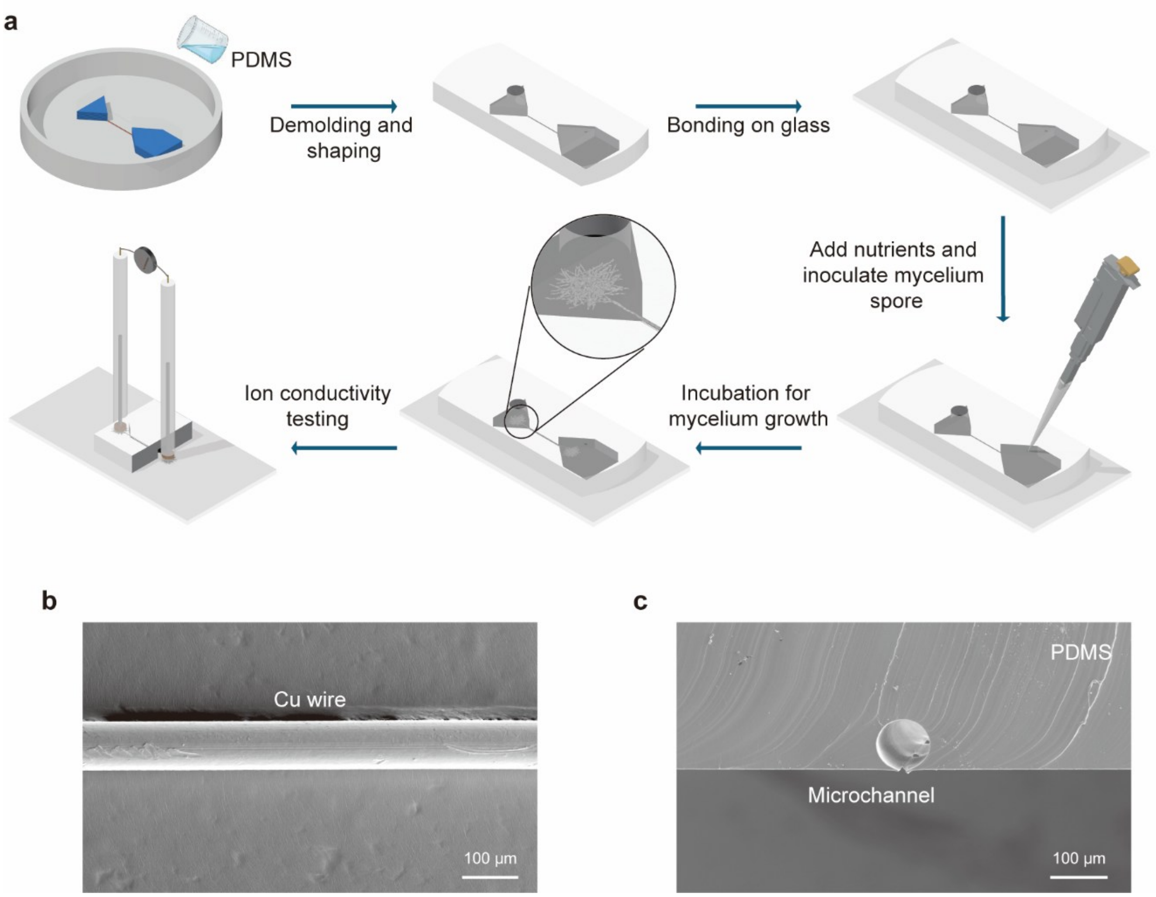

Supplementary Figure 15. Fabrication of the microchannel device using a copper wire. (a) The microchannel interconnects a reservoir containing nutrients and mycelium spores with an open space exposed to ambient air. Oxygen drives the mycelium to grow through the microchannel until it is fully filled. (b–c) SEM images of the copper wire template and the resulting microchannel embedded in PDMS.

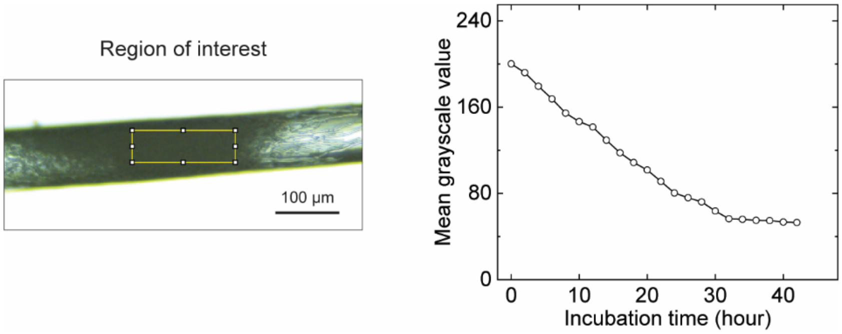

Supplementary Figure 16. Mean grayscale value measured within a fixed region of interest (ROI) in the microchannel to track *P. adiposa* hyphal growth over incubation time, analyzed using ImageJ. As incubation time increases, the denser hyphal network results in lower grayscale intensity. The filling ratio of the hyphal network is estimated to be around 5–20% by comparing the apparent density of the porous mycelial network with that of individual mycelial hyphae, as discussed in Supplementary Note 3. This filling ratio corresponds to an estimated ~50–170 hyphae spanning the microchannel cross-section. Direct experimental determination is challenging because cross-sectional cutting would disrupt the interconnected hyphal network and alter its native structure, thereby affecting accurate characterization.

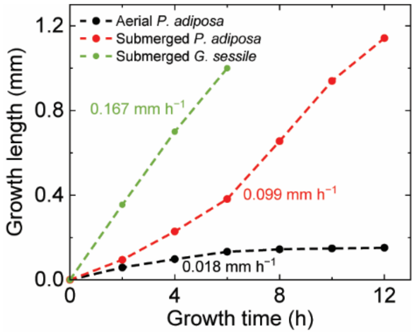

Supplementary Figure 17. Growth rates of aerial *P. adiposa* (Supplementary Movie 2), submerged *P. adiposa* (Supplementary Movie 3), and submerged *G. sessile* (Supplementary Movie 1) in microchannels. Submerged mycelium grows faster, with growth rates varying across species.

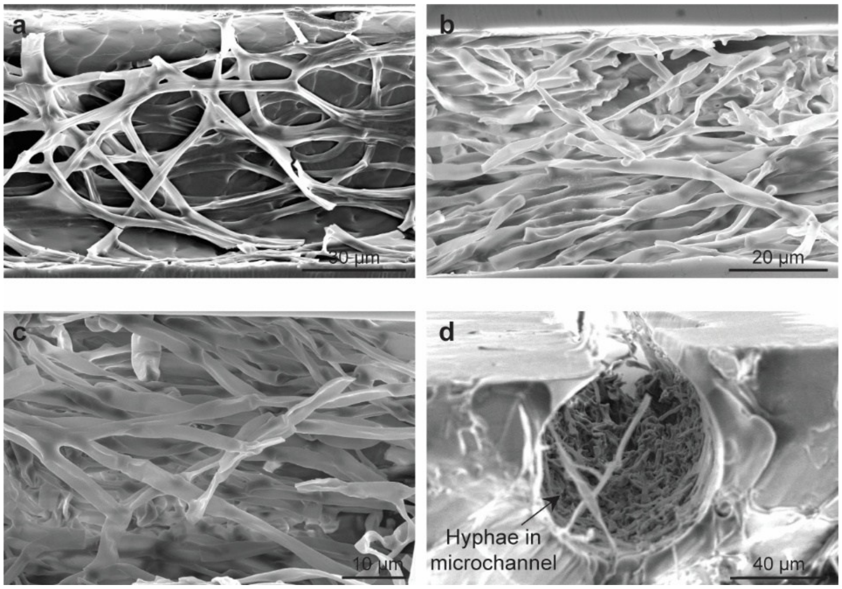

Supplementary Figure 18. SEM images of self-grown *P. adiposa* in the microchannel, forming an interwoven hyphal network that enables functional nanofluidic ion transport. The hyphal network fills the microchannel, as shown by the optical microscope images (Figure 3d). It is noted that the SEM images here do not clearly show the fully filled structure because part of the hyphal network can be mechanically disturbed or removed when the PDMS microchannel is detached from the glass substrate for SEM imaging.

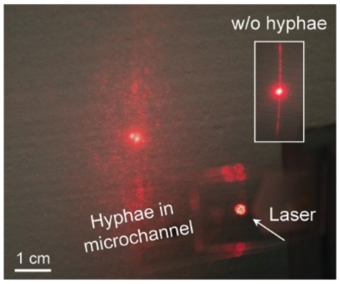

Supplementary Figure 19. Laser diffraction pattern of hyphae in the microchannel, indicating their alignment. The hyphae in the microchannel are oriented from left to right. The laser module used is a VLM-635-63 LPT-100 with a spot size of ~0.03 mm.

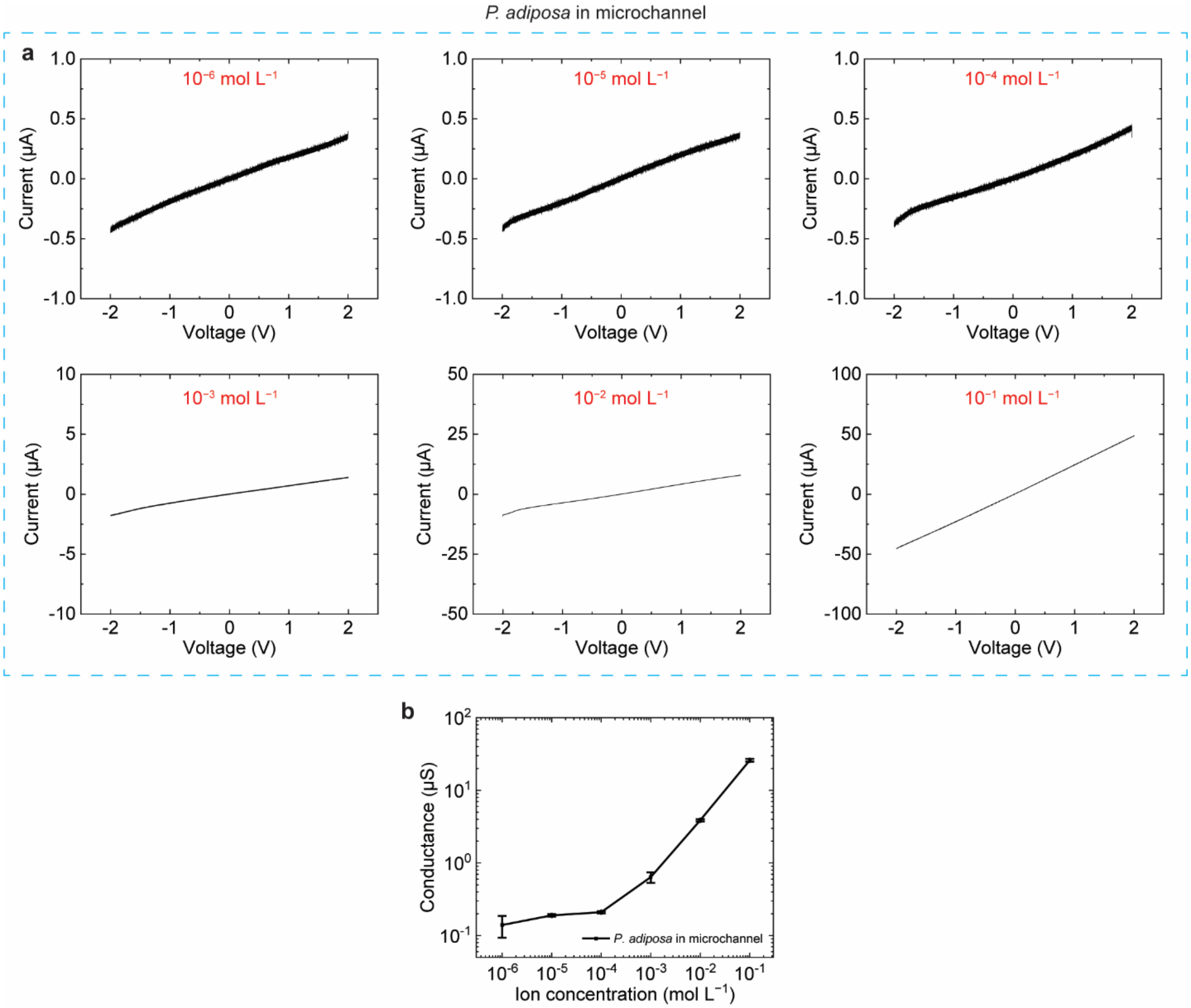

Supplementary Figure 20. I–V characterization of the *P. adiposa* hyphal network in the microchannel, clearly demonstrating nonlinearity due to nanofluidic properties. (a) Representative I–V curves. (b) Conductance derived from the I–V curves. Full raw data are provided in the source data file. The I–V curves are symmetric under forward and reverse bias, indicating that the measured conductivity is independent of the mycelial growth direction within the microchannel. Error bars represent the standard deviation of measurements from at least three devices fabricated from independently grown patches.

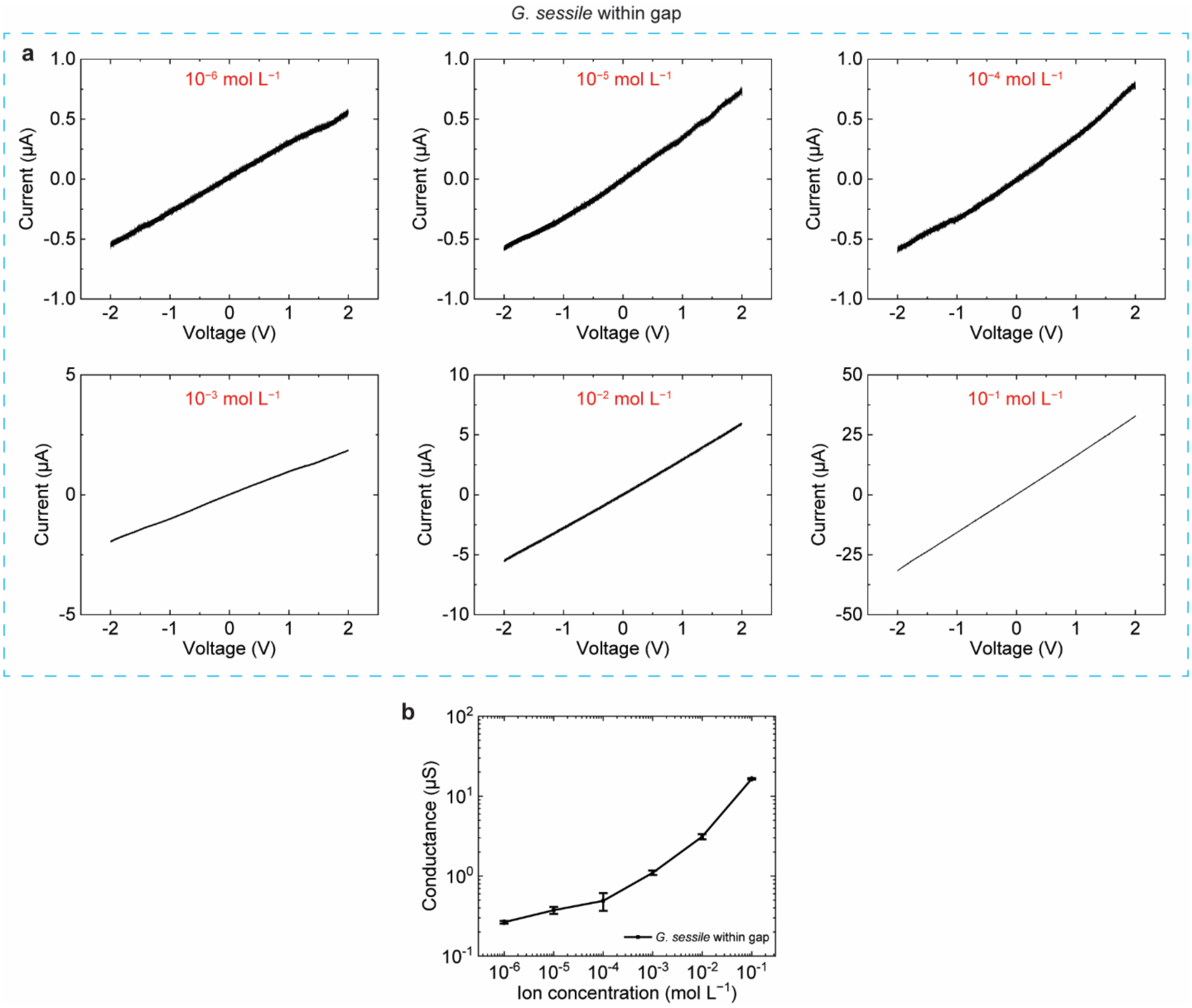

Supplementary Figure 21. I–V characterization of the *G. sessile* hyphal network within the gap between the glass pieces, clearly demonstrating nonlinearity due to nanofluidic properties. (a) Representative I–V curves. (b) Conductance derived from the I–V curves. Full raw data are provided in the source data file. Error bars represent the standard deviation of measurements from at least three devices fabricated from independently grown patches.

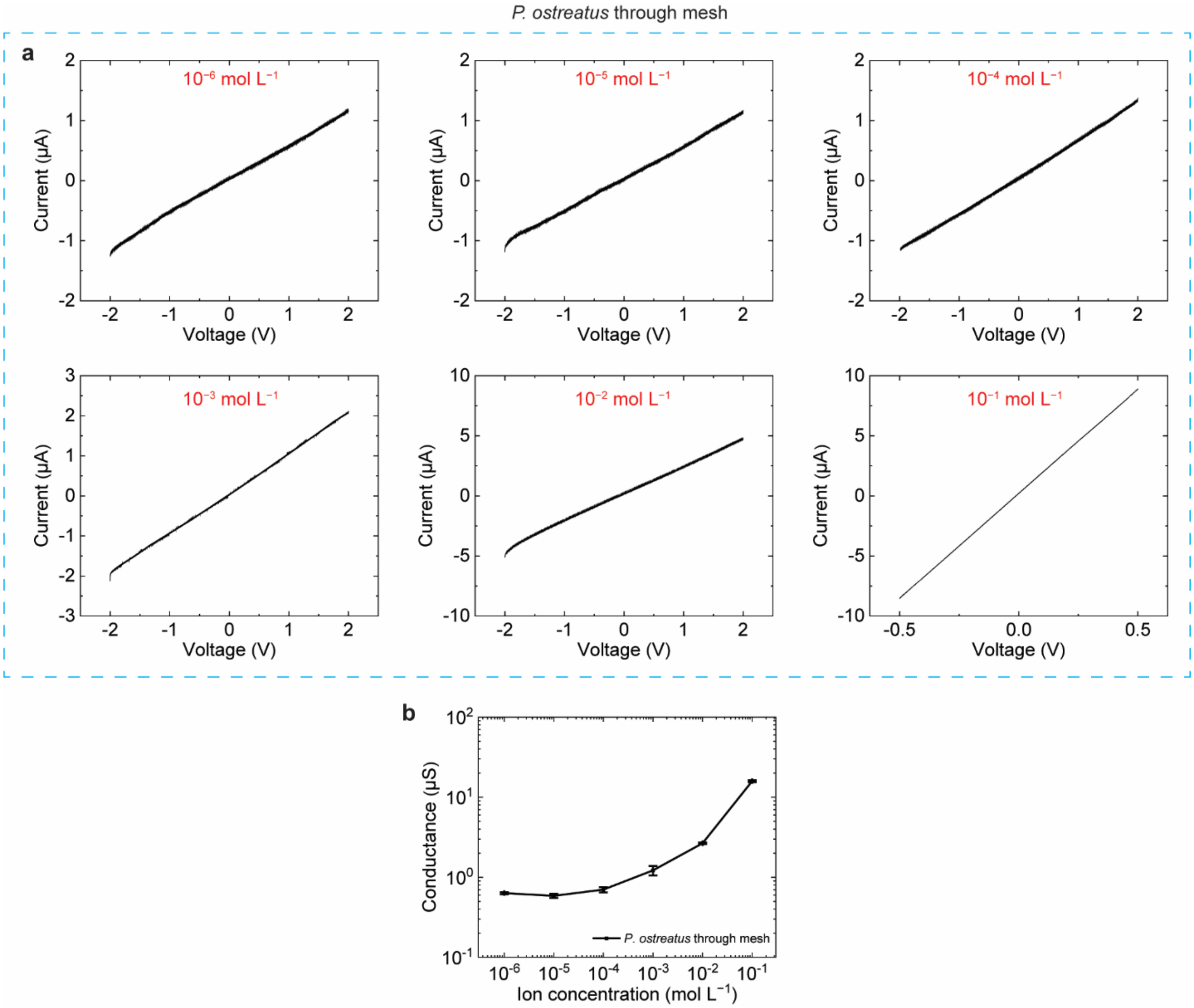

Supplementary Figure 22. I–V characterization of the *P. ostreatus* hyphal network through the metal mesh, clearly demonstrating nonlinearity due to nanofluidic properties. (a) Representative I–V curves. (b) Conductance derived from the I–V curves. Full raw data are provided in the source data file. Error bars represent the standard deviation of measurements from at least three devices fabricated from independently grown patches.

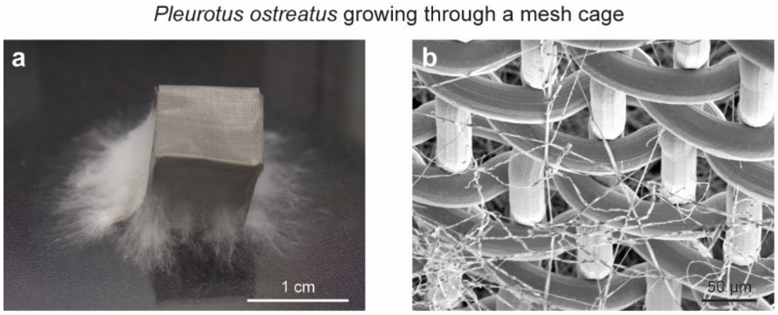

Supplementary Figure 23. Photo and SEM image of *P. ostreatus* mycelium breaking through a cage made of metal mesh (400 mesh), demonstrating its extensibility and penetration ability.

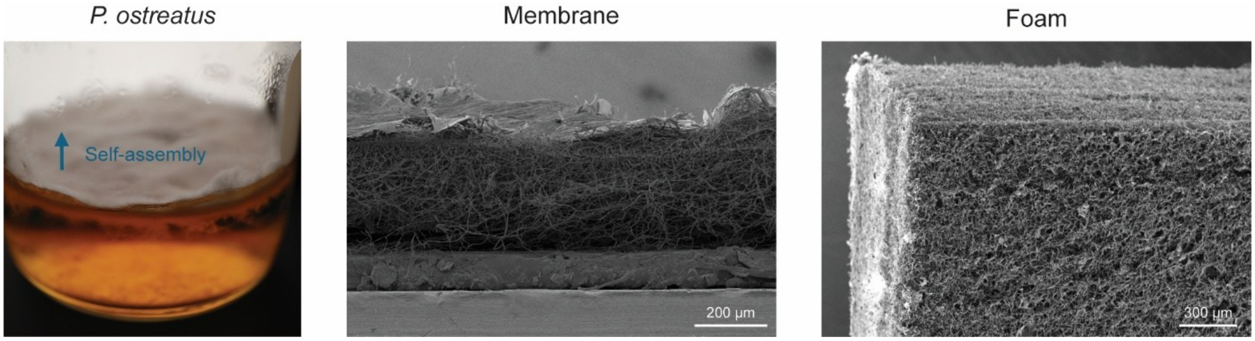

Supplementary Figure 24. Biological self-assembly of mycelium into membrane and foam structures, illustrating the dimensional versatility of the material.

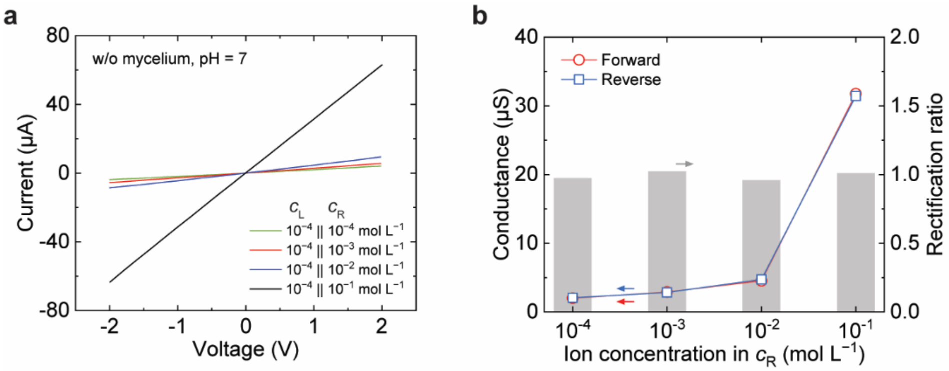

Supplementary Figure 25. Current–voltage measurements of the control device without mycelium under asymmetric bath concentrations. (a) Current–voltage response. (b) Corresponding rectification ratio, which remains close to unity, indicating the absence of clear rectification. This behavior is consistent with the lack of surface-charge-regulated ion transport in the absence of mycelial hyphae.

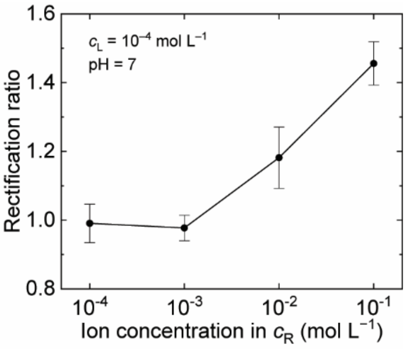

Supplementary Figure 26. Rectification ratio from –3 V to 3 V at different ion concentrations. Error bars indicate the standard deviation across three independent devices fabricated from separately grown batches.

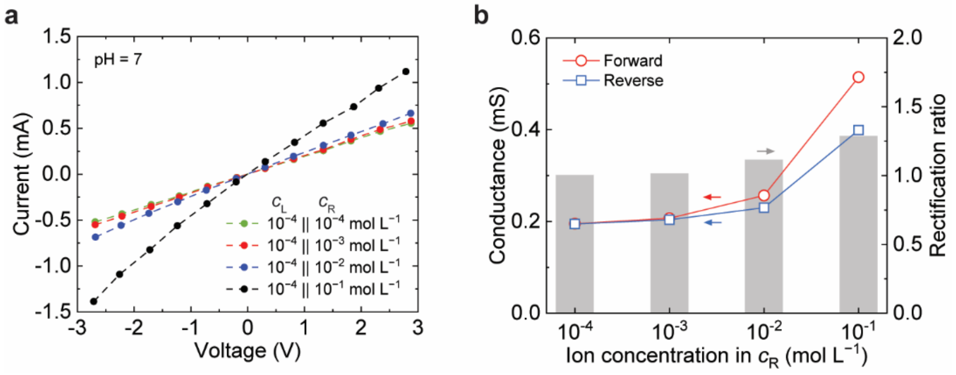

Supplementary Figure 27. Current–voltage measurements of bulk *P. adiposa* mycelium under asymmetric bath concentrations. (a) Current–voltage response. (b) Corresponding rectification ratio, reaching up to 1.3. This behavior is consistent with the device measurements shown in Figure 4e–f.

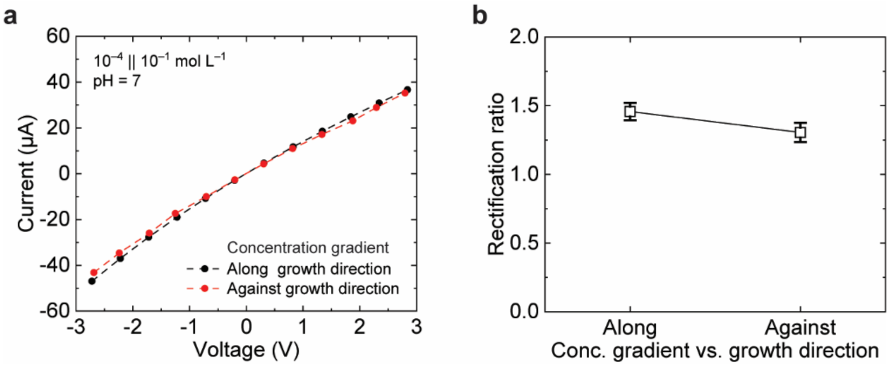

Supplementary Figure 28. Rectification behavior for concentration gradients applied along or opposite to the mycelial growth direction. (a) Current–voltage response. (b) Corresponding rectification ratio, showing negligible difference between the two directions. This behavior is consistent with the fibrous network structure of mycelium, composed of numerous interconnected hyphae that reduce structural anisotropy and directional asymmetry in ion transport. Error bars represent the standard deviation of measurements from three devices fabricated from independently grown patches.

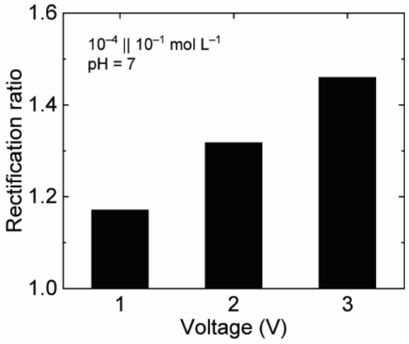

Supplementary Figure 29. Rectification ratio at different applied voltages. The rectification ratio increases modestly from ~1.2 at 1 V to ~1.5 at 3 V, consistent with field-driven enhancement of the asymmetric ion transport established by the concentration gradient and surface charge within the mycelial scaffold.

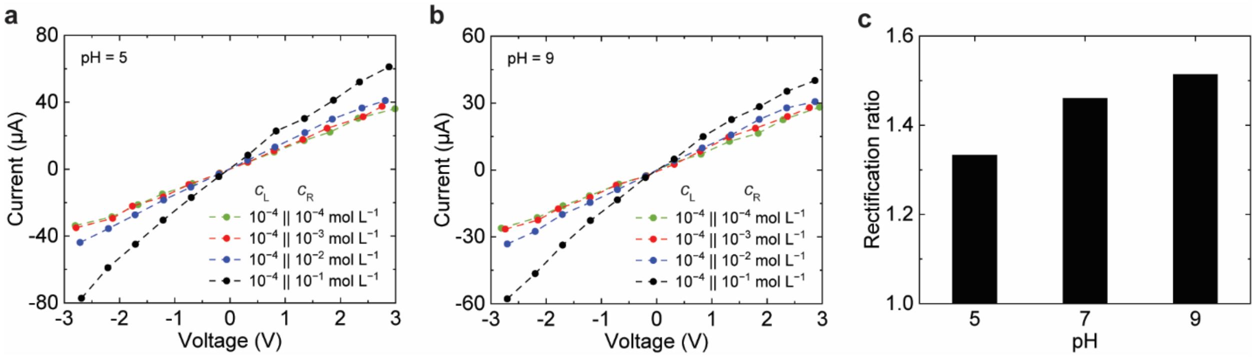

Supplementary Figure 30. Rectification behavior under different pH conditions. (a–b) Current–voltage responses measured at pH = 5 and pH = 9, respectively. (c) Corresponding rectification ratio as a function of pH. A higher pH leads to stronger rectification, consistent with increased negative surface charge of the mycelial network due to deprotonation of surface functional groups, which enhances cation-selective ion transport.

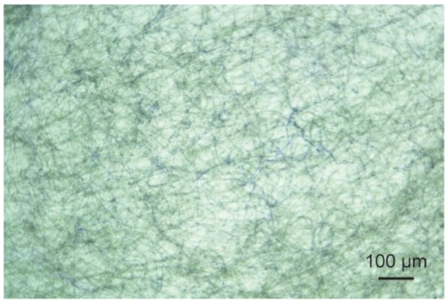

Supplementary Figure 31. Optical microscope image of the stained *P. adiposa* hyphal network after rinsing with deionized water for five minutes to remove methylene blue. The staining is significantly reduced compared with that observed before washout (Supplementary Figure 12), indicating that the signal arises from ion enrichment rather than irreversible adsorption.

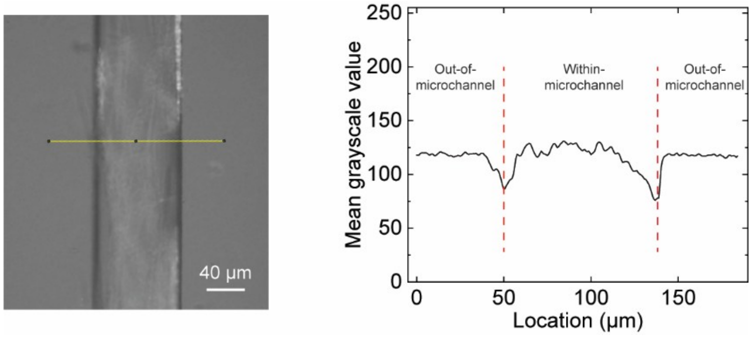

Supplementary Figure 32. Control fluorescence image of the mycelium-integrated microchannel containing the fluorescence indicator Fluo-4FF but no Ca^{2+}. The extracted grayscale image of the green channel shows no clear difference between the microchannel region and the surrounding area, likely because immersion of the mycelium in aqueous solution reduces optical scattering due to refractive index matching. The line profile shows that the grayscale value increases from ~120 out of the microchannel to ~130 within the microchannel, indicating that the scattering signal without fluorescence is ~10 in grayscale value and contributes less than ~10% of the observed fluorescence enhancement.

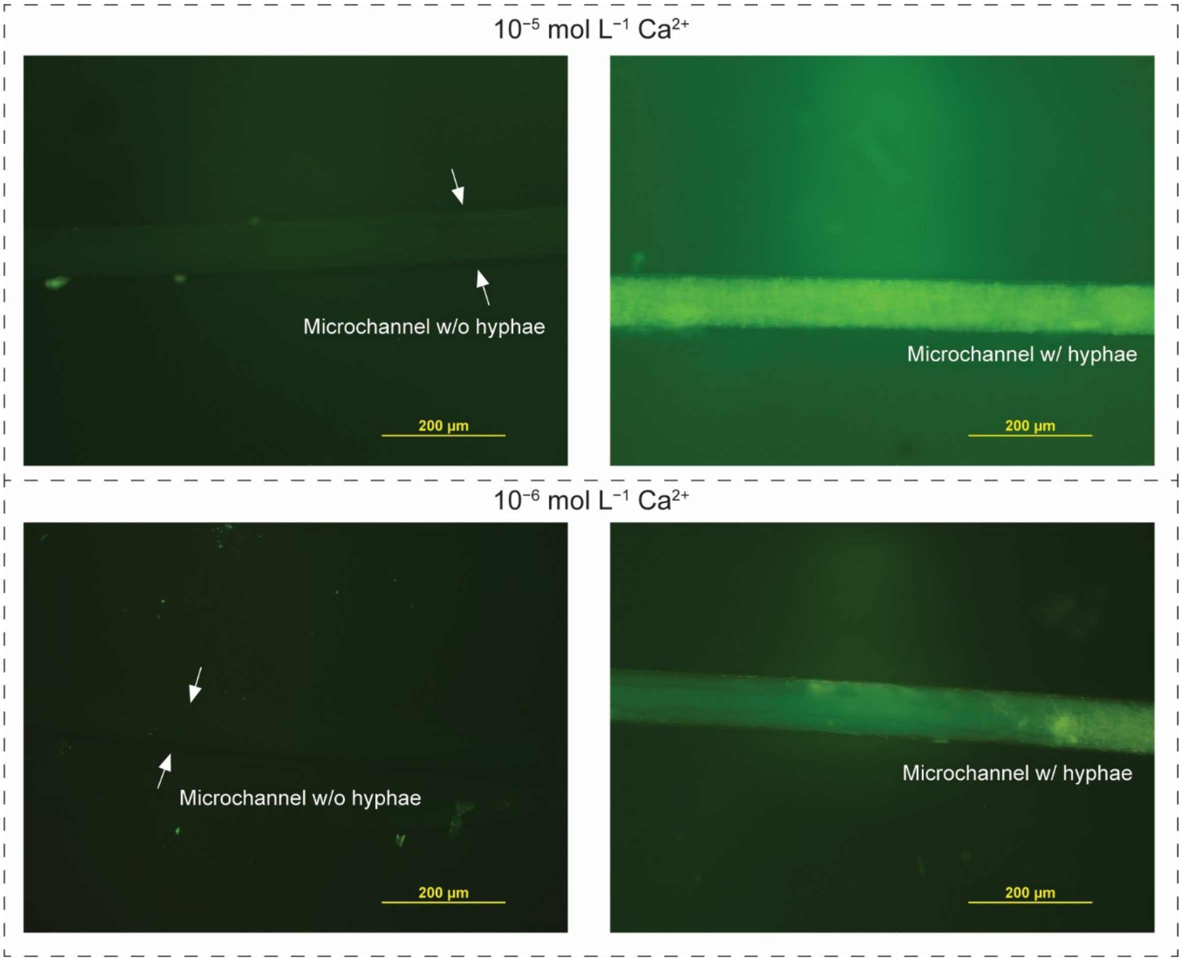

Supplementary Figure 33. Wide-field fluorescent images of the microchannel pre-soaked with Ca^{2+} immediately after exposure to Fluo-4FF. Enhanced fluorescence is observed in regions containing the mycelial network, consistent with nanofluidic cation enrichment within nanoconfined hyphal pathways.

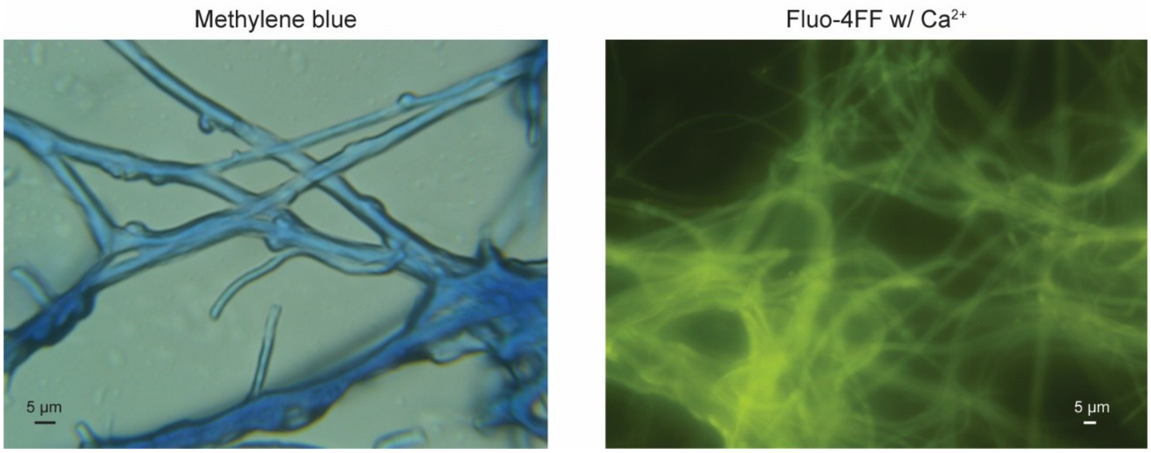

Supplementary Figure 34. Microscope images of stained *P. adiposa* mycelial hyphae. The dye signal follows the filamentous hyphal morphology and appears distributed across the hyphal width rather than being confined to the outer boundary, consistent with local cation enrichment within nanoconfined hyphal pathways rather than simple surface adsorption.

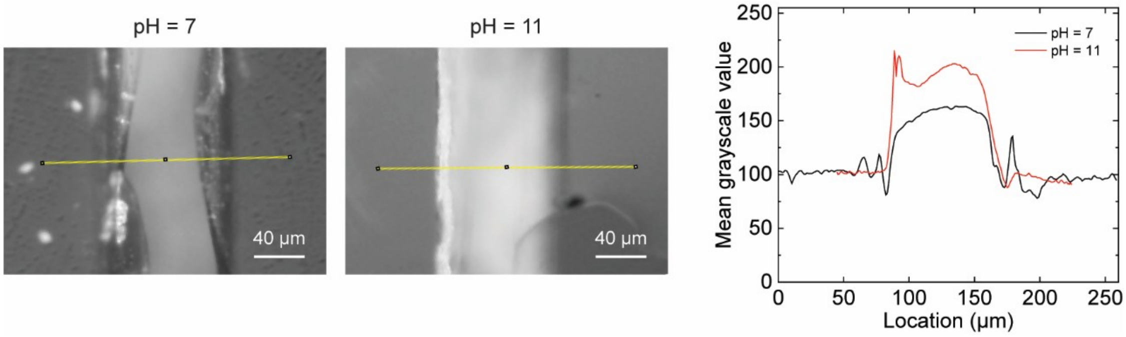

Supplementary Figure 35. Fluorescence images of the mycelium-integrated microchannel under different pH conditions. The mycelial hyphae appear out of focus due to the cylindrical geometry of the microchannel. Increasing pH enhances the negative surface charge of the mycelial scaffold, leading to stronger cation attraction and increased fluorescence intensity, as observed from the grayscale values. This behavior is consistent with surface-charge-regulated ion enrichment rather than partitioning. The pH was adjusted by adding KOH solution, which also introduces additional cations that may interact with the mycelial scaffold.

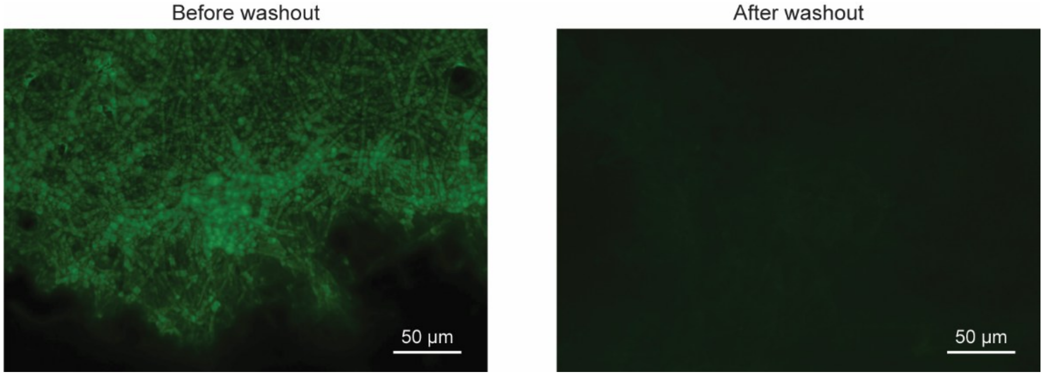

Supplementary Figure 36. Fluorescence image of the Ca^{2+} washout test after rinsing with deionized water for five minutes. As the mycelium integrated in the channel is difficult to rinse due to its embedded structure, bulk *P. adiposa* mycelium was used for the Ca^{2+} washout test. The significantly reduced fluorescence after washout indicates that the fluorescence enhancement in mycelium is unlikely to arise from ion adsorption.

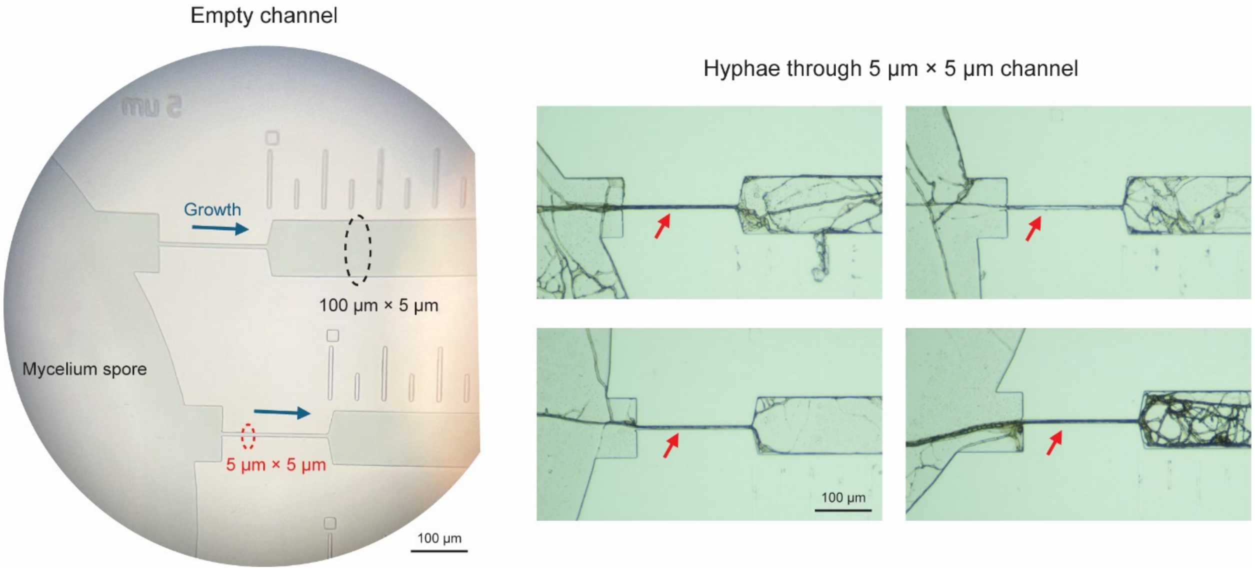

Supplementary Figure 37. Demonstration of *Lactarius* mycelium hyphae growing through a 5 μm × 5 μm microchannel. Mycelium spores were inoculated into the left chamber of the microfluidic device, and the resulting hyphae extended through the 5 μm × 5 μm channel and subsequently proliferated in the microchannel of 100 μm width. This result demonstrates that mycelium can successfully grow through single-digit micrometer-scale channels, supporting the feasibility of single-hypha nanofluidic devices.

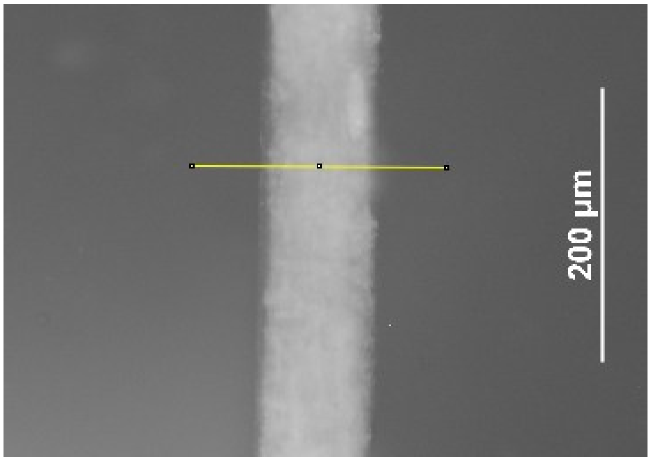

Supplementary Figure 38. Region of interest (ROI) used for intensity extraction in the green-channel grayscale image given by ImageJ.

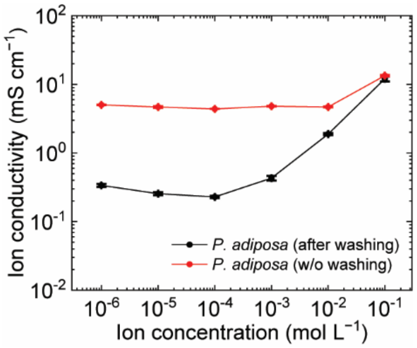

Supplementary Figure 39. Ion conductivity of *P. adiposa* mycelium with and without the washing procedure. Without washing, residual nutrients, pre-existing ions, and materials released from the hyphae can mask the intrinsic nanofluidic ion transport behavior. Error bars represent the standard deviation of measurements from three samples.

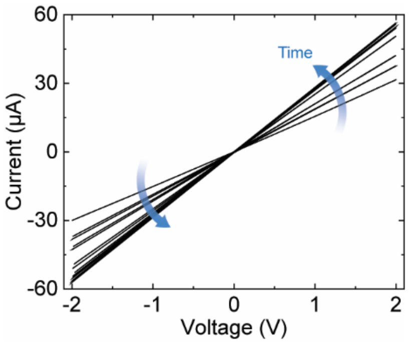

Supplementary Figure 40. Transient current–voltage response of *P. adiposa* mycelium immersed in 1×10^{-4} mol L^{-1} KCl solution. The ion transport shows a delayed response due to diffusion-limited equilibration within the mycelium. Therefore, in our measurements the samples were immersed in the KCl electrolyte for at least 24 hours prior to testing to ensure complete electrolyte equilibration.

References in Supplementary Information

1. Butt H-J, Graf K, Kappl M. *Physics and chemistry of interfaces*. John Wiley & Sons (2023).
2. Cheng L-J. Ion and molecule transport in nanochannels. University of Michigan (2008).
3. Vanysek PJChboc, physics. Ionic conductivity and diffusion at infinite dilution. 5-92 (1993).
4. Islam MR, Tudryn G, Bucinell R, Schadler L, Picu RC. Morphology and mechanics of fungal mycelium. *Scientific Reports* **7**, (2017).
5. George K, Rodríguez-Grau G, Manikandan VS, Molina P, Sancy M, Thirumurugan A. Engineering functional mycelium-based composites: Innovations in thermal and acoustic insulation. *Composites Part B: Engineering* **315**, (2026).
