# Supplementary material for: Self-grown mycelium in confined geometries as nanofluidic devices
Source: Nat Commun. 2026 May 15;17:6461. doi: 10.1038/s41467-026-72999-0 (PMC13376398; doi:10.1038/s41467-026-72999-0)
Supplement: Supplementary file 2 — DescriptionofAdditionalSupplementaryFiles [file 41467_2026_72999_MOESM2_ESM.pdf]

Description of Additional Supplementary Files

Supplementary Movie 1: Submerged *G. sessile* in microchannel

Supplementary Movie 2: Aerial *P. adiposa* in microchannel

Supplementary Movie 3: Submerged *P. adiposa* in microchannel

Supplementary Movie 4: Aerial and submerged *P. adiposa* in microchannel
