# Supplementary material for: Self-grown mycelium in confined geometries as nanofluidic devices
Source: Nat Commun. 2026 May 15;17:6461. doi: 10.1038/s41467-026-72999-0 (PMC13376398; doi:10.1038/s41467-026-72999-0)
Supplement: Supplementary file 7 — Transparent Peer Review file [file 41467_2026_72999_MOESM7_ESM.pdf]

nature portfolio

Peer Review File

Self-Grown Mycelium in Confined Geometries as Nanofluidic Devices

Corresponding Author: Professor Tian Li

This file contains all reviewer reports in order by version, followed by all author rebuttals in order by version.

Version 0:

Reviewer comments:

Reviewer #1

(Remarks to the Author)
1. Noteworthy results

This novel manuscript introduces self-grown fungal mycelium, formed in confined geometries and then freeze-dried, as a bio-derived porous scaffold that exhibits nanofluidic-like ion-transport behaviors. The noteworthy results are:

• pH-dependent “gating” of ionic conductance with an approximately 8.8-fold change between acidic and basic conditions.
• Current rectification under highly asymmetric concentration conditions, with rectification ratio reported up to 1.5 for a 1000-fold concentration difference.
• Demonstrations of cation enrichment that improves optical readouts, including methylene blue staining and Ca2+-indicator fluorescence enhancement (55-fold and 24-fold at the two Ca2+ concentrations).

The manuscript’s strength is the multi-geometry approach (microchannels, gaps, and open volumes) showing that the scaffold can be “grown into” different form factors rather than lithographically fabricated.

2. Significance to the field and relationship to prior literature:

If validated with stronger quantification and controls, the platform could be significant to iontronics/nanofluidics and bio-derived device engineering because it proposes a scalable bottom-up route for ion-selective transport structures in complex geometries. The manuscript frames this as complementary to top-down nanofabrication approaches and to other bottom-up/soft matter strategies where geometric programmability may be limited.

The manuscript would benefit from clearer positioning relative to established work in two areas:

Charged porous media and ion-selective membranes: Surface-charge-dominated conduction at low ionic strength, pH-dependent conductance, and modest rectification are well documented in charged nanopores/nanochannels and in porous charged membranes used for ion selectivity and ionic diodes. The authors should cite representative nanofluidics and nanoporous-membrane literature (e.g., foundational discussions of ion-current rectification and surface-charge effects in nanofluidic structures, and broader reviews of nanoporous membranes/nanochannels for selective ion transport and related electrokinetic phenomena). The manuscript should then clarify what is uniquely enabled by mycelium as the active scaffold (for example: bottom-up self-growth that conforms to irregular 3D confinements without lithographic definition of nanopores, hierarchical network connectivity, and potentially low-cost, scalable fabrication).

Nanofluidic “diodes” and concentration polarization: The reported rectification ratio (~1.5) is modest relative to canonical nanofluidic diodes, where geometric/charge asymmetry and engineered gating can yield much larger rectification factors. The authors should therefore avoid implying state-of-the-art rectification performance and instead frame novelty around manufacturability/adaptability (self-grown integration across multiple geometries), robustness, and functional demonstrations (pH gating, enrichment-enhanced optical readouts). If concentration polarization is invoked, they should clarify whether it is mechanistically evidenced (e.g., by polarization signatures vs. simply concentration asymmetry) and adjust wording accordingly.

3. Do the data support the claims and conclusions?:

Largely, but several conclusions would be stronger with added controls and clearer separation of model-dependent versus model-independent inferences. The observed trends are consistent with surface-charge-influenced transport and cation enrichment in a porous scaffold, but some claims would benefit from tighter analysis and additional mechanistic validation.

3.1 “Nanofluidic device” characterization across geometries needs stronger support

For open-volume and gap configurations, the effective transport geometry is not well defined, and an effective L/A is chosen so that the inferred conductivity at 0.1 M matches the bulk electrolyte. This assumption may be reasonable as an internal normalization, but it makes absolute conductivity values (and any inferred low-concentration plateau magnitude/‘equivalent nanochannel size’) dependent on the chosen effective geometry rather than directly constrained by measured dimensions/porosity.

Recommended revisions:

Present raw conductance (G) versus concentration (and representative I-V curves) prior to conversion to conductivity, then show how inferred conductivity depends on the assumed L/A.

Provide a sensitivity analysis showing how plateau magnitude and any extracted characteristic length scale vary over physically plausible L/A bounds (constrained by device dimensions and imaging-based estimates).

Provide an independent constraint on effective cross-section/porosity (image-based estimate, mass/volume plus porosity, or bounding calculations).

Clearly distinguish conclusions based on qualitative trends (robust) versus those relying on absolute conductivity/model fits (model-dependent).

3.2 Rectification is modest and mechanism is not fully isolated

Rectification ratios up to ~1.5 under 1000-fold concentration asymmetry are modest, raising the possibility of contributions from access resistance, small geometric asymmetries, and/or concentration polarization rather than strong intrinsic permselectivity.

Recommended revisions:

Include “no mycelium” and surface-charge-reduced/neutralized controls to test whether rectification decreases as expected if surface charge is the dominant mechanism.

Report rectification ratio versus voltage and versus ionic strength and/or pH to link the effect to surface charge.

Define rectification calculation details (voltage points, polarity convention, averaging, sample size) and show reproducibility across devices.

3.3 Optical enrichment claims need controls to distinguish enrichment from adsorption/partitioning

Recommended revisions:

State explicitly (in main Methods or SI) the enhancement metric used in the SI: ROI definition and within-channel minus outside-channel background subtraction, including any normalization.

Add adsorption/retention controls (e.g., time-course washout; inert porous control of similar porosity; or chemically/passivation-treated scaffold) to bound partitioning contributions.

Show polarity switching/time dynamics: if enhancement reverses promptly with polarity and diminishes on washout, that supports enrichment; persistence would suggest adsorption/retention.

Where possible, report analytical performance (SNR, repeatability, and any LOD/LOQ shift), not fold-intensity alone.

4. Flaws in analysis, interpretation, and conclusions

None of the issues appear fatal, but they require revision for soundness and to prevent overinterpretation:

- Geometry normalization (gap/volume) makes some quantitative conclusions underdetermined without sensitivity bounds.
- “Diode” language should be moderated or more strongly substantiated given rectification magnitude.
- Some interpretations would benefit from explicit discussion of alternative explanations (access resistance, nonspecific adsorption, scattering artifacts) and how the presented controls address them.

5. Methodology and expected standards

Overall the methods appear reasonable for a proof-of-concept, and the device concept is well-motivated. The most important soundness concerns are (i) quantitative interpretation when geometry is uncertain, (ii) replication and reporting clarity for key outcomes, and (iii) controls that separate enrichment from adsorption and rectification from generic asymmetries.

6. Reproducibility and methods detail

Several methods are described clearly at a high level, including the measurement setup and analysis tools.However, the manuscript should tighten reporting in the following areas:

• Replicates: The methods note error bars from three independent devices. Each key figure should state n, what constitutes an independent device (separate inoculation/growth batch vs same batch), and whether error bars are SD or SEM.
• Conductance extraction: Define how conductance is obtained from I-V curves (fit range, linearity criteria).
• Image quantification: Provide step-by-step image processing (exact ImageJ steps, ROIs, background correction) in Methods or SI.
• Geometry-dependent conductivity: Provide bounds/estimates for effective dimensions for each configuration; if not possible, provide a sensitivity analysis (as above).

7. Transparency: data availability

The manuscript states that data are available upon reasonable request.
For Nat Comms, the authors should strongly consider depositing raw/processed datasets and analysis scripts in a public repository, or clearly justify any constraints. At minimum, include the key raw I-V datasets and image quantification outputs.

8. Minor points and presentation

• Consider adding a short schematic summarizing the three device geometries and the hypothesized transport mechanism (surface-charge dominated regime, concentration polarization) to guide readers.

Recommendation

Major revision. The concept is novel and qualitative demonstrations are promising, however the manuscript needs strengthened quantification (especially where geometry is uncertain), clearer mechanistic controls,and more explicit replicate/statistical reporting. These appear addressable with additional analysis/controls and clearer reporting, so I recommend major revision rather than rejection.

Reviewer #2

(Remarks to the Author)
In this work, the authors demonstrate a method for integrating mycelium into microstructures to form nanofluidic networks. The overall strategy is straightforward, and the guided growth of mycelium is impressive. However, a couple of issues should be addressed before publication. Most importantly, the experimental procedures are not described clearly throughout the manuscript. It is strongly recommended to add step-by-step protocols, especially for buffer exchange and washing steps, so that the experiments can be properly evaluated and reproduced. In addition, although the fluidic system is fundamentally different from a simple nanochannel, the analysis relies mainly on current responses while assuming that nanofluidic behavior has already been established.
Specific comments are provided below.

1. Fig. 2b suggests substantial void volume between hyphae, yet the manuscript appears to assume that ion transport occurs primarily through nanofluidic hyphal networks. it would be helpful to clarify why transport through extracellular voids is excluded, and why the conductivity plateau versus concentration is attributed specifically to pathways inside hyphae.
2. The manuscript does not provide time-dependent current data. For long nanofluidic channels, transient current measurements (I vs. t) under constant bias are generally expected to show delayed ion responses. Furthermore, It is recommended to discuss why no nonlinearity associated with ion concentration polarization appears in Fig. 4b.
3. In Fig. 4a–c, achieving pH 3 requires approximately 10^−3 mM HCl, which is higher than the KCl concentration used. Therefore, ionic strength is not identical across pH conditions, and the ion-strength effect in Fig. 4d–f is not isolated from pH.
4. In Fig. 4d–f, rectification is shown only over a limited range and only under a large concentration difference. The authors are encouraged to clarify whether the platform can achieve stronger rectification, or to define the most appropriate application within the current performance range.
5. In Fig. 4g–i, the connection between dye membrane penetration and nanofluidic transport across reservoirs is unclear. Furthermore, how is staining inside hyphae demonstrated while excluding possible surface staining?
6. In Fig. 4j–l, it would be helpful to clarify where and when the images were acquired (e.g., at the entrance or in the center of the microchannel). Providing a full-field view of the microchannels would also help explain why enrichment occurs at that location.

7. A control experiment at 0 mM Ca2+ is needed. it would be clearer to clarify whether Fluo-4FF and Ca2+ were injected together or separately, whether a wash step was included, the enrichment duration, and the applied voltage bias.
8. It is also recommended to demonstrate that the enrichment indeed occurs inside hyphae.
9. In Fig. S10, it remains difficult to determine whether the channel is fully filled with mycelium or only surface-covered.
10. Please explain how pore size was derived from SEM images, and whether this method is directly comparable to the size estimates in Fig. 3e, 3i, and 3l.
11. Additional detail is needed regarding DI washing and buffer exchange in these long nanofluidic systems, including how completion of washing was determined and whether residual nutrients, pre-existing ions, or materials released from hyphae could affect the results.
12. The experimental details are insufficient for reproducibility. For example, measurement parameters of the equipment, detailed device geometry, copper wire removal from the microfluidic device, and freeze-drying conditions should be provided.
13. The manuscript states that previous methods have multiple drawbacks, but the basis for each claim is not sufficiently justified. It would improve the manuscript to specify which methods are limited in geometric programmability, throughput, and scalability. It would also be helpful to clarify how the present approach improves on them.

Minor
1. In Fig. 1e, the left-to-right transformation is not easy to follow. it would be helpful to clarify whether the panels show different sections or different time points.
2. Please define the time corresponding to “Final” in Fig. 3b.
3. The microfluidic devices shown in Fig. 3a and Supplementary Fig. 8 appear slightly different.
4. It would also be clearer to state the use of KCl in the Results section, rather than only in the Methods section.

Reviewer #3

(Remarks to the Author)
This manuscript, entitled " Self-Grown Mycelium in Confined Geometries as Nanofluidic Devices" is logically structured and supported by substantial experimental data. The authors demonstrate that ion transport through fibrous networks formed within a mycelium-integrated microchannel exhibits nanofluidic characteristics. However, based on the current evidence, it remains unclear whether the results fully substantiate the broad claims stated in the abstract, particularly that " by bypassing lithography and energy-intensive processing, this bio-derived strategy creates living and self-organizing ion transport networks with broad potential in sensing, ionic computing, and energy conversion."
While the concept is interesting and potentially impactful, the present data do not yet demonstrate sufficient mechanistic understanding or controllability to justify such wide-ranging implications. Additional theoretical or experimental support would strengthen the manuscript and better align the conclusions with the evidence provided before publication in Nature Communications. The main issues that require clarification are as follows:

1. The introduction provides a thorough overview of mycelium-related studies. However, the discussion of micro-/nano-fluidic systems is relatively limited. It would help readers if the authors included a brief discussion on the history of biomimetic transport or other engineered approaches for precise control of ion and molecular transport, particularly in analogous bio-derived or bio-templated channel systems. Clarifying how the present work differs conceptually and technically from existing biomimetic ion transport platforms would help define its originality more clearly.
2. The data regarding the mycelium membrane is convincing. However, the reproducibility and controllability of mycelium growth within confined microchannels require deeper discussion. Although Fig. S9 touches on growth rate, it would be helpful to discuss the extent to which network morphology can be controlled. A more fundamental question concerns the origin of the observed ion transport: is it primarily governed by the external EDL formed around the fibrous network (as in Fig. 1e), or does ion transport occur through the interior of the hyphae (as illustrated in Fig. 1f)? A comparison between live and freeze-dried mycelium could help distinguish between these possible transport pathways and clarify the underlying mechanism.
3. In the section describing mycelium-integrated device performance (line 245), the authors state that the self-growing and self-adaptable nature of mycelium enables filling of confined spaces and opens opportunities for nanofluidic devices. However, the degree of channel filling is not clearly quantified. Since the manuscript positions this system as a self-adaptable material platform for nanofluidics, more detailed characterization would be valuable. For example, how do variations in filling density, incubation time, or growth direction affect ionic conductivity and ICR for the system? In Figure 4, does the direction of mycelium growth influence rectification behavior? Additionally, were these measurements performed on live or freeze-dried samples? It would also be important to clarify whether ion transport and nanofluidic properties change during different stages of the biological life cycle or after freeze-drying. These experimental details should be clarified.
4. The authors fabricate a relatively large microchannel (~88 µm, defined by the copper wire), which is filled by a network of multiple fibers. Can the authors estimate approximately how many individual hyphae span the channel cross-section? Furthermore, how sensitive is the ionic current or ICR changes to the confined channel geometry itself? If the channel width is reduced, does the ion transport or ICR behavior change significantly? Is there a minimum channel dimension below which mycelium cannot bridge the gap? Could a single-hypha device even be made possible? Addressing these questions would help define the scalability and practical limitations of this approach.

Version 1:

Reviewer comments:

Reviewer #1

(Remarks to the Author)
My comments have been appropriately addressed.

Reviewer #2

(Remarks to the Author)
The authors have addressed all of my concerns in the revised manuscript. I sincerely thank them for their efforts. I am happy to recommend publication in its current form.

Reviewer #3

(Remarks to the Author)
The authors have adequately addressed all the concerns raised in my initial review. I am satisfied with the revisions and recommend acceptance of the manuscript in its current form.

Open Access This Peer Review File is licensed under a Creative Commons Attribution 4.0 International License, which permits use, sharing, adaptation, distribution and reproduction in any medium or format, as long as you give appropriate credit to the original author(s) and the source, provide a link to the Creative Commons license, and indicate if changes were made.
In cases where reviewers are anonymous, credit should be given to 'Anonymous Referee' and the source.
The images or other third party material in this Peer Review File are included in the article's Creative Commons license, unless indicated otherwise in a credit line to the material. If material is not included in the article's Creative Commons license and your intended use is not permitted by statutory regulation or exceeds the permitted use, you will need to obtain permission directly from the copyright holder.
To view a copy of this license, visit https://creativecommons.org/licenses/by/4.0/

Response Letter for NCOMMS-26-004628

Manuscript Information

Manuscript ID: NCOMMS-26-004628

Title: Self-Grown Mycelium in Confined Geometries as Nanofluidic Devices

Authors: Qilong Cheng[1†], Zhenyuan Niu[1†], Bryce Waller[2], Bingyu Xia[1], Pengfei Deng[1], Yanpei Tian[1], David Warsinger[1], Zuzanna Siwy[3], Xianming Dai[4], Gregory Bonito[2,5], Tian Li[1*]

[*]Correspondence: tianli@purdue.edu

Reviewer #1 (Remarks to the Author):

1. Noteworthy results

This novel manuscript introduces self-grown fungal mycelium, formed in confined geometries and then freeze-dried, as a bio-derived porous scaffold that exhibits nanofluidic-like ion-transport behaviors. The noteworthy results are:

• pH-dependent "gating" of ionic conductance with an approximately 8.8-fold change between acidic and basic conditions.

• Current rectification under highly asymmetric concentration conditions, with rectification ratio reported up to 1.5 for a 1000-fold concentration difference.

• Demonstrations of cation enrichment that improves optical readouts, including methylene blue staining and Ca2+-indicator fluorescence enhancement (55-fold and 24-fold at the two Ca2+ concentrations).

The manuscript's strength is the multi-geometry approach (microchannels, gaps, and open volumes) showing that the scaffold can be "grown into" different form factors rather than lithographically fabricated.

Response: We thank the reviewer for the positive evaluation and for highlighting the key findings of our work. We are pleased that the reviewer recognizes the novelty of introducing self-grown fungal mycelium as a bio-derived nanofluidic scaffold and the strength of this approach in enabling nanofluidic pathways across multiple geometries.

2. Significance to the field and relationship to prior literature:

If validated with stronger quantification and controls, the platform could be significant to iontronics/nanofluidics and bio-derived device engineering because it proposes a scalable bottom-up route for ion-selective transport structures in complex geometries. The manuscript frames this as complementary to top-down nanofabrication approaches and to other bottom-up/soft matter strategies where geometric programmability may be limited.

Response: We thank the reviewer for recognizing the potential of this platform for iontronics and nanofluidics. To improve quantification and strengthen the experimental controls, we have

clarified the device geometry and added additional rectification measurements and fluorescence experiments, together with expanded discussion in the revised manuscript. Detailed point-by-point responses to the reviewer's comments are provided below.

The manuscript would benefit from clearer positioning relative to established work in two areas: Charged porous media and ion-selective membranes: Surface-charge-dominated conduction at low ionic strength, pH-dependent conductance, and modest rectification are well documented in charged nanopores/nanochannels and in porous charged membranes used for ion selectivity and ionic diodes. The authors should cite representative nanofluidics and nanoporous-membrane literature (e.g., foundational discussions of ion-current rectification and surface-charge effects in nanofluidic structures, and broader reviews of nanoporous membranes/nanochannels for selective ion transport and related electrokinetic phenomena). The manuscript should then clarify what is uniquely enabled by mycelium as the active scaffold (for example: bottom-up self-growth that conforms to irregular 3D confinements without lithographic definition of nanopores, hierarchical network connectivity, and potentially low-cost, scalable fabrication).

Response: We thank the reviewer for this valuable suggestion on positioning our work. We have supplemented citations of representative literature on nanopores, nanochannels, and nanoporous membranes that exhibit ion selectivity, ion-current rectification, and surface-charge-governed transport. We have also clarified the advantages of the proposed mycelium platform in the revised manuscript.

We have revised in the introduction: "In many of these nanofluidic platforms, surface-charge-governed ion transport phenomena such as selectivity4, 5, rectification6, 7, and pH-dependent conductance29, 30 have been widely investigated. These effects are commonly observed in charged nanopores12, 22, 29, nanochannels31, 32, 33, and nanoporous membranes4, 12, 34 used for ion-selective transport. However, these systems are typically based on predefined nanoscale architectures or synthetic nanoporous matrices fabricated through lithography or membrane assembly, which limits their ability to autonomously generate interconnected ion transport pathways within irregular three-dimensional confinements. Overcoming these constraints calls for alternative fabrication paradigms that simultaneously deliver nanofluidic functionality, scalability, and geometric adaptability.

Biomaterials offer a promising opportunity for constructing microfluidic and nanofluidic networks35, 36, 37 owing to their intrinsic hierarchical architectures and growth-enabled connectivity. A variety of bio-derived or bio-templated transport systems have been explored to guide molecular and ionic transport, including porous membranes fabricated from natural biomaterials37, 38, transport pathways formed within bacterial biofilms39, 40, and vascular transport architectures found in plant tissues such as leaf veins41. However, most of these approaches rely on static biological templates or processed biomaterials and therefore do not exploit the intrinsic growth capability of living systems to autonomously generate interconnected transport pathways. Here, we introduce fungal mycelium as a living, low-cost, and scalable bio-derived scaffold for constructing nanofluidic pathways that can adapt to confined geometries without lithographic definition of nanoscale features. This capability arises from its bottom-up self-growth, hyphal branching and anastomosis, hierarchical porosity, and negatively charged surfaces." (page 2 line 61)

Nanofluidic "diodes" and concentration polarization: The reported rectification ratio (~1.5) is modest relative to canonical nanofluidic diodes, where geometric/charge asymmetry and engineered gating can yield much larger rectification factors. The authors should therefore avoid implying state-of-the-art rectification performance and instead frame novelty around manufacturability/adaptability (self-grown integration across multiple geometries), robustness, and functional demonstrations (pH gating, enrichment-enhanced optical readouts). If concentration polarization is invoked, they should clarify whether it is mechanistically evidenced (e.g., by polarization signatures vs. simply concentration asymmetry) and adjust wording accordingly.

Response: We thank the reviewer for the suggestion regarding the modest rectification performance. We have revised the abstract and the main text to avoid implying state-of-the-art rectification and instead emphasize the key advantages of the mycelium platform, including the bio-manufacturability, geometric adaptability, and functional demonstrations such as pH gating and cation enrichment, as detailed below.

"Specifically, a mycelium-integrated microchannel achieves a pH-gating switch ratio of up to 3.0 and a 55-fold enrichment for dilute cation detection. These results establish the principle that

nanofluidic functionality can be biologically grown rather than fabricated, introducing a scalable, sustainable, and geometrically adaptable platform." (page 1 line 27)

"The forward and reverse conductances exhibit a modest rectification ratio of 1.46±0.06 (Figure 4f and Supplementary Figure 26), which is consistent with that observed in bulk mycelium (Supplementary Figure 27). This rectification arises from the 1000-fold electrolyte concentration asymmetry across the device combined with surface-charge-governed ion transport within the mycelial scaffold." (page 14 line 337)

We also thank the reviewer for the suggestion regarding the wording of the rectification performance. We have clarified in the revised manuscript that the observed rectification arises from the asymmetric electrolyte concentrations across the device, as detailed below.

"Utilizing the inoculated hyphal network, the mycelium device exhibits typical nanofluidic properties, including pH gating (Figure 4a–c) and concentration-asymmetric rectification (Figure 4d–f)." (page 14 line 322)

"This rectification arises from the 1000-fold electrolyte concentration asymmetry across the device combined with surface-charge-governed ion transport within the mycelial scaffold. The I–V curves do not show nonlinearity associated with concentration polarization, likely because ion transport occurs through a distributed mycelial network rather than a single long nanochannel. The interconnected hyphal pathways provide multiple parallel ion transport routes with relatively large effective diameters, which reduces the formation of strong ion depletion zones and suppresses the development of concentration polarization, resulting in moderate rectification." (page 14 line 339)

3. Do the data support the claims and conclusions?:

Largely, but several conclusions would be stronger with added controls and clearer separation of model-dependent versus model-independent inferences. The observed trends are consistent with surface-charge-influenced transport and cation enrichment in a porous scaffold, but some claims would benefit from tighter analysis and additional mechanistic validation.

Response: We thank the reviewer for the constructive comment. To strengthen our conclusions, we have added additional control experiments to clearly separate the rectification and fluorescence effects arising from nanofluidic ion transport. We have also expanded the analysis and provided

additional mechanistic validation regarding the device geometry and the inferred ionic conductivity. Detailed point-to-point responses are provided below.

3.1 "Nanofluidic device" characterization across geometries needs stronger support

For open-volume and gap configurations, the effective transport geometry is not well defined, and an effective L/A is chosen so that the inferred conductivity at 0.1 M matches the bulk electrolyte. This assumption may be reasonable as an internal normalization, but it makes absolute conductivity values (and any inferred low-concentration plateau magnitude/'equivalent nanochannel size') dependent on the chosen effective geometry rather than directly constrained by measured dimensions/porosity.

Recommended revisions:

Present raw conductance (G) versus concentration (and representative I–V curves) prior to conversion to conductivity, then show how inferred conductivity depends on the assumed L/A.

Provide a sensitivity analysis showing how plateau magnitude and any extracted characteristic length scale vary over physically plausible L/A bounds (constrained by device dimensions and imaging-based estimates).

Provide an independent constraint on effective cross-section/porosity (image-based estimate, mass/volume plus porosity, or bounding calculations).

Clearly distinguish conclusions based on qualitative trends (robust) versus those relying on absolute conductivity/model fits (model-dependent).

Response: We thank the reviewer for this important comment and the valuable suggestions. To strengthen the characterization of the nanofluidic devices across different geometries, we have **added the raw I–V curves from which the conductance (*G*) values are derived (Supplementary Figure 20–22),** allowing the experimental observations to be presented prior to their conversion into conductivity. We have **provided image-based estimates and bounding calculations** to better constrain the effective cross-section of the transport pathways (Supplementary Note 3), and **performed a sensitivity analysis** to evaluate how the inferred conductivity and the low-concentration plateau depend on the assumed L/A values (Supplementary Note 4). Finally, we have **clarified in the revised manuscript** which conclusions are based on

qualitative trends in conductance (independent of the assumed *L*/*A*) and which analyses rely on absolute conductivity values derived from the assumed *L*/*A*.

Regarding "*Present raw conductance (G) versus concentration (and representative I–V curves) prior to conversion to conductivity*"

We thank the reviewer for this suggestion. The representative I–V curves have been added together with the conductance (*G*) data, as shown in Supplementary Figures 8–10 and Supplementary Figures 20–22, corresponding to the microchannel configuration (Figure 3g), gap configuration (Figure 3k), and open volume configuration (Figure 3n). Full raw I–V data from at least four independent measurements are provided in the source data file. And **we have expanded discussion in the revised manuscript:**

"The negative surface charge, synergistic with hyphal ion pathways, contributes to surface-mediated K^+ transport in the mycelial scaffold and confers a common multi-species ion regulation capability (Figure 2e and Supplementary Figures 8–10), a typical nanofluidic characteristic where high ion conductivity is observed even at low ion concentrations (< 1×10^{-3} mol L^{-1})." (page 7 line 159)

"The I–V curves are used to derive the conductance (Supplementary Figure 20), which is further converted to ionic conductivity using the length and diameter of the microchannel." (page 10 line 239)

"The ion conductance of the *G. sessile* network was derived from the I–V measurements (Supplementary Figure 21) and then converted to ion conductivity using estimated geometric parameters (see Methods and Supplementary Note 3)." (page 11 line 272)

"The nonlinear conductance derived from the I–V measurements (Supplementary Figure 22), together with the ion conductivity of the *P. ostreatus* hyphal network (Figure 3n), further demonstrate the universal nanofluidic ion regulation capability of mycelium." (page 11 line 283)

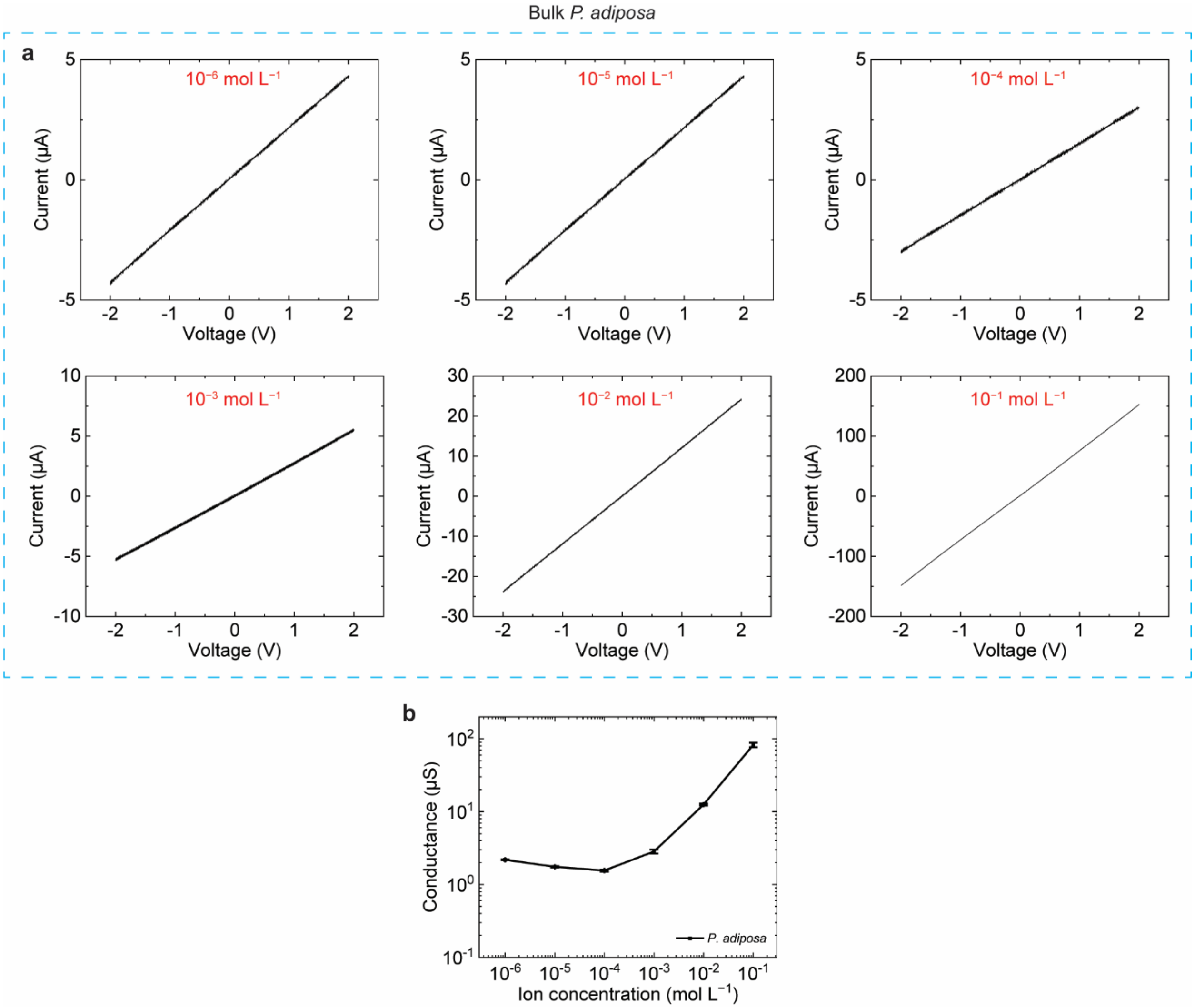

Supplementary Figure 8. I–V characterization of the bulk *P. adiposa* mycelium. (a) Representative I–V curves. (b) Conductance derived from the I–V curves. Full raw data are provided in the source data file.

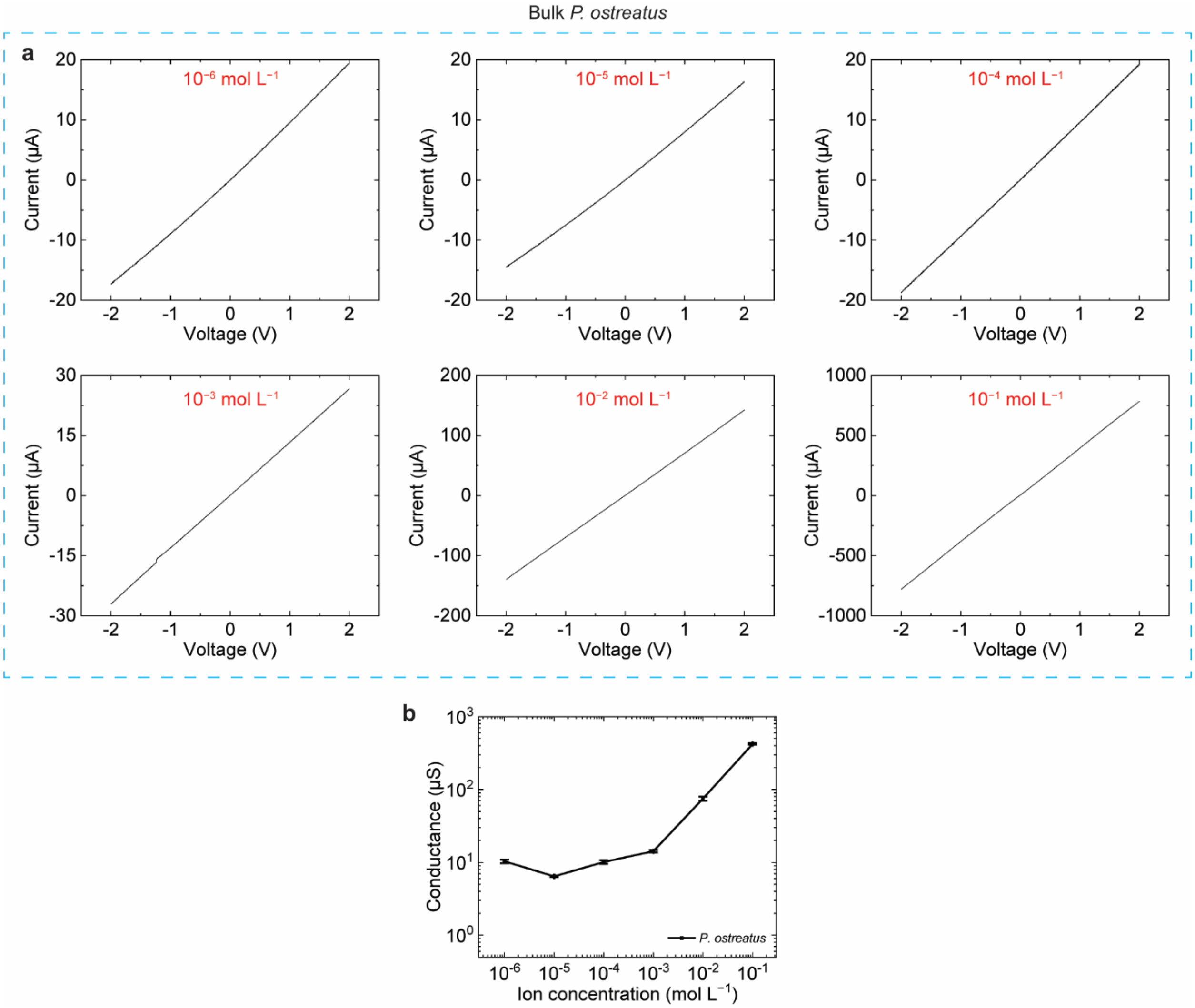

Supplementary Figure 9. I–V characterization of the bulk *P. ostreatus* mycelium. (a) Representative I–V curves. (b) Conductance derived from the I–V curves. Full raw data are provided in the source data file.

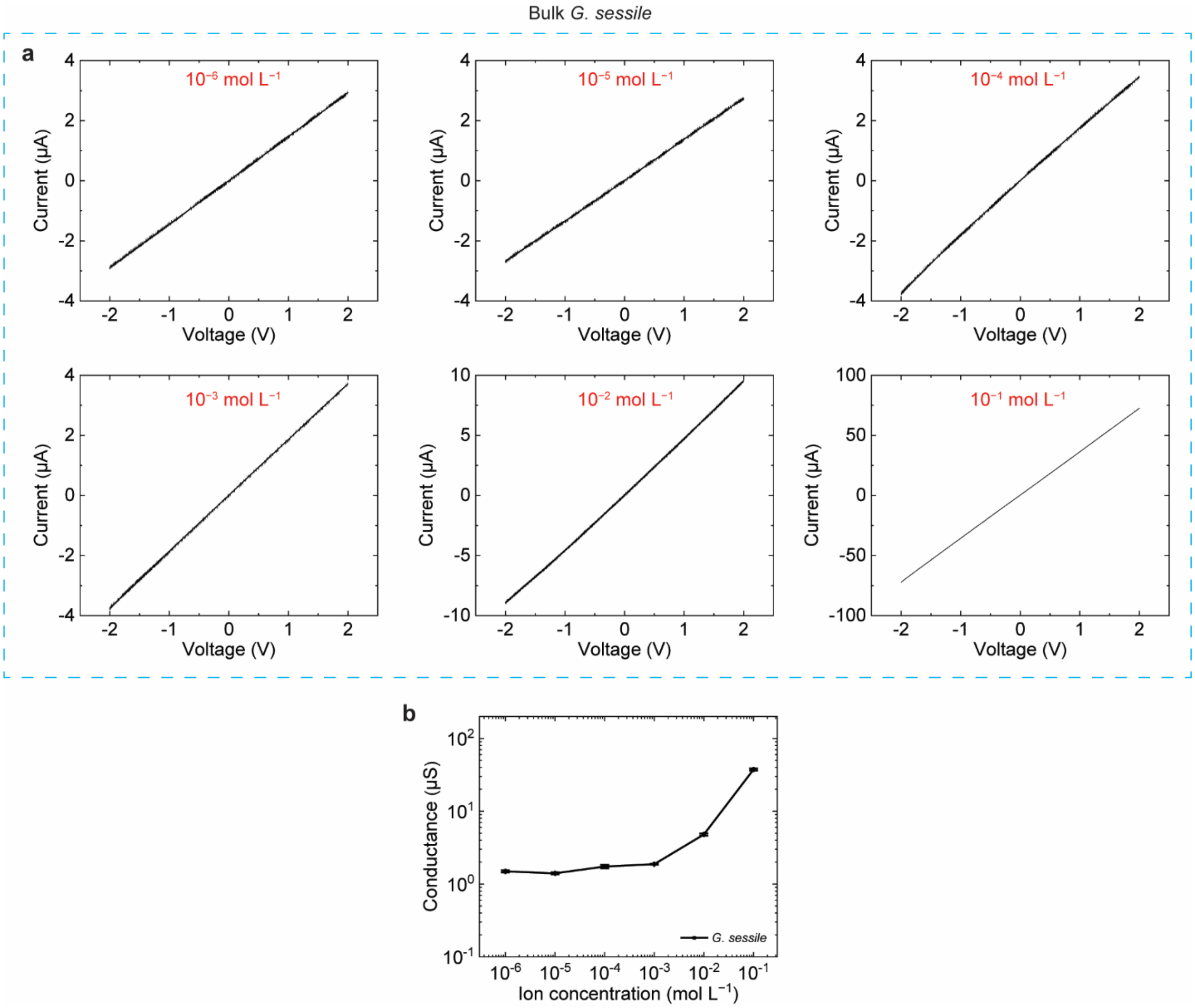

Supplementary Figure 10. I–V characterization of the bulk *G. sessile* mycelium. (a) Representative I–V curves. (b) Conductance derived from the I–V curves. Full raw data are provided in the source data file.

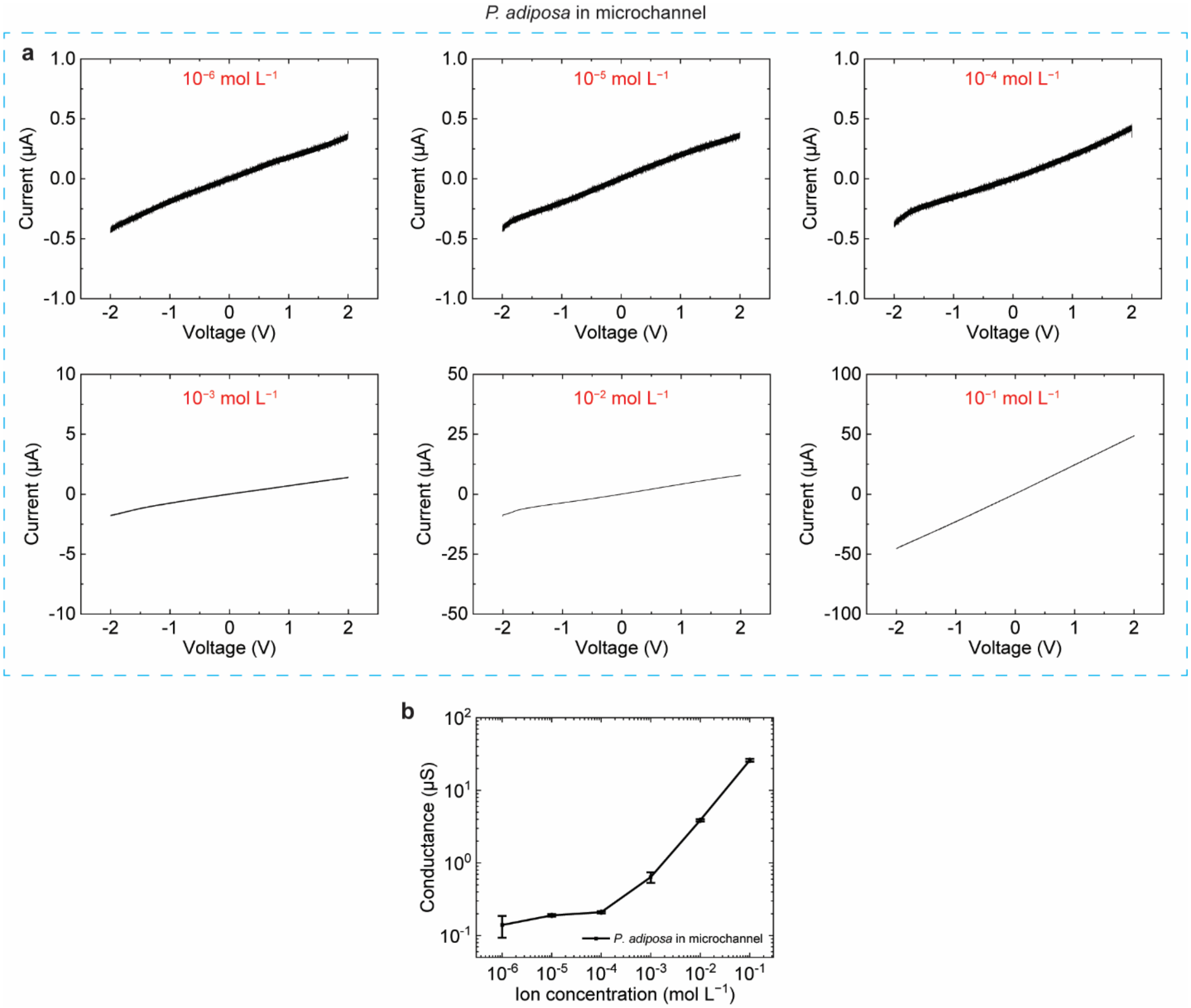

Supplementary Figure 20. I–V characterization of the *P. adiposa* hyphal network in the microchannel, clearly demonstrating nonlinearity due to nanofluidic properties. (a) Representative I–V curves. (b) Conductance derived from the I–V curves. Full raw data are provided in the source data file. The I–V curves are symmetric under forward and reverse bias, indicating that the measured conductivity is independent of the mycelial growth direction within the microchannel.

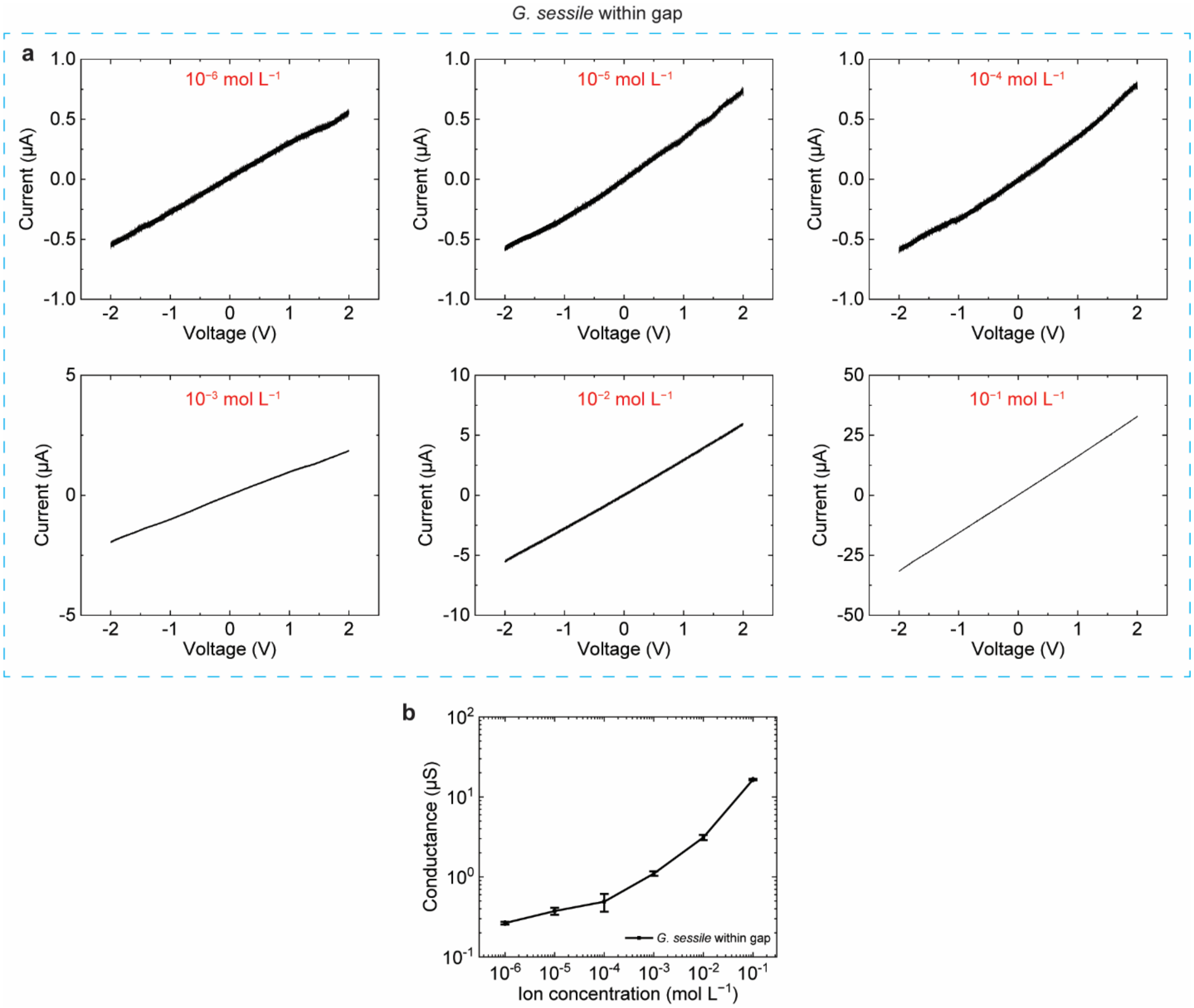

Supplementary Figure 21. I–V characterization of the *G. sessile* hyphal network within the gap between the glass pieces, clearly demonstrating nonlinearity due to nanofluidic properties. (a) Representative I–V curves. (b) Conductance derived from the I–V curves. Full raw data are provided in the source data file.

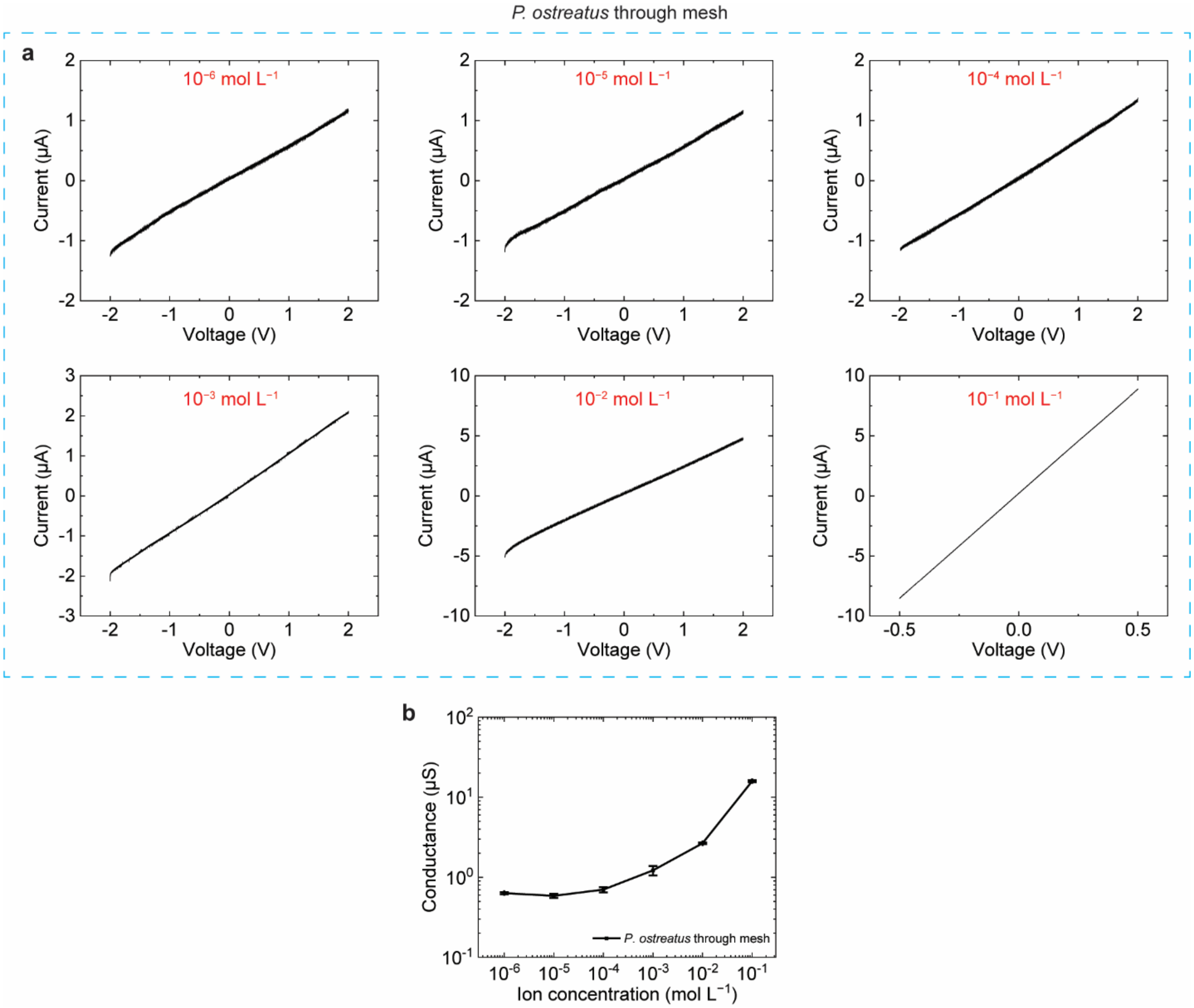

Supplementary Figure 22. I–V characterization of the *P. ostreatus* hyphal network through the metal mesh, clearly demonstrating nonlinearity due to nanofluidic properties. (a) Representative I–V curves. (b) Conductance derived from the I–V curves. Full raw data are provided in the source data file.

Regarding "*Provide an independent constraint on effective cross-section/porosity (image-based estimate, mass/volume plus porosity, or bounding calculations)*"

We thank the reviewer for this suggestion, and we agree that an independent constraint on the geometrical parameter is important. The assumed L/A values were 800 cm^{-1} and 900 cm^{-1} for the gap configuration (Figure 3i) and the open-volume configuration (Figure 3l), respectively. Here,

we **provide estimated ranges for the *L*/*A* bounds** based on image analysis (326–1270 cm^{-1} for gap configuration and 446–1420 cm^{-1} for open-volume configuration), as detailed in Supplementary Note 3 and **expanded discussion in the revised manuscript**.

"The ion conductance of the *G. sessile* network was derived from the I–V measurements (Supplementary Figure 21) and then converted to ion conductivity using estimated geometric parameters (see Methods and Supplementary Note 3)." (page 11 line 272)

"The estimation of the geometric parameters of the nanofluidic pathways and the corresponding sensitivity analysis are provided in Supplementary Note 3–4." (page 12 line 288)

"For mycelium grown through confined gaps and open volumes, the geometric parameters (length and area) of the nanofluidic pathways are not well-defined or directly measurable, so a constant value of L/A is assumed such that the sample conductivity at 0.1 mol L^{-1} matches the corresponding bulk value (800 cm^{-1} for Figure 3i and 900 cm^{-1} for Figure 3l). The assumed values fall within the estimated L/A range: 326–1270 cm^{-1} for Figure 3i and 446–1420 cm^{-1} for Figure 3l (Supplementary Note 3)." (page 22 line 548)

"**Supplementary Note 3.** Estimate for L/A values

In Figure 3i and Figure 3l, mycelium grows through confined gaps and open volumes, where the geometric parameters (length L and area A) of the nanofluidic pathways are not well-defined or directly measurable. This makes it difficult to derive exact ion conductivities. Therefore, an estimate of L/A is provided based on geometric considerations and literature-reported properties of mycelium networks.

For the gap configuration (Figure 3i), the length of the nanofluidic pathway corresponds to the width of the glass slide (L = 2.54 cm). The total cross-sectional area within the 0.3 mm gap is A_{total} = 0.3 mm × 1.3 cm = 0.039 cm^2. However, mycelium forms a sparse filamentous network and does not occupy the entire cross-section. Previous studies4,5 report bulk densities of mycelium networks on the order of 50–200 kg m^{-3}, while individual hyphae—containing 80–90% water—have densities close to that of water (1000–1100 kg m^{-3}). This suggests that the mycelium network has a filling ratio f around 5–20%. Based on this estimate, the effective conductive cross-sectional area is $A_{eff} = A_{total} \times f$ = 0.002–0.0078 cm^2. Consequently, the corresponding L/A range is 326–1270 cm^{-1}. The nominal value used in the conductivity calculation, 800 cm^{-1}, falls within this physically reasonable range.

For the open volume configuration (Figure 3l), the length of the nanofluidic pathway corresponds to the tilted distance between the two agar tanks. The horizontal separation between the two tanks is 0.5 cm, and the hyphae grow at a titled angle ~30°–45°, giving a conductive pathway length L = 0.58–0.71 cm. To estimate the effective cross-sectional area, we count the number of mesh grids N_{grid} through which the hyphae pass: N_{grid} ~ 1500–1800. Each grid contains hyphae number N_{hyphae} = ~5–10, with a hyphae diameter ~3 μm as estimated from the SEM image (Figure 3m). Based on this estimate, the effective conductive cross-sectional area is $A_{eff} = N_{grid} \times N_{hyphae} \times \pi/4 \times (3\ \mu m)^2$ = 0.0005–0.0013 cm^2. Consequently, the corresponding L/A range is 446–1420 cm^{-1}. The nominal value used in the conductivity calculation, 900 cm^{-1}, falls within this physically reasonable range." (Supplementary Note 3)

Regarding "*how inferred conductivity depends on the assumed L/A*" and "*Provide a sensitivity analysis showing how plateau magnitude and any extracted characteristic length scale vary over physically plausible L/A bounds (constrained by device dimensions and imaging-based estimates)*"

We thank the reviewer for this comment and agree that a sensitivity analysis is important given the uncertainty in the geometrical parameters. We have **provided the sensitivity analysis** for the inferred conductivity and the extracted characteristic nanochannel size based on the estimated L/A range as in Supplementary Note 4, and **expanded discussion in the revised manuscript**.

"The sensitivity of the inferred nanochannel size to the assumed geometric parameters is further discussed in Supplementary Note 4." (page 11 line 277)

"The estimation of the geometric parameters of the nanofluidic pathways and the corresponding sensitivity analysis are provided in Supplementary Note 3–4." (page 12 line 288)

"In addition, a sensitivity analysis of the L/A values on the inferred conductivity and equivalent nanochannel size is provided (Supplementary Note 4)." (page 23 line 553)

"**Supplementary Note 4.** Sensitivity based on L/A values

The ion conductivity κ was calculated from $\kappa = \frac{GL}{A}$. Therefore, for a given measured conductance G, the inferred conductivity κ (including the plateau magnitude) increases linearly

with the assumed L/A value. Meanwhile, the extract equivalent nanochannel size based on the relation $\kappa = q(\mu_+ + \mu_-)CN_\mathrm{A} + 2\sigma_\mathrm{s}\mu_+ / d$ changes accrodingly.

To evaluate this sensitivity, we provide results calculated using multiple L/A values within the estimated bounds (Supplementary Figure 1). For the gap configuration, data are provided for L/A = 400 cm^{-1}, 800 cm^{-1} (nominal), and 1200 cm^{-1} within the estimated range of 326–1270 cm^{-1}. For the open volume configuration, data are provided for L/A = 500 cm^{-1}, 900 cm^{-1} (nominal), and 1400 cm^{-1} within the estimated range of 446–1420 cm^{-1}. The resulting equivalent nanochannel sizes remain within the same order of magnitude across the estimated geometric range." (Supplementary Note 4)

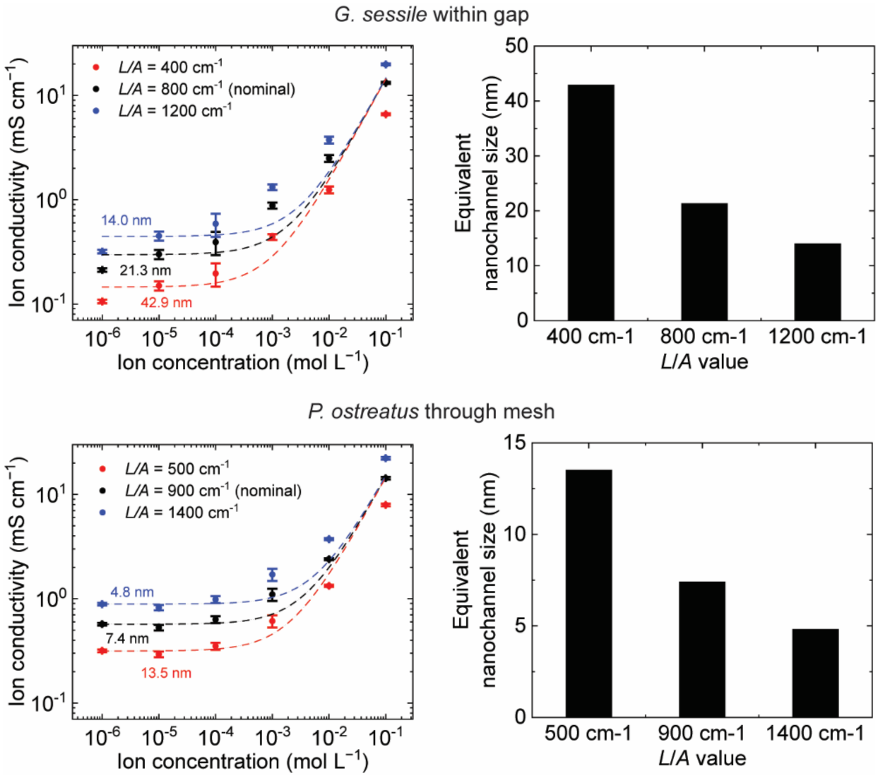

Supplementary Figure 1. Sensitivity analysis based on estimated range of the geometric parameter L/A. The ion conductivity varies linearly with the L/A value, while the equivalent nanochannel size is inversely proportional to the L/A value as indicated by Equation S2. However, the results remain within the same order of magnitude across the estimated geometric range.

Regarding "*Clearly distinguish conclusions based on qualitative trends (robust) versus those relying on absolute conductivity/model fits (model-dependent)*"

We thank the reviewer for this suggestion, and we agree that a clear distinguishment is important for the readers. We have clearly **distinguished the conclusions** that are independent of and dependent on the geometric assumptions and model fitting. **We expand discussion in the revised manuscript**: "As a result, qualitative conclusions based on conductance trends are robust and independent of geometric assumptions, whereas quantitative results (ion conductivity and inferred nanochannel size) are model-dependent and rely on the assumed L/A values. In contrast, the microchannel configuration does not require this L/A estimation because the channel length and cross-sectional area can be directly measured." (page 23 line 554)

3.2 Rectification is modest and mechanism is not fully isolated

Rectification ratios up to ~1.5 under 1000-fold concentration asymmetry are modest, raising the possibility of contributions from access resistance, small geometric asymmetries, and/or concentration polarization rather than strong intrinsic permselectivity.

Recommended revisions:

Include "no mycelium" and surface-charge-reduced/neutralized controls to test whether rectification decreases as expected if surface charge is the dominant mechanism.

Report rectification ratio versus voltage and versus ionic strength and/or pH to link the effect to surface charge.

Define rectification calculation details (voltage points, polarity convention, averaging, sample size) and show reproducibility across devices.

Response: We thank the reviewer for the valuable suggestions. To address these concerns, we have performed additional analyses and experiments in the revised manuscript. Specifically, we have **(i) included control experiments** without mycelium to examine whether rectification decreases in the absence of the mycelial network, **(ii) reported rectification ratios as a function of voltage, ionic strength, and pH** to examine their relationship with surface-charge-regulated transport, and **(iii) clarified the rectification calculation details** (voltage points, polarity convention, averaging

procedure, and sample size) and provided reproducibility data across multiple devices. These results and discussions have been added in the revised manuscript as detailed below.

Regarding "*Include 'no mycelium' control*"

We thank the reviewer for this comment. We supplemented the "no mycelium" control test and it shows no clear rectification. We have expanded the discussion in the revised manuscript: "The different bath concentrations create different chemical potentials at the two ends of the device, resulting in diode-like current behavior (Figure 4e), which is absent in the control device without mycelium (Supplementary Figure 25)." (page 14 line 332)

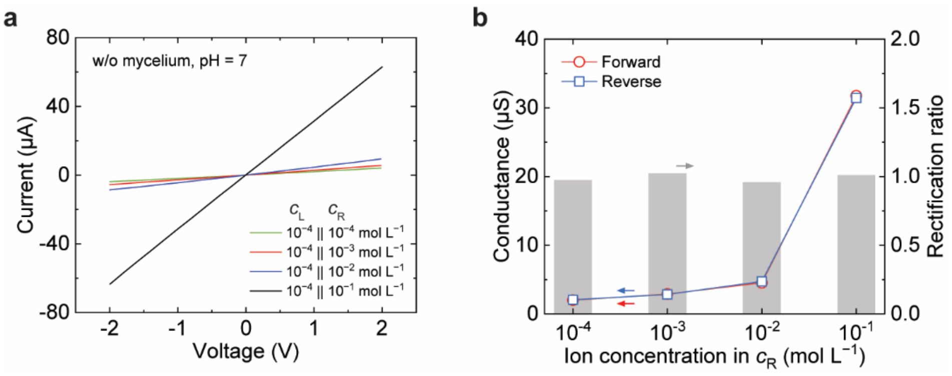

Supplementary Figure 25. Current–voltage measurements of the control device without mycelium under asymmetric bath concentrations. (a) Current–voltage response. (b) Corresponding rectification ratio, which remains close to unity, indicating the absence of clear rectification. This behavior is consistent with the lack of surface-charge-regulated ion transport in the absence of mycelial hyphae.

Regarding "*Include surface-charge-reduced/neutralized controls to test whether rectification decreases as expected if surface charge is the dominant mechanism*" and "*Report rectification ratio versus voltage and versus ionic strength and/or pH to link the effect to surface charge*"

We thank the reviewer for these valuable suggestions on linking the rectification behavior to surface charge, a key factor governing nanofluidic ion transport. We have **supplemented additional experiments at different voltages, ion concentrations, and pH values** to link the rectification behavior to surface charge and demonstrate that the rectification originates from the

concentration gradient across the device. **We have expanded discussion in the revised manuscript** as below.

"The forward and reverse conductances exhibit a modest rectification ratio of 1.46±0.06 (Figure 4f and Supplementary Figure 26), which is consistent with that observed in bulk mycelium (Supplementary Figure 27)." (page 14 line 337)

"In addition to the concentration asymmetry, the rectification ratio shows a slight increase with applied voltage and pH (Supplementary Figures 29–30), consistent with enhanced asymmetric ion transport under a stronger electric field and increased surface charge within the mycelial scaffold." (page 14 line 349)

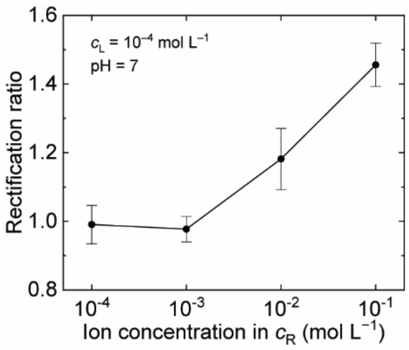

Supplementary Figure 26. Rectification ratio from –3 V to 3 V at different ion concentrations. Error bars indicate the standard deviation across three independent devices fabricated from separately grown batches.

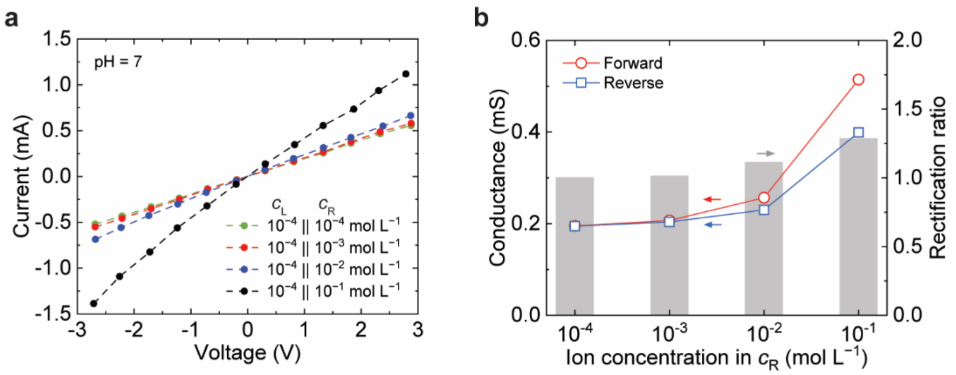

Supplementary Figure 27. Current–voltage measurements of bulk P. adiposa mycelium under asymmetric bath concentrations. (a) Current–voltage response. (b) Corresponding rectification

ratio, reaching up to 1.3. This behavior is consistent with the device measurements shown in Figure 4e–f.

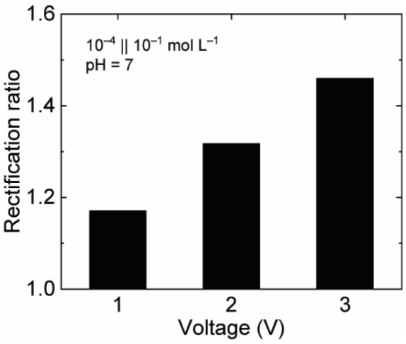

Supplementary Figure 29. Rectification ratio at different applied voltages. The rectification ratio increases modestly from ~1.2 at 1 V to ~1.5 at 3 V, consistent with field-driven enhancement of the asymmetric ion transport established by the concentration gradient and surface charge within the mycelial scaffold.

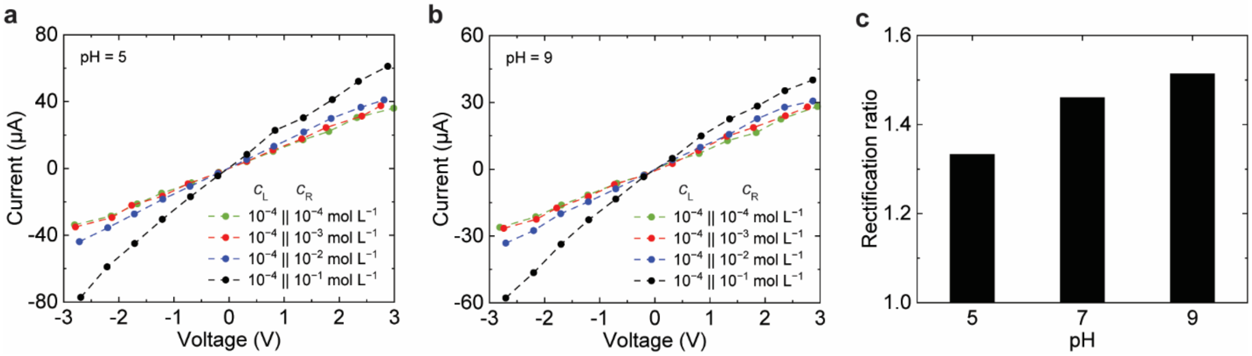

Supplementary Figure 30. Rectification behavior under different pH conditions. (a–b) Current–voltage responses measured at pH = 5 and pH = 9, respectively. (c) Corresponding rectification ratio as a function of pH. A higher pH leads to stronger rectification, consistent with increased negative surface charge of the mycelial network due to deprotonation of surface functional groups, which enhances cation-selective ion transport.

Regarding "*Define rectification calculation details (voltage points, polarity convention, averaging, sample size) and show reproducibility across devices*"

We thank the reviewer for the important comments. We have supplemented reproducibility results of rectification performance and provided the rectification calculation details.

"The forward and reverse conductances exhibit a modest rectification ratio of 1.46±0.06 (Figure 4f and Supplementary Figure 26), which is consistent with that observed in bulk mycelium (Supplementary Figure 27)." (page 14 line 337)

"In the rectification tests, KCl solutions of different concentrations were added to the two ends of the microchannel device. The I–V curves were measured from –3 V to 3 V with a voltage step of 0.5 V. The forward and reverse conductances were extracted as the linear slopes of the I–V curves under negative and positive bias, respectively, and the rectification ratio was defined as the ratio between these two conductances. Each I–V measurement was repeated three times for each device, and the reported values correspond to the averaged results. Error bars represent the standard deviation obtained from measurements on three independent devices fabricated from separately grown batches." (page 23 line 561)

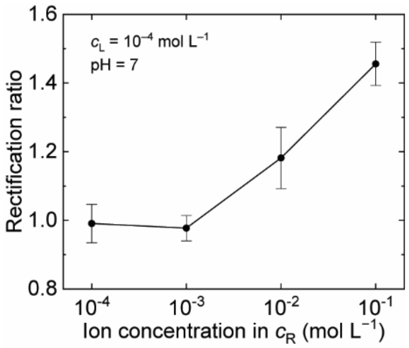

Supplementary Figure 26. Rectification ratio from –3 V to 3 V at different ion concentrations. Error bars indicate the standard deviation across three independent devices fabricated from separately grown batches.

3.3 Optical enrichment claims need controls to distinguish enrichment from adsorption/partitioning

Recommended revisions:

State explicitly (in main Methods or SI) the enhancement metric used in the SI: ROI definition and within-channel minus outside-channel background subtraction, including any normalization.

Add adsorption/retention controls (e.g., time-course washout; inert porous control of similar porosity; or chemically/passivation-treated scaffold) to bound partitioning contributions.

Show polarity switching/time dynamics: if enhancement reverses promptly with polarity and diminishes on washout, that supports enrichment; persistence would suggest adsorption/retention.

Where possible, report analytical performance (SNR, repeatability, and any LOD/LOQ shift), not fold-intensity alone.

Response: We thank the reviewer for the valuable suggestions. To address these concerns, we have added additional analyses and controls in the revised manuscript. Specifically, we have **(i) provided step-by-step procedures for the image processing** used to quantify the fluorescence enhancement, including ROI definition and background subtraction, **(ii) supplemented washing tests and pH-treatment tests** to help distinguish ion enrichment from possible adsorption or partitioning effects, and **(iii) further reported analytical performance metrics** including signal-to-noise ratio (SNR) and repeatability across measurements.

Regarding "*State explicitly (in main Methods or SI) the enhancement metric used in the SI: ROI definition and within-channel minus outside-channel background subtraction, including any normalization*"

We thank the reviewer for these important suggestions that help improve the clarity of the manuscript. The region of interest (ROI) is a line spanning the microchannel from the fluorescence images. We have explicitly stated the ROI definition and included the step-by-step image processing procedures in the revised manuscript: "Fluorescent images were acquired at the center of the microchannel immediately after introducing 1×10^{-6} mol L^{-1} Fluo-4FF solution without any applied voltage bias. Image analysis was performed using ImageJ. The image color was split into RGB channels and converted into grayscale images. The green channel was used for analysis because Fluo-4FF emits green fluorescence when bound to Ca^{2+}. The region of interest (ROI) was defined as a line spanning the microchannel with a width equal to twice the microchannel diameter (Supplementary Figure 38). Intensity profiles extracted from five such lines were averaged to obtain the green channel intensity without further normalization." (page 21 line 508)

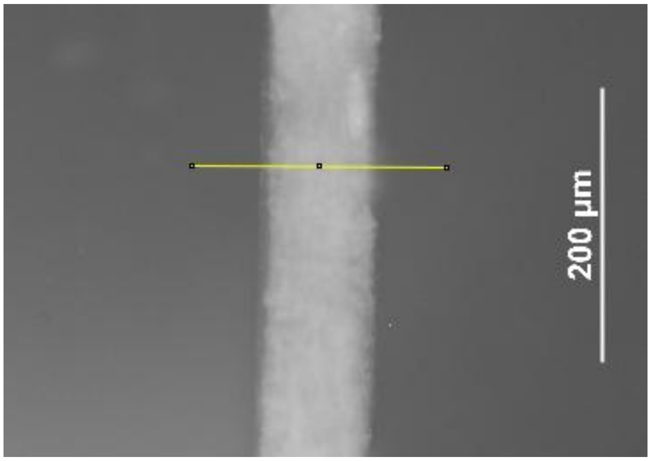

Supplementary Figure 38. Region of interest (ROI) used for intensity extraction in the green-channel grayscale image given by ImageJ.

The enhancement metric used in this study is the background-subtracted net fluorescence intensity, compared between conditions with and without hyphae. The background subtraction (within-channel minus outside-channel intensity) is summarized in Supplementary Table 1 and further clarified in Supplementary Note 5: "The within-microchannel value corresponds to the maximum intensity measured within the microchannel region, while the outside-of-microchannel value corresponds to the average intensity measured in the background region. The enhancement in the background-subtracted net fluorescence intensity ($I_{\text{true fluorescence}}$, with hyphae vs. without hyphae) is used to quantify the sensitivity enhancement as summarized in Supplementary Table 1." (Supplementary Note 5)

Supplementary Table 1. Green channel intensity for four cases in Figure 4j–l

Green color intensity (a.u.)		Within-microchannel	Out-of-microchannel	True fluorescence	Enhancement	Background noise	SNR
10^{-6} mol L^{-1}	w/o hyphae	37.3	35.5	1.8	55×	0.79	2.3
	w/ hyphae	136.6	38.0	98.6		0.99	99.7
10^{-5} mol L^{-1}	w/o hyphae	54.1	50.2	3.9	24×	1.13	3.5

	w/ hyphae	184.0	92.1	91.9		1.16	79.0

Regarding "*Add adsorption/retention controls (e.g., inert porous control of similar porosity) to bound partitioning contributions*"

We thank the reviewer for the suggestion to include control experiments to bound adsorption or partitioning. This is important for establishing nanofluidic ion enrichment as the underlying mechanism in our system. We performed a control experiment using Fluo-4FF without Ca^{2+} to confirm that the fluorescence enhancement originates from Ca^{2+} enrichment. **We have expanded the discussion in the revise manuscript:** "However, the hyphae in the microchannel greatly enhance the fluorescent effect due to their nanofluidic ion enrichment capability (Figure 4j bottom), compared with the control condition containing Fluo-4FF but no Ca^{2+} (Supplementary Figure 32), thus expanding the detection range of Fluo-4FF." (page 16 line 380)

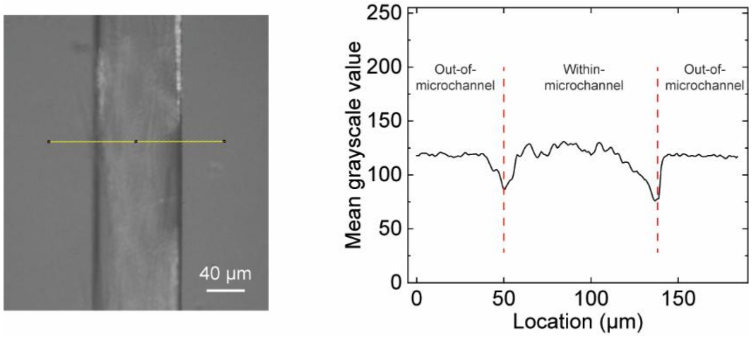

Supplementary Figure 32. Control fluorescence image of the mycelium-integrated microchannel containing the fluorescence indicator Fluo-4FF but no Ca^{2+}. The extracted grayscale image of the green channel shows no clear difference between the microchannel region and the surrounding area, likely because immersion of the mycelium in aqueous solution reduces optical scattering due to refractive index matching. The line profile shows that the grayscale value increases from ~120 out of the microchannel to ~130 within the microchannel, indicating that the scattering signal without fluorescence is ~10 in grayscale value and contributes less than ~10% of the observed fluorescence enhancement.

Regarding "*Add adsorption/retention controls (e.g., chemically/passivation-treated scaffold) to bound partitioning contributions*"

We thank the reviewer for the suggestion to use a chemically treated scaffold to bound adsorption or partitioning. This is important for supporting nanofluidic ion enrichment as the underlying mechanism in our system. We performed fluorescence measurements under different pH conditions to probe the role of surface charge in the observed fluorescence response. The fluorescence signal becomes stronger at higher pH, consistent with an increase in the negative surface charge of the mycelial scaffold. **We have expanded the discussion in the revise manuscript:** "The nanofluidic behavior is further supported by pH-dependent measurements probing the surface charge effect, where a more negative surface charge leads to stronger cation attraction (Supplementary Figure 35)." (page 16 line 387)

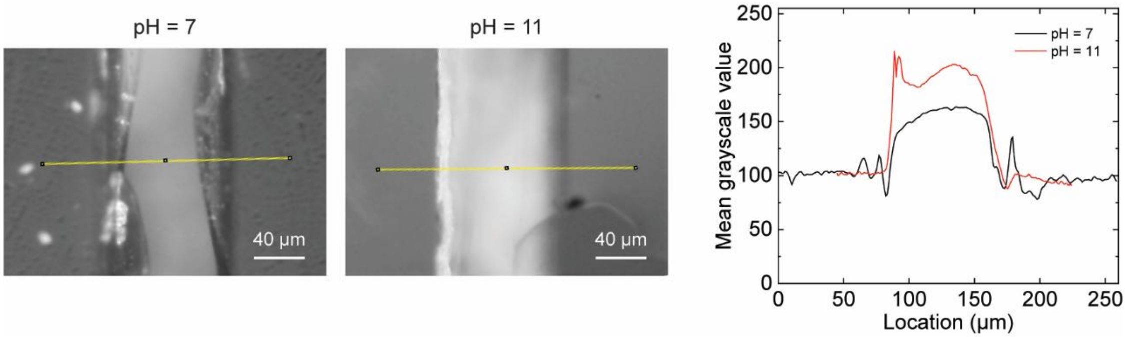

Supplementary Figure 35. Fluorescence images of the mycelium-integrated microchannel under different pH conditions. The mycelial hyphae appear out of focus due to the cylindrical geometry of the microchannel. Increasing pH enhances the negative surface charge of the mycelial scaffold, leading to stronger cation attraction and increased fluorescence intensity, as observed from the grayscale values. This behavior is consistent with surface-charge-regulated ion enrichment rather than partitioning. The pH was adjusted by adding KOH solution, which also introduces additional cations that may interact with the mycelial scaffold.

Regarding "*Add adsorption/retention controls (e.g., time-course washout) to bound partitioning contributions*" and "*Show time dynamics*: *if enhancement diminishes on washout, that supports enrichment; persistence would suggest adsorption/retention*"

We thank the reviewer for the suggestion to perform washout tests to bound adsorption or partitioning. These tests help validate nanofluidic ion enrichment as the underlying mechanism in our system. We performed washout tests to remove Ca^{2+} from the scaffold, and the fluorescence signal shows a significant decrease after rinsing. This result serves as a control to rule out irreversible ion adsorption. **We have expanded discussion in the revised manuscript:** "The enhancement is unlikely to originate from ion adsorption, as the fluorescence diminishes after a washout step (Supplementary Figure 36), indicating reversible ion accumulation." (page 16 line 389)

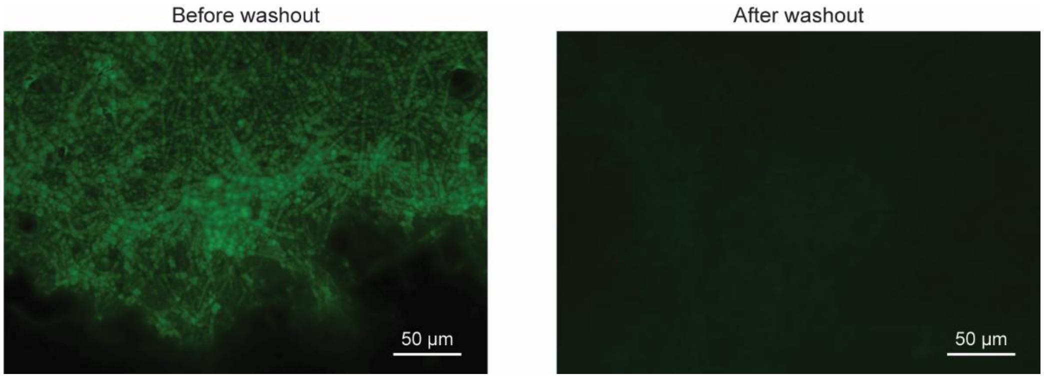

Supplementary Figure 36. Fluorescence image of the Ca^{2+} washout test after rinsing with deionized water for five minutes. As the mycelium integrated in the channel is difficult to rinse due to its embedded structure, bulk *P. adiposa* mycelium was used for the Ca^{2+} washout test. The significantly reduced fluorescence after washout indicates that the fluorescence enhancement in mycelium is unlikely to arise from ion adsorption.

Regarding "*Show polarity switching*: *if enhancement reverses promptly with polarity*"

We thank the reviewer for the suggestion regarding polarity switching, as it may affect the observed fluorescence enhancement. It should be noted that the ion enrichment observed in this work is a passive process occurring without applied voltage bias. Therefore, polarity-switching experiments are not applicable in this system. We have clarified this point in the revised manuscript as follows: "Fluorescent images were acquired at the center of the microchannel immediately after introducing 1×10^{-6} mol L^{-1} Fluo-4FF solution without any applied voltage bias." (page 21 line 508)

Regarding "*Where possible, report analytical performance (SNR, and any LOD/LOQ shift), not fold-intensity alone*"

We thank the reviewer for the value suggestions to explore additional analytical performance metrics beyond the fold-intensity. We have provided further analysis of the signal-to-noise ratio (SNR) and the effective limit of detection (LOD) in Supplementary Note 5 and **expanded the discussion in the revised manuscript.**

"Further analysis shows that the signal-to-noise ratio (SNR) increases from 2.3 to 99.7 and from 3.5 to 79.0 for the two dilute Ca^{2+} solutions, respectively, due to the nanofluidic hyphal network (Supplementary Table 1). Correspondingly, the effective limit of detection (LOD) is improved by approximately one order of magnitude (Supplementary Note 5)." (page 16 line 398)

"Furthermore, the signal-to-noise ratio (SNR) is defined as

$$\mathrm{SNR} = I_{\mathrm{true\ fluorescence}}/\sigma_{\mathrm{background}} \quad \text{(S4)}$$

where $\sigma_{\mathrm{background}}$ is the standard deviation of the background intensity. The presence of the nanofluidic hyphal network increases the SNR from 2.3 to 99.7 and from 3.5 to 79.0 for the two dilute Ca^{2+} solutions, respectively. At 10^{-5} mol L^{-1} Ca^{2+} concentration, the SNR is only slightly above 3 without hyphae, whereas the SNR is significantly improved in the presence of hyphae even at a lower concentration of 10^{-6} mol L^{-1}. This result indicates that the mycelial scaffold improves the effective limit of detection (LOD) by approximately one order of magnitude." (Supplementary Note 5)

Regarding "*Where possible, report analytical performance (repeatability), not fold-intensity alone*"

We thank the reviewer for this comment and agree that repeatability is important for the proposed approach. We extracted five line scans from different samples under identical imaging conditions and averaged them to obtain the line profile shown in Figure 4k. Standard deviation error bars have also been added to Figure 4l. **The corresponding discussion has been expanded in the revised manuscript.**

"The acquired fluorescent images were processed to extract the green color channel that Fluo-4FF emits (see Methods), and the radial profiles clearly demonstrate that the presence of hyphae

remarkably enhances the green channel intensity (Figure 4k), with enhancements of 55 ± 3-fold and 24 ± 2-fold for the two low concentrations (Figure 4l).” (page 16 line 395)

“The region of interest (ROI) was defined as a line spanning the microchannel with a width equal to twice the microchannel diameter (Supplementary Figure 38). Intensity profiles extracted from five such lines were averaged to obtain the green channel intensity without further normalization.” (page 21 line 512)

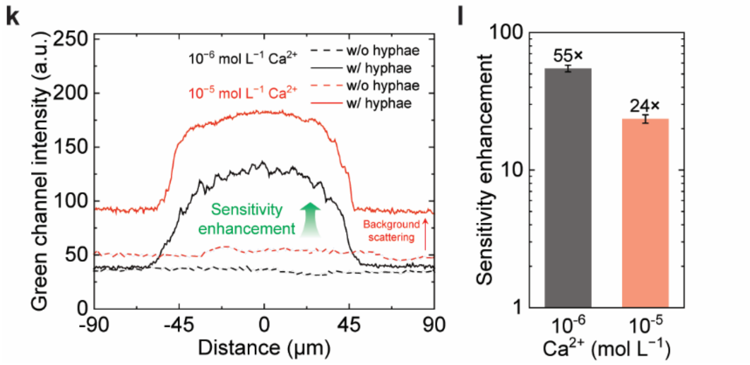

Figure 4. Performance and visualization of self-grown nanofluidic devices. (k) Radial profile of green channel intensity for low-concentration Ca^{2+} detection (10^{-6} and 10^{-5} mol L^{-1}), extracted from processed images, indicating that the integrated hyphae clearly enhance the green signal. (l) Sensitivity enhancement derived from the green channel intensity (Supplementary Note 5). Error bars represent the standard deviation of measurements from three devices fabricated from independently grown patches.

4. Flaws in analysis, interpretation, and conclusions

None of the issues appear fatal, but they require revision for soundness and to prevent overinterpretation:

• Geometry normalization (gap/volume) makes some quantitative conclusions underdetermined without sensitivity bounds.

• “Diode” language should be moderated or more strongly substantiated given rectification magnitude.

• *Some interpretations would benefit from explicit discussion of alternative explanations (access resistance, nonspecific adsorption, scattering artifacts) and how the presented controls address them.*

Response: We thank the reviewer for summarizing these concerns. Specifically, we have (i) provided geometric parameter bound calculations and sensitivity analysis in response to Comment 3.1, and (ii) revised the wording related to diode behavior and provided additional data representation in response to Comment 3.2.

Here, **we have expanded explicit discussion of alternative explanations,** including access resistance, nonspecific adsorption, and scattering artifacts, and clarified how the control experiments address these possibilities.

"While access resistance may contribute to the overall device resistance, its magnitude is expected to be much smaller than the resistance of the extended mycelial network spanning the millimeter-scale microchannel. This interpretation is further supported by the observation that the device performance is consistent with that of bulk mycelium materials (Figure 2e)." (page 10 line 243)

"However, the hyphae in the microchannel greatly enhance the fluorescent effect due to their nanofluidic ion enrichment capability (Figure 4j bottom), compared with the control condition containing Fluo-4FF but no Ca^{2+} (Supplementary Figure 32), thus expanding the detection range of Fluo-4FF." (page 16 line 380)

"The nanofluidic behavior is further supported by pH-dependent measurements probing the surface charge effect, where a more negative surface charge leads to stronger cation attraction (Supplementary Figure 35)." (page 16 line 387)

"The enhancement is unlikely to originate from ion adsorption, as the fluorescence diminishes after a washout step (Supplementary Figure 36), indicating reversible ion accumulation." (page 16 line 389)

"Notably, the background light intensity in the region outside the microchannel may increase due to optical scattering from the strong fluorescence within the microchannel. To address this issue, background subtraction was performed to extract the true fluorescence signal (Supplementary Note 5)." (page 16 line 392)

"The line profile shows that the grayscale value increases from ~120 out of the microchannel to ~130 within the microchannel, indicating that the scattering signal without fluorescence is ~10 in grayscale value and contributes less than ~10% of the observed fluorescence enhancement." (Supplementary Figure 32 caption)

"Increasing pH enhances the negative surface charge of the mycelial scaffold, leading to stronger cation attraction and increased fluorescence intensity, as observed from the grayscale values. This behavior is consistent with surface-charge-regulated ion enrichment rather than partitioning." (Supplementary Figure 35 caption)

5. Methodology and expected standards

Overall the methods appear reasonable for a proof-of-concept, and the device concept is well-motivated. The most important soundness concerns are (i) quantitative interpretation when geometry is uncertain, (ii) replication and reporting clarity for key outcomes, and (iii) controls that separate enrichment from adsorption and rectification from generic asymmetries.

Response: We thank the reviewer for summarizing these concerns. Specifically, we have (i) addressed the geometry uncertainty by providing L/A bound calculations and sensitivity analysis in response to Comment 3.1, (ii) clarified the experimental procedures and reporting details for the key measurements in response to Comments 3.1–3.3, and (iii) supplemented control experiments to distinguish enrichment from adsorption and rectification from generic asymmetries. These controls include washout tests, zero-Ca^{2+} tests, and pH-treated tests for ion enrichment, as well as no-mycelium controls, pH-dependent tests, and voltage-dependent measurements for rectification, as detailed in our responses to Comments 3.3 and 3.2, respectively.

6. Reproducibility and methods detail

Several methods are described clearly at a high level, including the measurement setup and analysis tools. However, the manuscript should tighten reporting in the following areas:

• Replicates: The methods note error bars from three independent devices. Each key figure should state n, what constitutes an independent device (separate inoculation/growth batch vs same batch), and whether error bars are SD or SEM.

• *Conductance extraction: Define how conductance is obtained from I–V curves (fit range, linearity criteria).*

• *Image quantification: Provide step-by-step image processing (exact ImageJ steps, ROIs, background correction) in Methods or SI.*

• *Geometry-dependent conductivity: Provide bounds/estimates for effective dimensions for each configuration; if not possible, provide a sensitivity analysis (as above).*

Response: We thank the reviewer for raising these concerns. We have addressed them one-by-one.

Regarding "*Several methods are described clearly at a high level, including the measurement setup and analysis tools*"

We thank the reviewer for this comment, and **we have added additional details to the Methods section regarding** fluorescence image processing, ion conductivity measurements, and rectification tests, as described below.

"Fluorescent images were acquired using an Olympus BX-51 microscope equipped with a DP-71 camera and a U-MWB2 filter cube (blue excitation/green emission), with an exposure time of 5 s and ISO200 sensitivity. The mycelium-integrated microchannel was washed first to remove residual ions and then pre-soaked with Ca^{2+} solution at a specific concentration for one hour. Fluorescent images were acquired at the center of the microchannel immediately after introducing 1×10^{-6} mol L^{-1} Fluo-4FF solution without any applied voltage bias. Image analysis was performed using ImageJ. The image color was split into RGB channels and converted into grayscale images. The green channel was used for analysis because Fluo-4FF emits green fluorescence when bound to Ca^{2+}. The region of interest (ROI) was defined as a line spanning the microchannel with a width equal to twice the microchannel diameter (Supplementary Figure 38). Intensity profiles extracted from five such lines were averaged to obtain the green channel intensity without further normalization." (page 21 line 504)

"For mycelium grown through confined gaps and open volumes, the geometric parameters (length and area) of the nanofluidic pathways are not well-defined or directly measurable, so a constant value of L/A is assumed such that the sample conductivity at 0.1 mol L^{-1} matches the corresponding bulk value (800 cm^{-1} for Figure 3i and 900 cm^{-1} for Figure 3l). The assumed values fall within the estimated L/A range: 326–1270 cm^{-1} for Figure 3i and 446–1420 cm^{-1} for Figure 3l

(Supplementary Note 3). In addition, a sensitivity analysis of the *L*/*A* values on the inferred conductivity and equivalent nanochannel size is provided (Supplementary Note 4). As a result, qualitative conclusions based on conductance trends are robust and independent of geometric assumptions, whereas quantitative results (ion conductivity and inferred nanochannel size) are model-dependent and rely on the assumed *L*/*A* values. In contrast, the microchannel configuration does not require this *L*/*A* estimation because the channel length and cross-sectional area can be directly measured." (page 22 line 548)

"In the rectification tests, KCl solutions of different concentrations were added to the two ends of the microchannel device. The I–V curves were measured from –3 V to 3 V with a voltage step of 0.5 V. The forward and reverse conductances were extracted as the linear slopes of the I–V curves under negative and positive bias, respectively, and the rectification ratio was defined as the ratio between these two conductances. Each I–V measurement was repeated three times for each device, and the reported values correspond to the averaged results. Error bars represent the standard deviation obtained from measurements on three independent devices fabricated from separately grown batches." (page 23 line 561)

Regarding "*Replicates: The methods note error bars from three independent devices. Each key figure should state n, what constitutes an independent device (separate inoculation/growth batch vs same batch), and whether error bars are SD or SEM.*"

We thank the reviewer for this comment, and we agree that reproducibility is important for the proposed approach. **We have provided information on error bars and experimental replicates** for all relevant figures and key measurements, as described below.

"Error bars represent the standard deviation of five samples from independently grown patches." (Figure 2 caption)

"All error bars represent the standard deviation of measurements from at least three devices fabricated from independently grown patches." (Figure 3 caption)

"Error bars represent the standard deviation of measurements from three devices fabricated from independently grown patches." (Figure 4 caption)

"Error bars represent the standard deviation calculated from measurements of at least three devices from independently grown patches." (page 22 line 542)

"Each I–V measurement was repeated three times for each device, and the reported values correspond to the averaged results. Error bars represent the standard deviation obtained from measurements on three independent devices fabricated from separately grown batches." (page 23 line 565)

Regarding "*Conductance extraction: Define how conductance is obtained from I–V curves (fit range, linearity criteria)*"

We thank the reviewer for this comment. **We have clarified how conductance is extracted from the I–V curves,** including the fitting range and procedure.

"The conductance was extracted from the linear slope of the I–V curves from –2 V to 2 V." (page 22 line 541)

"The I–V curves were measured from –3 V to 3 V with a voltage step of 0.5 V. The forward and reverse conductances were extracted as the linear slopes of the I–V curves under negative and positive bias, respectively, and the rectification ratio was defined as the ratio between these two conductances." (page 23 line 562)

Regarding "*Image quantification: Provide step-by-step image processing (exact ImageJ steps, ROIs, background correction) in Methods or SI*"

We thank the reviewer for summarizing the missing details in the image quantification. We have provided the step-by-step image processing procedure, including the ImageJ workflow (RGB channel splitting and line-scan analysis), ROI definition, and background subtraction, as detailed in our response to Comment 3.3.

Regarding "*Geometry-dependent conductivity: Provide bounds/estimates for effective dimensions for each configuration; if not possible, provide a sensitivity analysis (as above).*"

We thank the reviewer for summarizing the missing details in the geometry-dependent conductivity. We have provided bound calculations for the geometric parameter L/A, along with the corresponding sensitivity analysis, as detailed in our response to Comment 3.1.

7. Transparency: data availability

The manuscript states that data are available upon reasonable request.

For Nat Comms, the authors should strongly consider depositing raw/processed datasets and analysis scripts in a public repository, or clearly justify any constraints. At minimum, include the key raw I–V datasets and image quantification outputs.

Response: We have provided a source data file containing the datasets associated with the figures, including conductivity, conductance, and image quantification outputs. The raw I–V datasets have also been included in the source data file.

8. Minor points and presentation

• Consider adding a short schematic summarizing the three device geometries and the hypothesized transport mechanism (surface-charge dominated regime, concentration polarization) to guide readers.

Response: We thank the reviewer for this helpful suggestion. **We have updated Figure 3** to include schematic illustrations of the three device geometries together with the hypothesized ion transport mechanism, highlighting surface-charge-regulated transport.

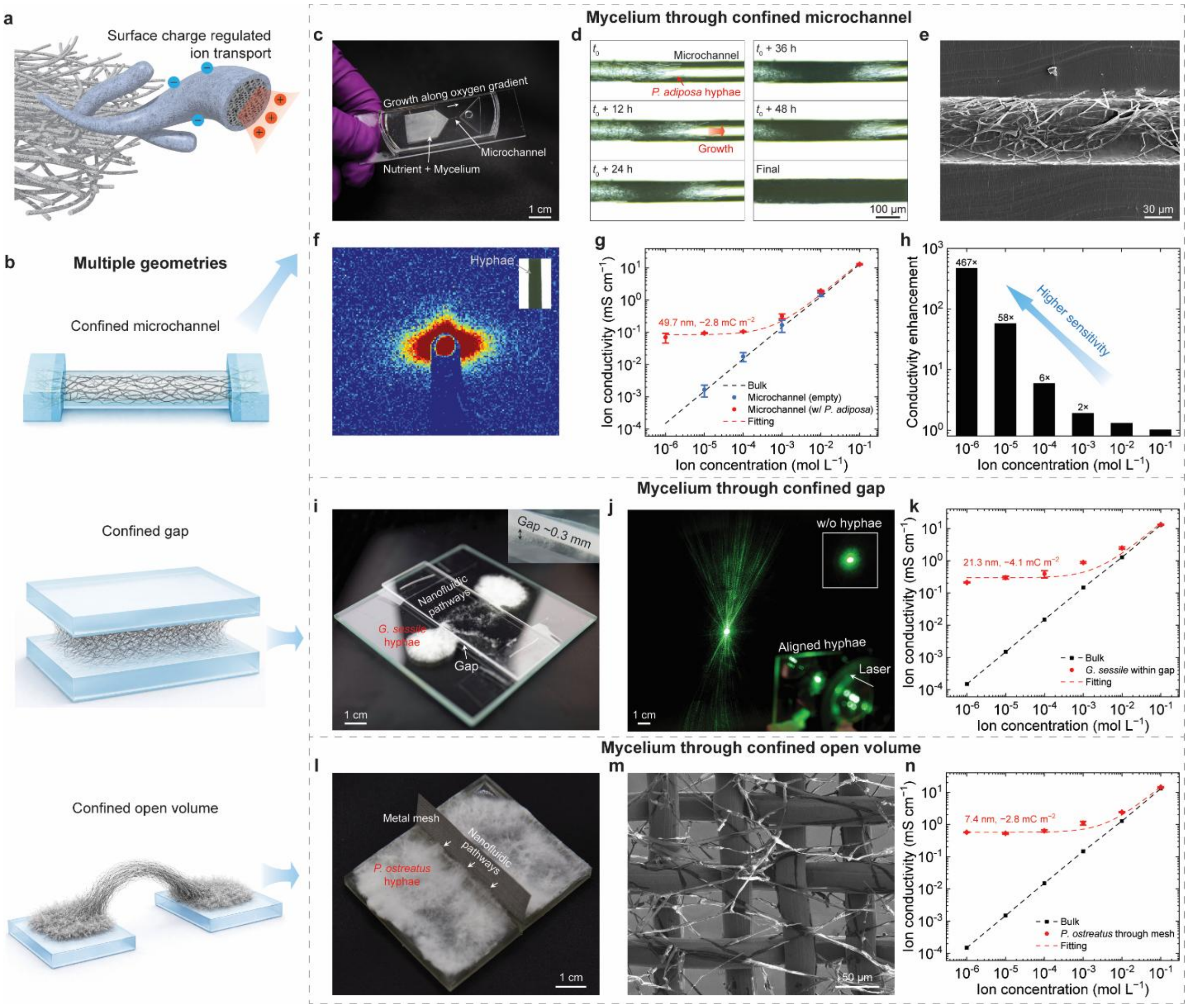

Figure 3. Self-grown mycelium-enabled nanofluidics. (a) Schematic of surface charge regulated ion transport in mycelium. (b) Demonstration of guided mycelium growth forming nanofluidic pathways across multiple device geometries.

Recommendation

Major revision. The concept is novel and qualitative demonstrations are promising, however the manuscript needs strengthened quantification (especially where geometry is uncertain), clearer mechanistic controls,and more explicit replicate/statistical reporting. These appear addressable with additional analysis/controls and clearer reporting, so I recommend major revision rather than rejection.

Response: We thank the reviewer for recognizing the novelty and the promising qualitative demonstrations of our work, as well as for the valuable suggestions for improving the manuscript. In response to the reviewer's comments, we have strengthened the quantification, added additional mechanistic controls, and improved the reporting of replicates and statistical analyses. These revisions address the reviewer's concerns and are detailed in the point-by-point responses above:

- Response to Comment 3.1: Geometry uncertainty and sensitivity analysis
- Response to Comment 3.2: mechanistic controls for rectification behavior
- Response to Comment 3.3: mechanistic controls for fluorescence characterization
- Response to Comment 4–6: clarification, method details, and replicate/statistical reporting

Reviewer #2 (Remarks to the Author):

In this work, the authors demonstrate a method for integrating mycelium into microstructures to form nanofluidic networks. The overall strategy is straightforward, and the guided growth of mycelium is impressive. However, a couple of issues should be addressed before publication. Most importantly, the experimental procedures are not described clearly throughout the manuscript. It is strongly recommended to add step-by-step protocols, especially for buffer exchange and washing steps, so that the experiments can be properly evaluated and reproduced. In addition, although the fluidic system is fundamentally different from a simple nanochannel, the analysis relies mainly on current responses while assuming that nanofluidic behavior has already been established.

Response: We thank the reviewer for recognizing our strategy using guided mycelium growth. We have revised the manuscript to improve the clarity of the experimental procedures. In particular, we have added detailed step-by-step descriptions of buffer exchange, washing procedures, and device preparation in the Methods section to ensure that the experiments can be properly evaluated and reproduced.

Regarding "*In addition, although the fluidic system is fundamentally different from a simple nanochannel, the analysis relies mainly on current responses while assuming that nanofluidic behavior has already been established.*"

We thank the reviewer for this important comment. We acknowledge that the mycelial network differs from a conventional solid-state nanochannel. In the revised manuscript, we clarify that ion transport occurs within the mycelial hyphae, which provide confined pathways for ion transport. The observed voltage-, concentration-, and pH-dependent transport characteristics are consistent with surface-charge-regulated ion transport in a nanofluidic regime, establishing nanofluidic behavior in the mycelial scaffold.

Specific comments are provided below.

1. Fig. 2b suggests substantial void volume between hyphae, yet the manuscript appears to assume that ion transport occurs primarily through nanofluidic hyphal networks. it would be helpful to clarify why transport through extracellular voids is excluded, and why the conductivity plateau versus concentration is attributed specifically to pathways inside hyphae.

Response: We thank the reviewer for this insightful comment regarding the mechanistic origin of the ion transport pathways.

In the revised manuscript, **we have clarified why ion transport is primarily associated with nanoconfined channels within the hyphae rather than the larger void spaces between hyphae.** As shown in Figure 2b, the extracellular voids are typically on the microscale (tens of micrometers), whereas the internal hyphal channels are confined to the nanoscale (Figure 2c). The ion conductivity enhancement observed at low electrolyte concentrations is characteristic of electric double layer (EDL)-regulated transport, which becomes significant only when the channel dimension approaches the Debye length[1, 2, 3]. Such nanoconfinement conditions are therefore unlikely to occur in the much larger void spaces between hyphae.

We have also included additional experimental clarification in the revised manuscript. First, mechanical compression of the mycelium network substantially reduces the void volume between hyphae, yet the measured ionic conductivity remains nearly unchanged (Supplementary Figure 11). This observation suggests that the microscale void spaces do not dominate the conduction pathway. Second, the hyphae form an interconnected network through hyphal fusion and extracellular matrix components, enabling direct transfer of water, ions, and metabolites within the network[4, 5]. Dye-tracing experiments (Supplementary Figure 12) further confirm that the mycelial network forms a continuous and interconnected structure, allowing ion migration along the hyphal network rather than through isolated gaps between hyphae.

Taken together, these observations support that the dominant ion transport pathway is associated with the nanoconfined channels within the hyphal network, which provides the geometric conditions necessary for EDL-regulated ion transport. **The corresponding explanation has been clarified in the revised manuscript:**

"The ion transport in the mycelial network is primarily associated with nanoconfined channels within the hyphae rather than microscale void spaces between hyphae. The spaces between hyphae observed in Figure 2b are typically on the order of tens of micrometers, which are substantially larger than the Debye length in dilute electrolytes (typically ~10–100 nm). Such large channels do not support electric double layer overlap and therefore cannot sustain nanofluidic transport. This interpretation is further supported by compression tests, where compressed samples with reduced void volume between hyphae exhibit ion conductivities comparable to those of uncompressed

samples (Supplementary Figure 11). The nanoporous structure within the hyphae is also expected to carry negative charge similar to the hyphal surface, likely arising from cell membrane disruption and the dissociation of functional groups, thereby allowing cation migration within the hyphal interior. In addition, dye-tracing experiments reveal that the mycelial hyphae form an interconnected network supporting continuous ion transport pathways (Supplementary Figure 12), enabled by hyphal fusion and extracellular matrix components45, 51." (page 7 line 164)

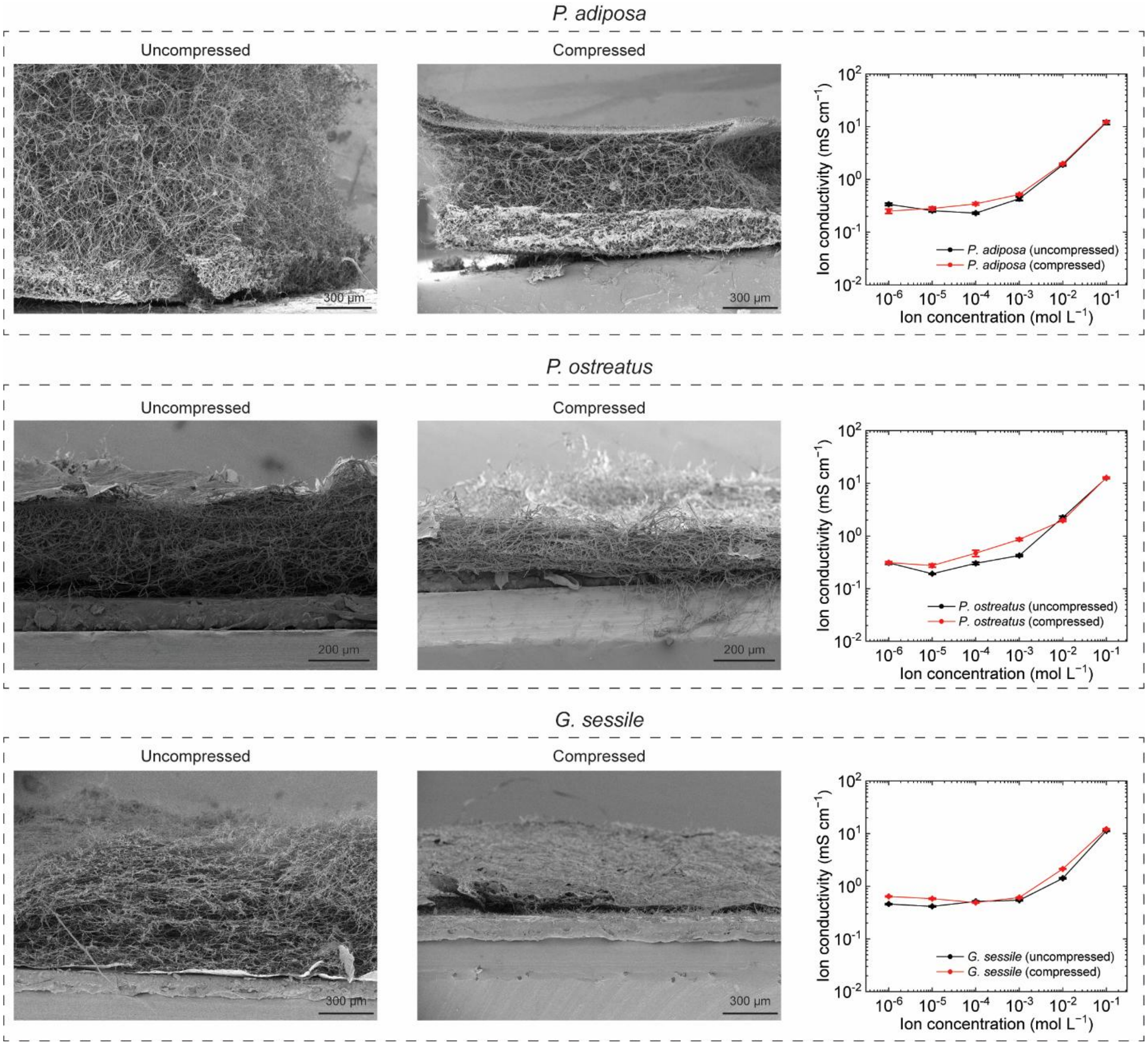

Supplementary Figure 11. SEM images of original (uncompressed) and compressed mycelium samples, together with the corresponding ion conductivity measurements. The compressed samples were subjected to a pressure of 6.9 MPa for 1 min, resulting in reduced void volume between hyphae. The comparable conductivity observed for uncompressed and compressed

samples suggests that the ion transport primarily occurs through the nanoconfined hyphal network rather than through microscale void spaces between adjacent hyphae.

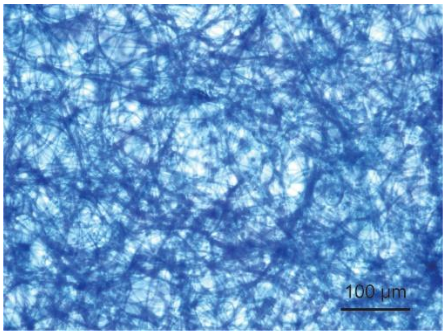

Supplementary Figure 12. Optical microscope image of the *P. adiposa* hyphal network stained with 1×10^{-4} mol L^{-1} methylene blue solution for dye-tracing experiments. The uniform staining indicates that the mycelial hyphae form an interconnected network with continuous transport pathways.

2. The manuscript does not provide time-dependent current data. For long nanofluidic channels, transient current measurements (I vs. t) under constant bias are generally expected to show delayed ion responses. Furthermore, It is recommended to discuss why no nonlinearity associated with ion concentration polarization appears in Fig. 4b.

Response: We thank the reviewer for this helpful comment. We indeed observed delayed ionic responses immediately after introducing the electrolyte. However, in our measurements the I–V curves were recorded only after the samples were fully immersed and equilibrated in the electrolyte, ensuring that the system had reached a steady state before the electrical characterization. **We have expanded discussion in the revised manuscript:** "The samples were fully immersed and equilibrated in the KCl electrolyte for at least 24 hours prior to measurement to ensure complete electrolyte exchange and avoid delayed ion response (Supplementary Figure 40), such that the system reached steady-state ion transport before electrical characterization." (page 22 line 535)

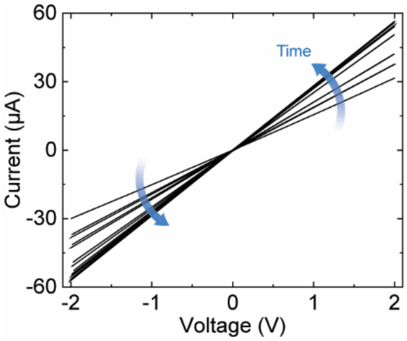

Supplementary Figure 40. Transient current–voltage response of *P. adiposa* mycelium immersed in 1×10^{-4} mol L^{-1} KCl solution. The ion transport shows a delayed response due to diffusion-limited equilibration within the mycelium. Therefore, in our measurements the samples were immersed in the KCl electrolyte for at least 24 hours prior to testing to ensure complete electrolyte equilibration.

Regarding "*Furthermore, It is recommended to discuss why no nonlinearity associated with ion concentration polarization appears in Fig. 4b.*"

We thank the reviewer for this insightful comment. The I–V curves shown in Figure 4e remain approximately linear without pronounced nonlinear behavior typically associated with ion concentration polarization. This is likely because ion transport in our system occurs through a distributed mycelial network rather than a single long nanochannel. The interconnected hyphal pathways provide multiple parallel ion transport routes with relatively large effective diameters, which reduces the formation of strong ion depletion zones and suppresses the development of concentration polarization. As a result, the I–V characteristics remain close to linear within the measured voltage range. The modest rectification ratio (~1.5) further suggests that the transport asymmetry is relatively weak and not dominated by strong concentration polarization.

We have expanded discussion in the revised manuscript: "The I–V curves do not show nonlinearity associated with concentration polarization, likely because ion transport occurs through a distributed mycelial network rather than a single long nanochannel. The interconnected hyphal pathways provide multiple parallel ion transport routes with relatively large effective diameters, which reduces the formation of strong ion depletion zones and suppresses the development of concentration polarization, resulting in moderate rectification." (page 15 line 341)

3. In Fig. 4a–c, achieving pH 3 requires approximately 10^−3 mM HCl, which is higher than the KCl concentration used. Therefore, ionic strength is not identical across pH conditions, and the ion-strength effect in Fig. 4d–f is not isolated from pH.

Response: We thank the reviewer for this careful observation. Achieving pH = 3 requires 1 mM HCl, which increases the ionic strength relative to the KCl background electrolyte and may therefore introduce a confounding ionic-strength effect. To avoid this complication, **we have updated Figure 4b–c** by removing the pH = 3 condition so that the ionic strength remains nearly identical across the pH conditions. The corresponding analysis has been updated, yielding a revised pH-gating ratio of up to 3.0-fold. The related figures and descriptions in the manuscript have been updated to reflect this change.

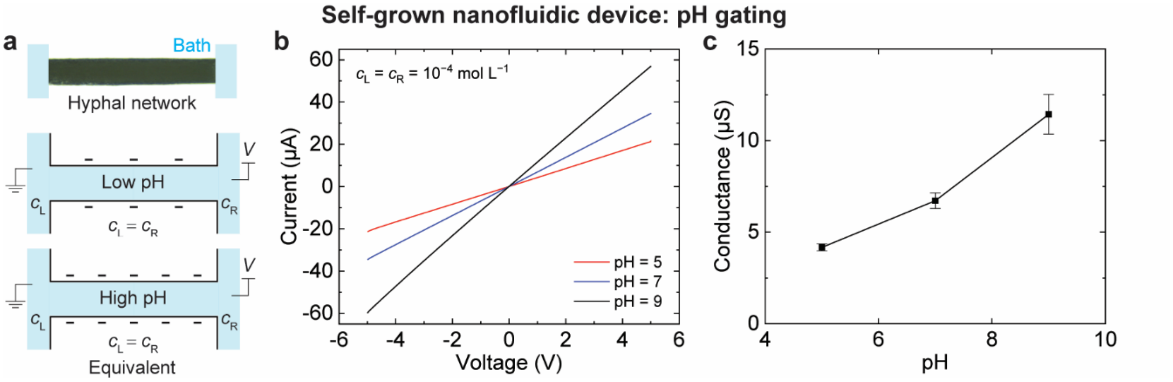

Figure 4. Performance and visualization of self-grown nanofluidic devices. (a–c) pH gating of a mycelium-integrated device. (a) Schematic illustrating that the negative surface charge can be tuned via pH. (b) Current–voltage response under varying pH. (c) The resultant conductance modulated by pH.

4. In Fig. 4d–f, rectification is shown only over a limited range and only under a large concentration difference. The authors are encouraged to clarify whether the platform can achieve stronger rectification, or to define the most appropriate application within the current performance range.

Response: We thank the reviewer for this insightful comment. The rectification ratio observed in the current device (~1.5) is modest compared to highly optimized solid-state nanofluidic diodes. This likely arises from the structural characteristics of the mycelial network, where ion transport

occurs through a distributed network of multiple parallel hyphal pathways rather than a single highly confined nanochannel. Such a multi-path architecture can reduce the degree of ion depletion and accumulation typically required for strong ionic rectification.

Nevertheless, the present platform offers a distinct capability: guiding the growth of living mycelium to form nanofluidic pathways across diverse geometries. Ionic current rectification in nanofluidic channels and nanopores is known to depend strongly on channel geometry, surface charge distribution, and electrolyte conditions, and moderate rectification is commonly observed in many nanofluidic systems[6, 7, 8]. Within the current performance range, this system may therefore be particularly suited for applications that benefit from adaptive or bio-integrated ionic transport, such as ion gating, environmental sensing, and living-material-based ion regulation, where moderate rectification combined with structural adaptability can enable functional regulation of ion transport.

We have clarified this point in the revised manuscript:

"The interconnected hyphal pathways provide multiple parallel ion transport routes with relatively large effective diameters, which reduces the formation of strong ion depletion zones and suppresses the development of concentration polarization, resulting in moderate rectification." (page 15 line 343)

"Although the rectification ratio is moderate, this bio-derived material platform may be particularly suited for applications that benefit from adaptive or bio-integrated ionic transport11, 57, 58, including ion gating, environmental sensing, and living-material-based ion regulation, where moderate rectification combined with structural adaptability can enable functional regulation of ion transport." (page 15 line 352)

5. In Fig. 4g–i, the connection between dye membrane penetration and nanofluidic transport across reservoirs is unclear. Furthermore, how is staining inside hyphae demonstrated while excluding possible surface staining?

Response: We thank the reviewer for this important comment. We clarify that the dye staining experiment is not intended to demonstrate nanofluidic transport across reservoirs, **but rather to**

visualize local ion enrichment within the mycelial network, which is later utilized for Ca^{2+} detection (Figure 4j–l).

To distinguish between possible surface adsorption and nanofluidic ion enrichment, we performed a washout test after staining (Supplementary Figure 31). If the staining were dominated by surface adsorption, the dye would be expected to remain bound to the hyphal surface after rinsing. In contrast, we observed that the enhanced methylene blue staining was largely removed during the washing step. This reversible staining behavior suggests that the dye accumulation arises from transient ion enrichment within confined pathways rather than irreversible surface adsorption.

In addition, the staining follows the filamentous morphology of the hyphae rather than appearing as a uniform coating on the outer surface (Supplementary Figure 34). Together, these observations suggest that the dye accumulation is associated with nanoconfined pathways within the mycelial structure, where electric double layer (EDL) effects and nanoconfinement can promote enrichment of cationic dye molecules such as methylene blue.

We have clarified this point in the revised manuscript:

"Besides electrical responses, the mycelial network can also be readily visualized using cationic dyes due to nanofluidic ion enrichment, which helps its application in multiple scenarios such as biosensing and diagnosis." (page 15 line 359)

"A washout test, in which the blue staining was largely removed after rinsing, suggests that the dye accumulation arises from transient ion enrichment within confined pathways rather than irreversible surface adsorption (Supplementary Figure 31). After freeze-drying, the mycelial scaffold retains negatively charged surfaces and a porous structure, which can promote enrichment of cationic dye molecules under nanoconfinement. The intrinsic porosity of the mycelial network further facilitates dye penetration along the filamentous hyphae, resulting in uniformly stained mycelium." (page 15 line 365)

"higher-magnification images show that the dye signal is distributed across the hyphae width rather than being confined to the outer boundary (Supplementary Figure 34), consistent with local cation enrichment within nanoconfined hyphal pathways." (page 16 line 384)

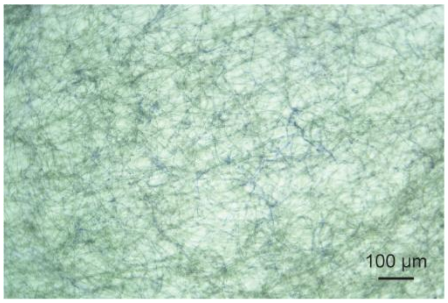

Supplementary Figure 31. Optical microscope image of the stained *P. adiposa* hyphal network after rinsing with deionized water for five minutes to remove methylene blue. The staining is significantly reduced compared with that observed before washout (Supplementary Figure 12), indicating that the signal arises from ion enrichment rather than irreversible adsorption.

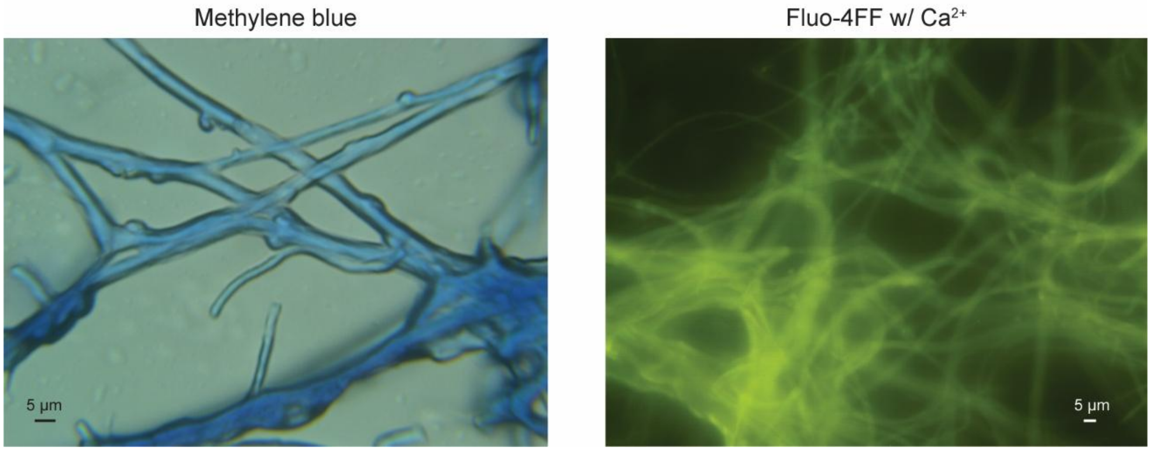

Supplementary Figure 34. Microscope images of stained *P. adiposa* mycelial hyphae. The dye signal follows the filamentous hyphal morphology and appears distributed across the hyphal width rather than being confined to the outer boundary, consistent with local cation enrichment within nanoconfined hyphal pathways rather than simple surface adsorption.

6. In Fig. 4j–l, it would be helpful to clarify where and when the images were acquired (e.g., at the entrance or in the center of the microchannel). Providing a full-field view of the microchannels would also help explain why enrichment occurs at that location.

Response: We thank the reviewer for this helpful comment. The fluorescence images were acquired at the center of the microchannel right after the introduction of Fluo-4FF solution. The

enrichment occurs along the hyphal network within the microchannel, where the nanoconfined pathways of the mycelium can locally concentrate cations. As a result, stronger fluorescence signals are observed in regions containing hyphae compared to the surrounding microchannel areas without mycelium. **We have clarified the imaging location and timing** in the revised manuscript. In addition, we have **supplemented the figure with wider-field images** showing the full microchannel to better illustrate the spatial distribution of the fluorescence signal and to explain why enrichment occurs at the hypha-containing regions.

"Wider-field images show uniform fluorescence enhancement in regions containing the mycelial network (Supplementary Figure 33), while higher-magnification images show that the dye signal is distributed across the hyphae width rather than being confined to the outer boundary (Supplementary Figure 34), consistent with local cation enrichment within nanoconfined hyphal pathways." (page 16 line 383)

"The mycelium-integrated microchannel was washed first to remove residual ions and then pre-soaked with Ca^{2+} solution at a specific concentration for one hour. Fluorescent images were acquired at the center of the microchannel immediately after introducing 1×10^{-6} mol L^{-1} Fluo-4FF solution without any applied voltage bias." (page 21 line 506)

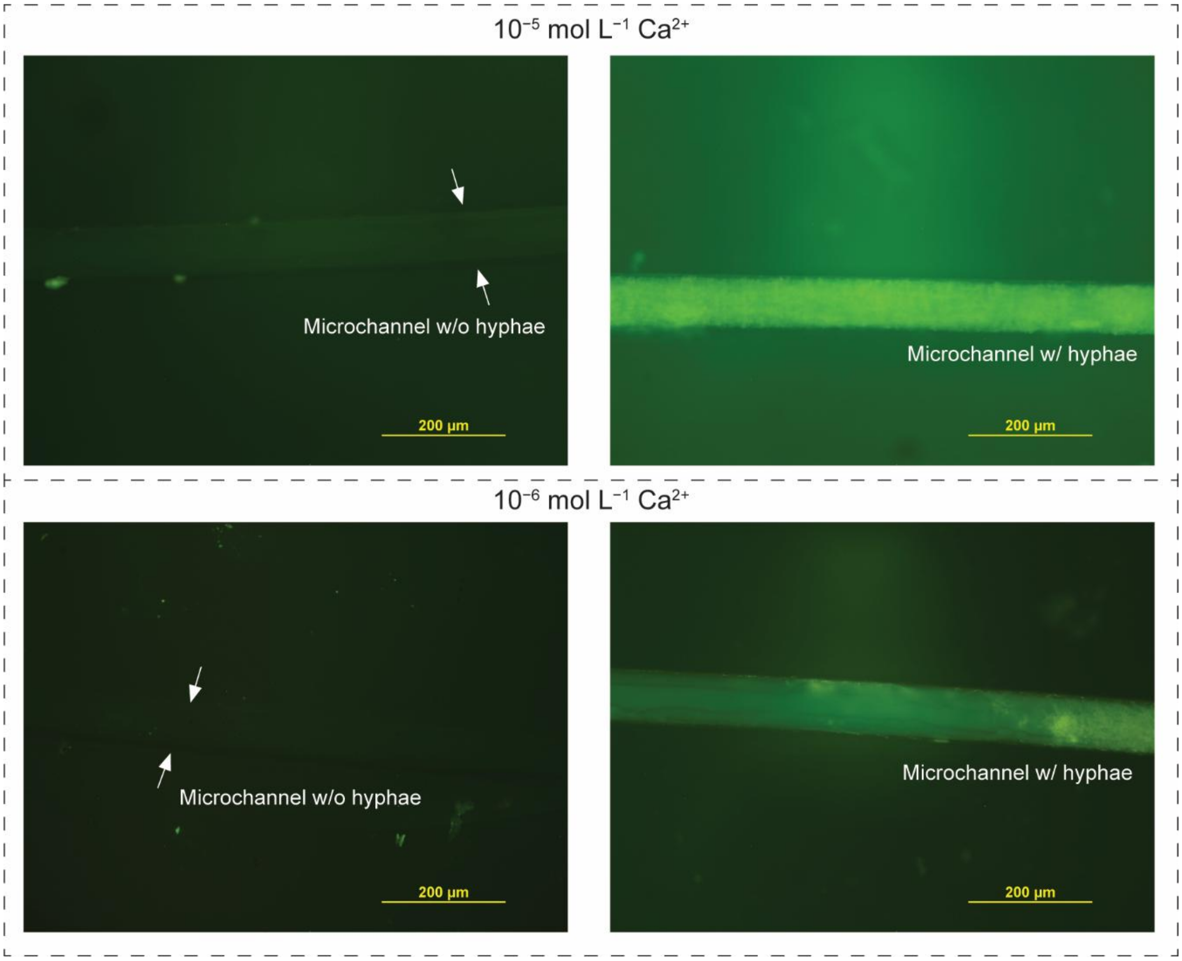

Supplementary Figure 33. Wide-field fluorescent images of the microchannel pre-soaked with Ca^{2+} immediately after exposure to Fluo-4FF. Enhanced fluorescence is observed in regions containing the mycelial network, consistent with nanofluidic cation enrichment within nanoconfined hyphal pathways.

7. A control experiment at 0 mM Ca2+ is needed. it would be clearer to clarify whether Fluo-4FF and Ca2+ were injected together or separately, whether a wash step was included, the enrichment duration, and the applied voltage bias.

Response: We thank the reviewer for raising these detailed questions regarding the experimental procedure. In response, **we have added a control experiment at 0 mM Ca^{2+}** (Supplementary Figure 32) and **clarified the experimental protocol** in the revised manuscript, including the

washing step, the sequence of Fluo-4FF and Ca^{2+} introduction, the enrichment duration, and the absence of an applied voltage bias.

Specifically, the microchannel was first washed to remove residual ions and then pre-soaked with the Ca^{2+} solution for one hour. The 1×10^{-6} mol L^{-1} Fluo-4FF solution was subsequently introduced into the microchannel, and fluorescence images were acquired at the center of the channel immediately after introduction. No external voltage bias was applied during the experiment.

The corresponding description has been added to the revised manuscript:

"However, the hyphae in the microchannel greatly enhance the fluorescent effect due to their nanofluidic ion enrichment capability (Figure 4j bottom), compared with the control condition containing Fluo-4FF but no Ca^{2+} (Supplementary Figure 32), thus expanding the detection range of Fluo-4FF." (page 16 line 380)

"The mycelium-integrated microchannel was washed first to remove residual ions and then pre-soaked with Ca^{2+} solution at a specific concentration for one hour. Fluorescent images were acquired at the center of the microchannel immediately after introducing 1×10^{-6} mol L^{-1} Fluo-4FF solution without any applied voltage bias." (page 21 line 506)

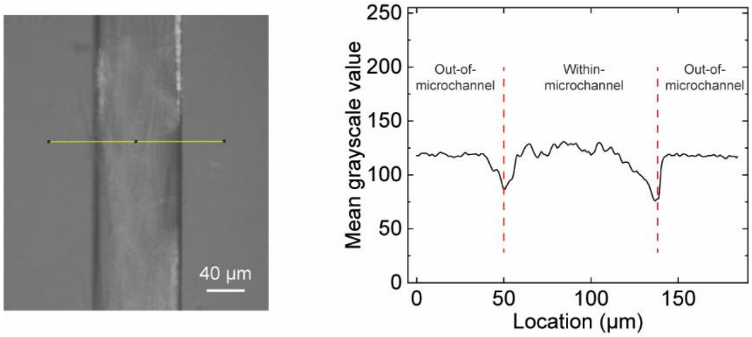

Supplementary Figure 32. Control fluorescence image of the mycelium-integrated microchannel containing the fluorescence indicator Fluo-4FF but no Ca^{2+}. The extracted grayscale image of the green channel shows no clear difference between the microchannel region and the surrounding area, likely because immersion of the mycelium in aqueous solution reduces optical scattering due to refractive index matching. The line profile shows that the grayscale value increases from ~120 out of the microchannel to ~130 within the microchannel, indicating that the scattering signal

without fluorescence is ~10 in grayscale value and contributes less than ~10% of the observed fluorescence enhancement.

8. It is also recommended to demonstrate that the enrichment indeed occurs inside hyphae.

Response: We thank the reviewer for this helpful suggestion. To further clarify the spatial location of the enrichment, **we have supplemented microscope images** of hyphae stained with methylene blue **and fluorescence images** obtained using Fluo-4FF in the presence of Ca^{2+}. These images show that the dye signal follows the filamentous hyphal morphology and appears distributed across the hyphal width rather than being confined to the outer boundary, which is consistent with local cation enrichment within nanoconfined hyphal pathways rather than simple surface adsorption.

We have expanded discussion in the revised manuscript: "Wider-field images show uniform fluorescence enhancement in regions containing the mycelial network (Supplementary Figure 33), while higher-magnification images show that the dye signal is distributed across the hyphae width rather than being confined to the outer boundary (Supplementary Figure 34), consistent with local cation enrichment within nanoconfined hyphal pathways." (page 16 line 383)

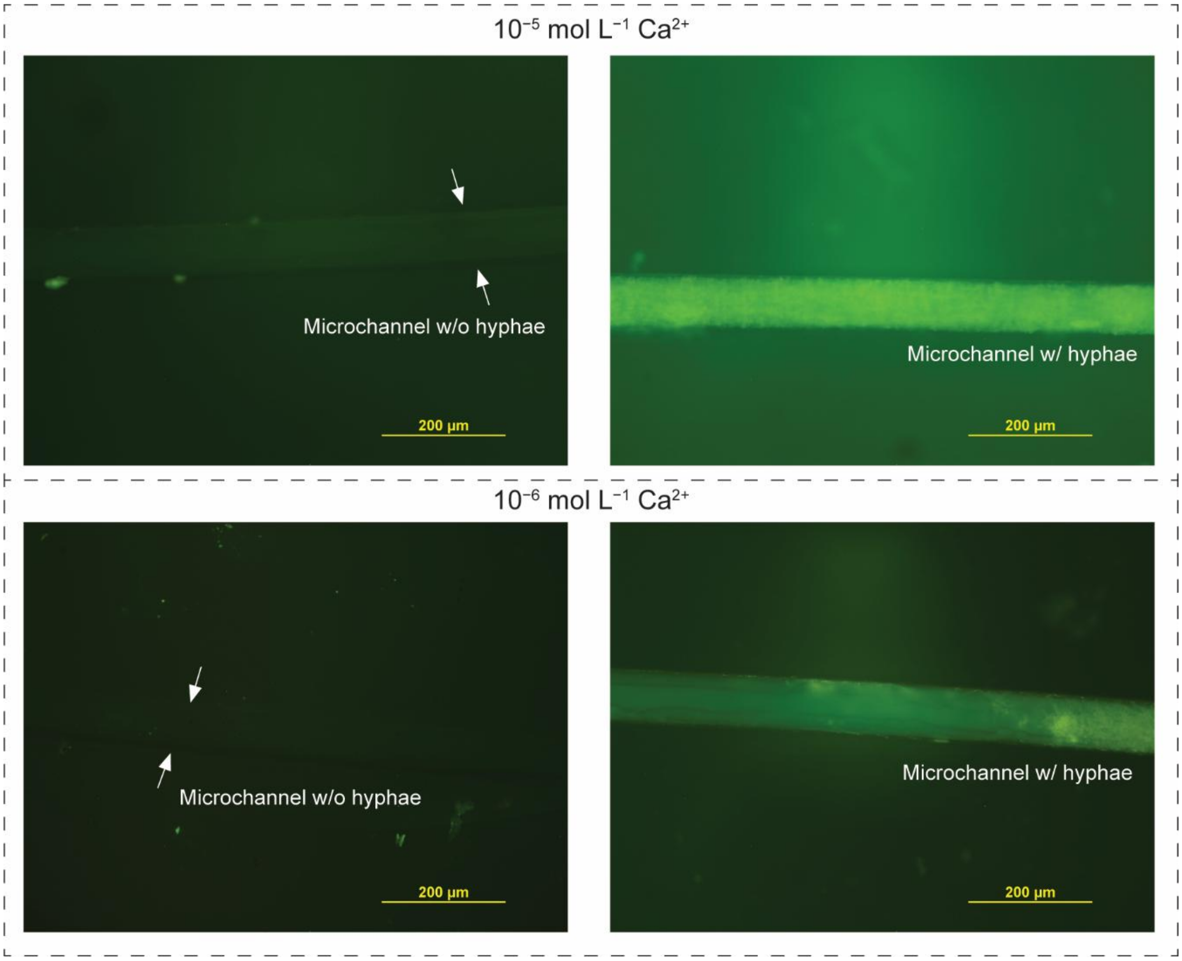

Supplementary Figure 33. Wide-field fluorescent images of the microchannel pre-soaked with Ca^{2+} immediately after exposure to Fluo-4FF. Enhanced fluorescence is observed in regions containing the mycelial network, consistent with nanofluidic cation enrichment within nanoconfined hyphal pathways.

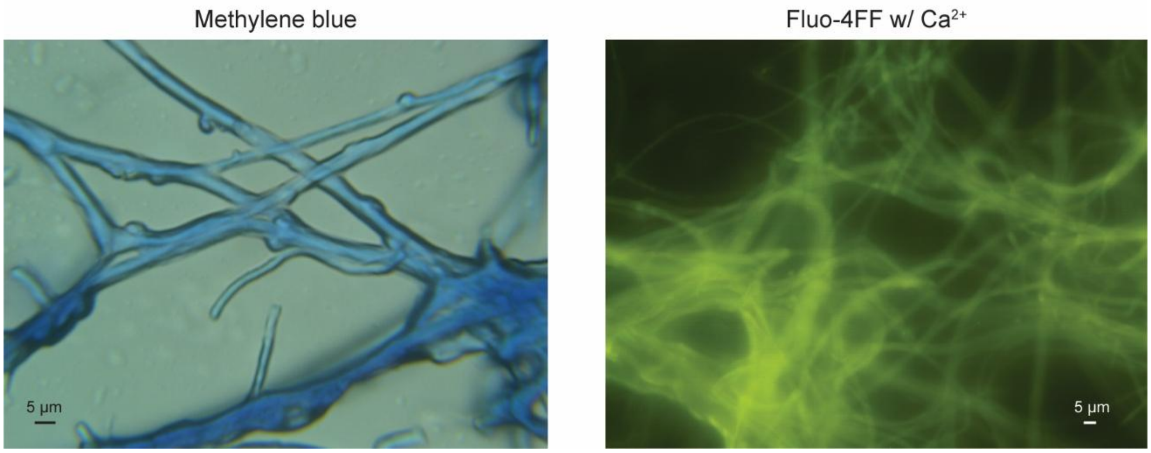

Supplementary Figure 34. Microscope images of stained *P. adiposa* mycelial hyphae. The dye signal follows the filamentous hyphal morphology and appears distributed across the hyphal width rather than being confined to the outer boundary, consistent with local cation enrichment within nanoconfined hyphal pathways rather than simple surface adsorption.

9. In Fig. S10, it remains difficult to determine whether the channel is fully filled with mycelium or only surface-covered.

Response: We thank the reviewer for raising this point. The microchannel is fully filled with mycelial hyphae, which can be directly observed from the optical microscope images (Figure 3d), where the hyphae grow continuously along the entire microchannel. The SEM images in Supplementary Figure 18 (originally Supplementary Figure 10) do not clearly show the fully filled structure because SEM sample preparation requires detaching the PDMS microchannel from the glass substrate and exposing the internal channel surface. During this process, part of the hyphal network can be mechanically disturbed or removed, leading to an incomplete appearance in the SEM images.

We have expanded our discussion in Supplementary Figure 18: "The hyphal network fills the microchannel, as shown by the optical microscope images (Figure 3d). It is noted that the SEM images here do not clearly show the fully filled structure because part of the hyphal network can be mechanically disturbed or removed when the PDMS microchannel is detached from the glass substrate for SEM imaging." (Supplementary Figure 18 caption)

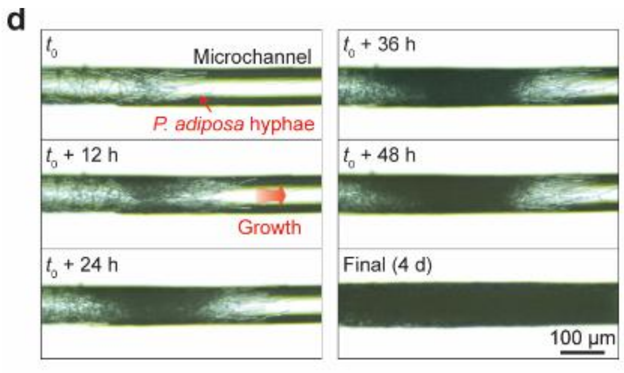

Figure 3. Self-grown mycelium-enabled nanofluidics. (d) Time-lapse microscope images of mycelium growth through the microchannel.

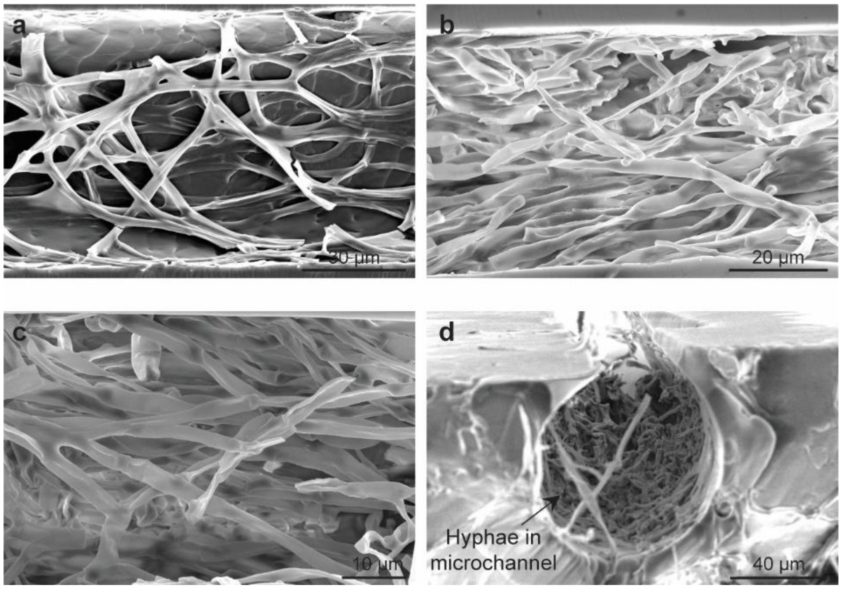

Supplementary Figure 18. SEM images of self-grown *P. adiposa* in the microchannel, forming an interwoven hyphal network that enables functional nanofluidic ion transport. The hyphal network fills the microchannel, as shown by the optical microscope images (Figure 3d). It is noted that the SEM images here do not clearly show the fully filled structure because part of the hyphal network can be mechanically disturbed or removed when the PDMS microchannel is detached from the glass substrate for SEM imaging.

10. Please explain how pore size was derived from SEM images, and whether this method is directly comparable to the size estimates in Fig. 3e, 3i, and 3l.

Response: We thank the reviewer for the questions. The pore size D_{pore} was estimated from SEM images by measuring the pore area A_{pore} and assuming an equivalent circular pore, where $A_{\mathrm{pore}} = \pi / 4 \times D_{\mathrm{pore}}^2$. The pore areas were extracted from the SEM images using ImageJ, and the pore size distribution was obtained using kernel density estimation of the calculated pore size data. **We have clarified this procedure in the Methods section**: "The pore size D_{pore} was estimated from SEM images by measuring the pore area A_{pore} and assuming an equivalent circular pore, where $A_{\mathrm{pore}} = \pi / 4 \times D_{\mathrm{pore}}^2$. The pore areas were extracted from the SEM images using ImageJ, and the pore size distribution was obtained using kernel density estimation of the calculated pore size data." (page 21 line 500)

Regarding "*whether this method is directly comparable to the size estimates in Fig. 3e, 3i, and 3l*"

We thank the reviewer for this comment. Regarding the relationship between the pore size distribution and the extracted equivalent nanochannel size, all three species exhibit a broad distribution of cross-sectional nanopores smaller than ~100–150 nm. These nanopores, together with the intrinsic nanopores within the mycelial cell wall (2–3 nm[9]), collectively contribute to equivalent nanochannel sizes of 49.7 nm, 21.3 nm, and 7.4 nm for the three configurations shown in Figure 3.

Importantly, the expression used to estimate the nanochannel size $\kappa = q(\mu_+ + \mu_-)CN_{\mathrm{A}} + 2\sigma_{\mathrm{s}}\mu_+ / d$ was originally derived for an idealized single cylindrical nanochannel. In contrast, the mycelium forms a nanoporous scaffold where ion transport occurs through a distributed hyphal network rather than a single long nanochannel. The interconnected hyphal pathways therefore provide multiple parallel ion transport routes. Consequntly, the fitted nanochannel size on the order of tens of nanometers should not be interpreted as the physical diameter of an individual pore, but rather as an effective averaged transport channel that captures the collective behavior of this parallel and tortuous nanochannel network. We therefore describe d as an average effective channel dimension that reflects ion transport in this interconnected nanoporous architecture.

We have expanded our discussion in the revised manuscript:

“Together, the larger internal pores and the intrinsic cell-wall nanopores enable the hyphae to function as an interconnected nanoporous network for ion transport.” (page 6 line 150)

“It should be noted that this expression was originally derived for an idealized single cylindrical nanochannel. In contrast, mycelium forms a nanoporous scaffold in which ion transport occurs through a distributed hyphal network with multiple parallel pathways rather than a single long nanochannel, resulting in collective transport behavior within this interconnected nanoporous architecture.” (page 8 line 186)

“Using *P. adiposa* with a surface charge of -2.8 mC m^{-2}, conductivity modelling indicates that the mycelium-integrated microchannel is equivalent to a 49.7 nm nanochannel in terms of ion transport (Supplementary Note 2). This effective channel size reflects the collective transport behavior of nanopores within the mycelial cell walls (~several nm) and the hyphal interior (~10–100 nm).” (page 11 line 260)

“Conductivity modeling indicates an equivalent nanochannel size of 7.4 nm, consistent with the observation that *P. ostreatus* contains a higher proportion of smaller nanopores (Supplementary Figure 7).” (page 12 line 286)

11. Additional detail is needed regarding DI washing and buffer exchange in these long nanofluidic systems, including how completion of washing was determined and whether residual nutrients, pre-existing ions, or materials released from hyphae could affect the results.

Response: We thank the reviewer for this helpful suggestion. We agree that washing and electrolyte exchange are important for ensuring reliable measurements in long nanofluidic systems. **We have supplemented additional details** regarding the washing procedure, the criterion used to determine completion of washing, and the electrolyte equilibration process, as described below.

“The samples were washed with deionized water to remove residual ions until the conductivity of the rinsed water was lower than 20 μS cm^{-1}.” (page 22 line 523)

“For microchannel samples, deionized water was introduced at both ends of the channel and the liquid was repeatedly removed using Kimwipes to facilitate flushing. The process was repeated until the conductivity of the deionized water at both ends of the microchannel was below 20 μS cm^{-1}.” (page 22 line 528)

"The samples were fully immersed and equilibrated in the KCl electrolyte for at least 24 hours prior to measurement to ensure complete electrolyte exchange and avoid delayed ion response (Supplementary Figure 40), such that the system reached steady-state ion transport before electrical characterization." (page 22 line 535)

Regarding "*whether residual nutrients, pre-existing ions, or materials released from hyphae could affect the results*"

We performed additional control experiments using freeze-dried mycelium without the washing procedure to examine the influence of residual nutrients, pre-existing ions, and materials released from hyphae. Without washing, the intrinsic nanofluidic transport behavior is largely masked by these uncontrolled species.

"This step minimizes the influence of residual nutrients, pre-existing ions, and materials released from the hyphae that could otherwise introduce uncontrolled variables and mask the intrinsic nanofluidic transport behavior (Supplementary Figure 39)." (page 22 line 525)

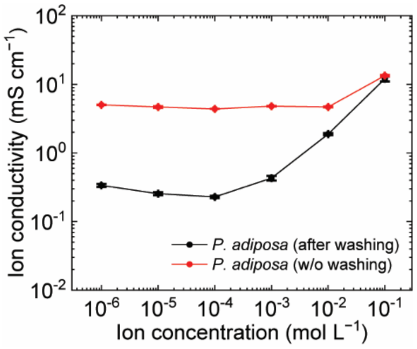

Supplementary Figure 39. Ion conductivity of *P. adiposa* mycelium with and without the washing procedure. Without washing, residual nutrients, pre-existing ions, and materials released from the hyphae can mask the intrinsic nanofluidic ion transport behavior.

12. The experimental details are insufficient for reproducibility. For example, measurement parameters of the equipment, detailed device geometry, copper wire removal from the microfluidic device, and freeze-drying conditions should be provided.

Response: We thank the reviewer for this important suggestion regarding the experimental details and reproducibility. **We have supplemented additional details** on the I–V measurement parameters, microfluidic device fabrication, device geometry, and freeze-drying conditions.

"The electrical response was measured using linear sweep voltammetry (LSV) on a BioLogic VSP potentiostat. After a rest time of 5 s, the potential was scanned from –2.0 V to 2.0 V at a scan rate of 100 mV s^{-1}. Ag/AgCl electrodes with a diameter of 4 mm were used (Supplementary Figure 14). The samples were fully immersed and equilibrated in the KCl electrolyte for at least 24 hours prior to measurement to ensure complete electrolyte exchange and avoid delayed ion response (Supplementary Figure 40), such that the system reached steady-state ion transport before electrical characterization." (page 22 line 532)

"The microchannel was fabricated by casting PDMS onto a copper wire (~88 μm in diameter and several centimeters in length). The two ends of the wire were wrapped with thermal adhesive tape (0.3 mm thick) to form small reservoirs, while the unwrapped section (~1 cm long) served as the microchannel template. After curing, the patterned PDMS was peeled off from the copper wire and bonded to a glass slide via oxygen plasma after trimming and hole drilling. Approximately 30 μL of solid or liquid nutrients were then added to one reservoir of the customized PDMS microfluidic device, and 10 μL of mycelium liquid culture was inoculated to enable growth through the microchannel for subsequent conductivity testing (Supplementary Figure 15). The samples were cultivated for a sufficiently long incubation time (>10 days) to ensure a dense hyphal network within the microchannel, thereby maintaining measurement consistency." (page 20 line 477)

"As for the other two configurations, the gap was created by placing aluminum wires (0.3 mm in diameter) as spacers between a glass sheet (75 mm × 75 mm) and a glass slide (75 mm × 25 mm). Approximately 1 mL of solid nutrients were placed outside the gap, and liquid nutrients were placed within the gap to allow mycelium to grow from one side to the other, constructing nanofluidic pathways through the gap. The volumetric mycelium-integrated device was fabricated by placing solid nutrients into two discrete tanks (5 cm × 3 cm × 0.5 cm) separated by a stainless steel mesh (holes ~400 mesh) and inoculating 100 μL of mycelium liquid culture, enabling mycelium to grow through the mesh and form nanofluidic pathways between the two tanks. All devices were freeze-dried at –57 °C under a vacuum below 20 Pa for over 24 hours to preserve nanoporosity within the mycelial hyphae for nanofluidic ion transport." (page 20 line 487)

"The mycelium grown on the solid medium was first frozen in a –20 °C freezer for 24 hours and subsequently freeze-dried using a LABCONCO FreeZone 2.5 lyophilizer at –57 °C under a pressure below 20 Pa for over 24 hours." (page 20 line 471)

13. The manuscript states that previous methods have multiple drawbacks, but the basis for each claim is not sufficiently justified. It would improve the manuscript to specify which methods are limited in geometric programmability, throughput, and scalability. It would also be helpful to clarify how the present approach improves on them.

Response: We thank the reviewer for this helpful suggestion. We agree that the limitations of existing nanofluidic fabrication strategies should be more clearly specified. In the revised manuscript, **we have clarified which classes of nanofluidic platforms are associated with limitations** in geometric programmability, fabrication throughput, and scalability, and have **further clarified how the proposed approach addresses these limitations.** The corresponding discussion has been revised in the Introduction as follows:

"Multiple material families (e.g., silicon18, 19, alumina20, 21, and polymers8, 22) have been widely explored for nanofluidic devices and have become well-established platforms for investigating nanoscale transport. These systems are commonly fabricated using top-down techniques, such as electron-beam or photolithographic patterning followed by etching to define nanopores or nanochannels in dielectric substrates. While these approaches provide high structural precision and surface control, they typically require complex cleanroom processing, exhibit relatively low fabrication throughput, and are largely confined to rigid planar geometries, limiting scalability and geometric adaptability." (page 2 line 46)

"In contrast, bottom-up strategies—including the assembly of low-dimensional materials (e.g., graphene slit channels4, 16, 23, carbon nanotube membranes24, 25, and boron nitride nanochannels26) and biomolecular structures such as DNA nanopores27, 28 and protein nanopores10—offer broader material diversity and intrinsic molecular-scale confinement. However, graphene and boron nitride slit-channel devices often rely on mechanically assembled heterostructures with limited geometric programmability, while carbon nanotube membranes and biomolecular nanopores are typically difficult to fabricate in high throughput or to integrate into interconnected nanofluidic transport networks over large or non-planar surfaces." (page 2 line 53)

"In many of these nanofluidic platforms, surface-charge-governed ion transport phenomena such as selectivity4, 5, rectification6, 7, and pH-dependent conductance29, 30 have been widely investigated. These effects are commonly observed in charged nanopores12, 22, 29, nanochannels31, 32, 33, and nanoporous membranes4, 12, 34 used for ion-selective transport. However, these systems are typically based on predefined nanoscale architectures or synthetic nanoporous matrices fabricated through lithography or membrane assembly, which limits their ability to autonomously generate interconnected ion transport pathways within irregular three-dimensional confinements. Overcoming these constraints calls for alternative fabrication paradigms that simultaneously deliver nanofluidic functionality, scalability, and geometric adaptability." (page 2 line 61)

"Biomaterials offer a promising opportunity for constructing microfluidic and nanofluidic networks35, 36, 37 owing to their intrinsic hierarchical architectures and growth-enabled connectivity. A variety of bio-derived or bio-templated transport systems have been explored to guide molecular and ionic transport, including porous membranes fabricated from natural biomaterials37, 38, transport pathways formed within bacterial biofilms39, 40, and vascular transport architectures found in plant tissues such as leaf veins41. However, most of these approaches rely on static biological templates or processed biomaterials and therefore do not exploit the intrinsic growth capability of living systems to autonomously generate interconnected transport pathways. Here, we introduce fungal mycelium as a living, low-cost, and scalable bio-derived scaffold for constructing nanofluidic pathways that can adapt to confined geometries without lithographic definition of nanoscale features. This capability arises from its bottom-up self-growth, hyphal branching and anastomosis, hierarchical porosity, and negatively charged surfaces." (page 3 line 70)

Minor

1. In Fig. 1e, the left-to-right transformation is not easy to follow. it would be helpful to clarify whether the panels show different sections or different time points.

Response: We thank the reviewer for this helpful suggestion. **We have revised Figure 1e** to improve the clarity of the left-to-right transformation. The panels are now organized to clearly

indicate the progression of the process, from the initial state to the stages after incubation and freeze-drying. The labels and caption have also been revised accordingly.

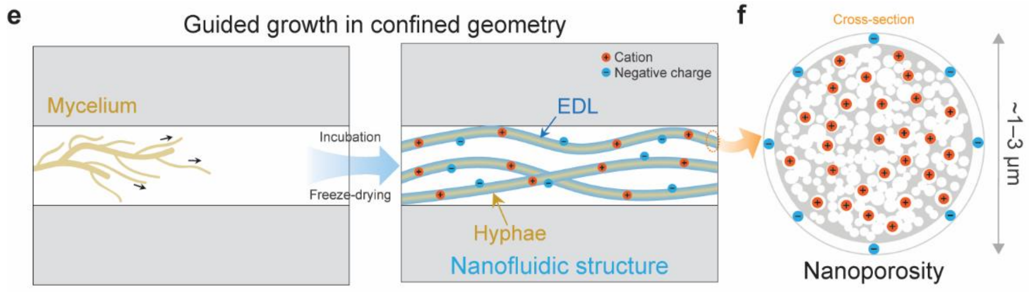

Figure 1. Ubiquitous and scalable mycelium and its guided growth in confined geometries. (e) Schematic of guided mycelium growth in a confined geometry, driven by growth resources such as nutrients and oxygen. The extended self-grown hyphae emerge as nanofluidic structures after freeze-drying, forming electric double layers (EDLs) that regulate cations, thus enabling nanofluidics. (f) Schematic of a cross-sectional hypha, showing its nanoporosity. The mycelial cell wall is inherently negatively charged due to the dissociation of functional groups, with nanopores within the hypha serving as pathways for cations.

2. Please define the time corresponding to "Final" in Fig. 3b.

Response: We thank the reviewer for this comment. In the revised manuscript, the original Fig. 3b has been reorganized and is now presented as Fig. 3d. The label "Final" has been updated to "Final (4 d)" to clearly indicate the corresponding time point.

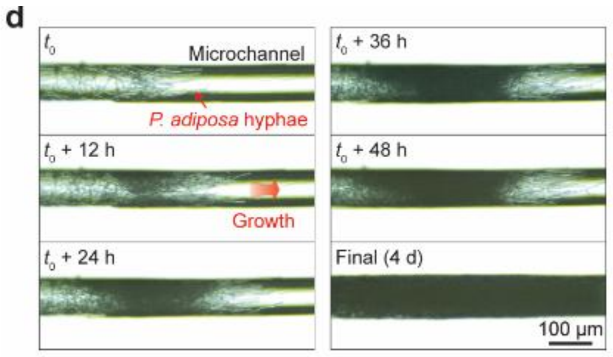

Figure 3. Self-grown mycelium-enabled nanofluidics. (d) Time-lapse microscope images of mycelium growth through the microchannel.

3. The microfluidic devices shown in Fig. 3a and Supplementary Fig. 8 appear slightly different.

Response: We thank the reviewer for this careful observation. In the revised manuscript, the schematic in Supplementary Figure 15 (originally Supplementary Figure 8) has been updated to match the microfluidic device shown in Figure 3c.

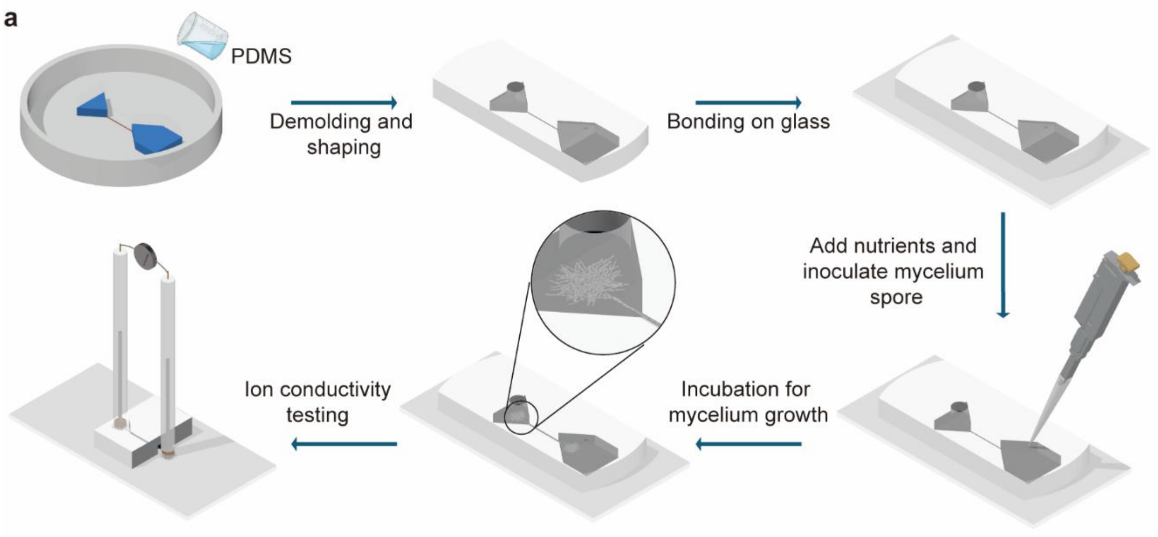

Supplementary Figure 15. Fabrication of the microchannel device using a copper wire. (a) The microchannel interconnects a reservoir containing nutrients and mycelium spores with an open space exposed to ambient air. Oxygen drives the mycelium to grow through the microchannel until it is fully filled.

4. It would also be clearer to state the use of KCl in the Results section, rather than only in the Methods section.

Response: We thank the reviewer for this helpful suggestion. In the revised manuscript, we have clarified in the Results section that KCl was used as the electrolyte, in addition to the description already provided in the Methods section.

"KCl electrolyte solutions at different concentrations were used for conductivity measurements throughout this study. The negative surface charge, synergistic with hyphal ion pathways, contributes to surface-mediated K^+ transport in the mycelial scaffold and confers a common multi-species ion regulation capability (Figure 2e and Supplementary Figures 8–10), a typical

nanofluidic characteristic where high ion conductivity is observed even at low ion concentrations (< 1×10^{-3} mol L^{-1}).” (page 7 line 158)

“(e) Ion conductivity measurements (Supplementary Figure 14) of the three mycelium membranes show substantially higher conductivity than bulk values at KCl concentrations below 1×10^{-3} mol L^{-1}, illustrating their universal ion regulation capability.” (Figure 2 caption)

“Thanks to the nanofluidic properties of mycelium, the integrated microchannel exhibits previously absent ion regulation capability (Figure 3g) under the same KCl electrolyte conditions.” (page 10 line 237)

Reviewer #3 (Remarks to the Author):

This manuscript, entitled " Self-Grown Mycelium in Confined Geometries as Nanofluidic Devices" is logically structured and supported by substantial experimental data. The authors demonstrate that ion transport through fibrous networks formed within a mycelium-integrated microchannel exhibits nanofluidic characteristics. However, based on the current evidence, it remains unclear whether the results fully substantiate the broad claims stated in the abstract, particularly that " by bypassing lithography and energy-intensive processing, this bio-derived strategy creates living and self-organizing ion transport networks with broad potential in sensing, ionic computing, and energy conversion."

Response: We thank the reviewer for this constructive comment. We agree that the scope of the claims in the abstract should accurately reflect the level of experimental evidence presented in the manuscript. Our intention was to highlight the potential of the mycelium-based nanofluidic platform as a bio-derived strategy for constructing ion transport networks without lithographic fabrication.

To improve clarity and avoid overinterpretation, **we have revised the abstract** to better align the claims with the experimental demonstrations in this work. In particular, we clarify that the present study demonstrates the formation of self-grown nanofluidic pathways and their ion regulation behaviors (e.g., conductivity enhancement, pH gating, and cation enrichment), while applications such as sensing, ionic computing, and energy conversion are discussed as possible future directions rather than direct demonstrations.

The corresponding statement in the abstract has been revised as follows:

"By bypassing lithography and energy-intensive processing, this bio-derived strategy may enable living and self-organizing ion transport networks with potential applications in sensing, ionic computing, and energy conversion." (page 1 line 30)

While the concept is interesting and potentially impactful, the present data do not yet demonstrate sufficient mechanistic understanding or controllability to justify such wide-ranging implications. Additional theoretical or experimental support would strengthen the manuscript and better align

the conclusions with the evidence provided before publication in Nature Communications. The main issues that require clarification are as follows:

Response: We thank the reviewer for this thoughtful assessment of our work and for recognizing the potential impact of the concept. In the revised manuscript, we have revised the discussion to better align the conclusions with the experimental evidence and to strengthen the mechanistic interpretation of the results. Specifically, we have added additional optical characterization, expanded the analysis of controllability, and included further conductivity and rectification measurements to improve quantification, reproducibility, and interpretation.

These revisions help clarify the mechanism and operational characteristics of the mycelium-based nanofluidic system while maintaining a balanced discussion of its broader implications. The specific concerns raised by the reviewer are addressed in detail below.

1. The introduction provides a thorough overview of mycelium-related studies. However, the discussion of micro-/nano-fluidic systems is relatively limited. It would help readers if the authors included a brief discussion on the history of biomimetic transport or other engineered approaches for precise control of ion and molecular transport, particularly in analogous bio-derived or bio-templated channel systems. Clarifying how the present work differs conceptually and technically from existing biomimetic ion transport platforms would help define its originality more clearly.

Response: We thank the reviewer for this helpful suggestion. In the revised Introduction, **we have added a brief discussion of bio-derived and bio-templated transport systems,** including porous membranes from natural biomaterials, transport pathways in bacterial biofilms, and vascular transport architectures in plant tissues. We also **clarify the conceptual distinction of our approach,** which exploits the intrinsic growth capability of living mycelium to autonomously generate interconnected transport pathways and adapt to confined geometries. The relevant text has been added to the Introduction:

"Biomaterials offer a promising opportunity for constructing microfluidic and nanofluidic networks35, 36, 37 owing to their intrinsic hierarchical architectures and growth-enabled connectivity. A variety of bio-derived or bio-templated transport systems have been explored to guide molecular and ionic transport, including porous membranes fabricated from natural

biomaterials37, 38, transport pathways formed within bacterial biofilms39, 40, and vascular transport architectures found in plant tissues such as leaf veins41. However, most of these approaches rely on static biological templates or processed biomaterials and therefore do not exploit the intrinsic growth capability of living systems to autonomously generate interconnected transport pathways. Here, we introduce fungal mycelium as a living, low-cost, and scalable bio-derived scaffold for constructing nanofluidic pathways that can adapt to confined geometries without lithographic definition of nanoscale features. This capability arises from its bottom-up self-growth, hyphal branching and anastomosis, hierarchical porosity, and negatively charged surfaces." (page 3 line 70)

2. The data regarding the mycelium membrane is convincing. However, the reproducibility and controllability of mycelium growth within confined microchannels require deeper discussion. Although Fig. S9 touches on growth rate, it would be helpful to discuss the extent to which network morphology can be controlled. A more fundamental question concerns the origin of the observed ion transport: is it primarily governed by the external EDL formed around the fibrous network (as in Fig. 1e), or does ion transport occur through the interior of the hyphae (as illustrated in Fig. 1f)? A comparison between live and freeze-dried mycelium could help distinguish between these possible transport pathways and clarify the underlying mechanism.

Response: We thank the reviewer for these insightful comments. In the revised manuscript, we have **expanded the discussion on the reproducibility and controllability** of mycelial network morphology. We have also **clarified the origin of ion transport** through additional control experiments, including comparisons between uncompressed and compressed samples, and between live and freeze-dried mycelium, to distinguish possible transport pathways. The specific responses are provided below.

Regarding "*The data regarding the mycelium membrane is convincing. However, the reproducibility and controllability of mycelium growth within confined microchannels require deeper discussion. Although Fig. S9 touches on growth rate, it would be helpful to discuss the extent to which network morphology can be controlled.*"

We thank the reviewer for this valuable comment on the reproducibility and controllability of mycelium growth. Under defined growth conditions, mycelium consistently forms interconnected

fibrous networks within confined geometries through hyphal tip extension, branching, and anastomosis. Comparable network formation is observed across different mycelium species (*G. sessile* and *P. adiposa*), microchannel geometries (rectangular and cylindrical), and growth environments (aerial, submerged, and mixed), as shown in Supplementary Videos 1–4. These observations indicate that mycelial networks reproducibly form within confined microchannels under a range of growth conditions.

- Supplementary Video 1: submerged *G. sessile* growth in a rectangular microchannel
- Supplementary Video 2: aerial *P. adiposa* growth in a cylindrical microchannel
- Supplementary Video 3: submerged *P. adiposa* growth in a cylindrical microchannel
- Supplementary Video 4: mixed aerial–submerged *P. adiposa* growth in a cylindrical microchannel

In addition to forming one-dimensional networks within microchannels, the mycelium can also grow into membranes and foams after longer incubation times (Supplementary Figure 24), providing greater control over the dimensionality of the resulting structures.

We have expanded the discussion in the revised manuscript:

"Comparable network formation is observed across different mycelium species (*G. sessile* and *P. adiposa*), microchannel geometries (rectangular and cylindrical), and growth environments (aerial, submerged, and mixed), as shown in Supplementary Videos 1–4, indicating reproducible formation of mycelial networks within confined microchannels across a range of growth conditions." (page 10 line 227)

"Beyond the aforementioned geometries, mycelium also possesses the capability to self-assemble into membranes and foams (Supplementary Figure 24), enabling greater control over the dimensionality of the resulting structures." (page 12 line 292)

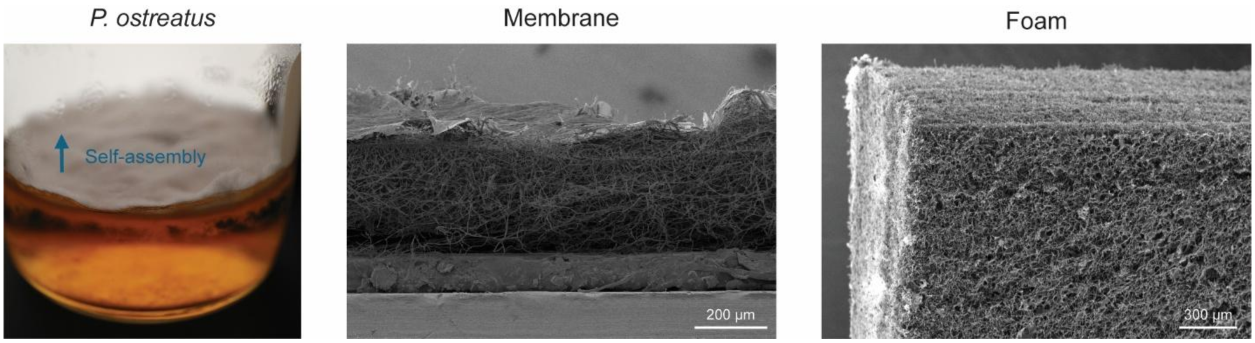

Supplementary Figure 24. Biological self-assembly of mycelium into membrane and foam structures, illustrating the dimensional versatility of the material.

Regarding "*A more fundamental question concerns the origin of the observed ion transport: is it primarily governed by the external EDL formed around the fibrous network (as in Fig. 1e), or does ion transport occur through the interior of the hyphae (as illustrated in Fig. 1f)? A comparison between live and freeze-dried mycelium could help distinguish between these possible transport pathways and clarify the underlying mechanism.*"

In the revised manuscript, we have clarified **that ion transport is primarily associated with nanoconfined channels within the hyphae** rather than around the hyphae, and have **revised Figure 1e–f** accordingly. We have also **included additional experimental evidence** in the revised manuscript. First, mechanical compression of the mycelium network substantially reduces the void volume between hyphae, yet the measured ionic conductivity remains nearly unchanged (Supplementary Figure 11). This observation suggests that the microscale void spaces around the hyphae do not dominate the conduction pathway. Second, the hyphae form an interconnected network through hyphal fusion and extracellular matrix components, enabling direct transfer of water, ions, and metabolites within the network[4, 5]. Dye-tracing experiments (Supplementary Figure 12) further confirm that the mycelial network forms a continuous and interconnected structure, allowing ion migration along the hyphal network rather than through isolated gaps between hyphae. Furthermore, comparisons between live and freeze-dried mycelium (Supplementary Figure 13) further support this interpretation, because the nanoporosity generated during freeze-drying is necessary for EDL-mediated ion transport in the nanoconfined structures.

Taken together, these observations support the conclusion that the dominant ion transport pathway is associated with the nanoconfined channels within the hyphal network, which provides the geometric conditions necessary for EDL-regulated ion transport. **The corresponding explanation has been clarified in the revised manuscript:**

"Leveraging its extensibility and hyphal anastomosis, mycelium can be guided to fill customized confined spaces and grow into desired device architectures (Figure 1e). The extended hyphae naturally exhibit nanoscale porosity and negatively charged surfaces after freeze-drying, forming EDLs within the hyphae for ion transport regulation (Figure 1e–f)." (page 4 line 103)

"The ion transport in the mycelial network is primarily associated with nanoconfined channels within the hyphae rather than microscale void spaces between hyphae. The spaces between hyphae observed in Figure 2b are typically on the order of tens of micrometers, which are substantially larger than the Debye length in dilute electrolytes (typically ~10–100 nm). Such large channels do not support electric double layer overlap and therefore cannot sustain nanofluidic transport. This interpretation is further supported by compression tests, where compressed samples with reduced void volume between hyphae exhibit ion conductivities comparable to those of uncompressed samples (Supplementary Figure 11). The nanoporous structure within the hyphae is also expected to carry negative charge similar to the hyphal surface, likely arising from cell membrane disruption and the dissociation of functional groups, thereby allowing cation migration within the hyphal interior. In addition, dye-tracing experiments reveal that the mycelial hyphae form an interconnected network supporting continuous ion transport pathways (Supplementary Figure 12), enabled by hyphal fusion and extracellular matrix components45, 51. Comparisons between live and freeze-dried mycelium further highlight the role of nanoporosity generated during freeze-drying in enabling nanofluidic ion transport (Supplementary Figure 13)." (page 7 line 164)

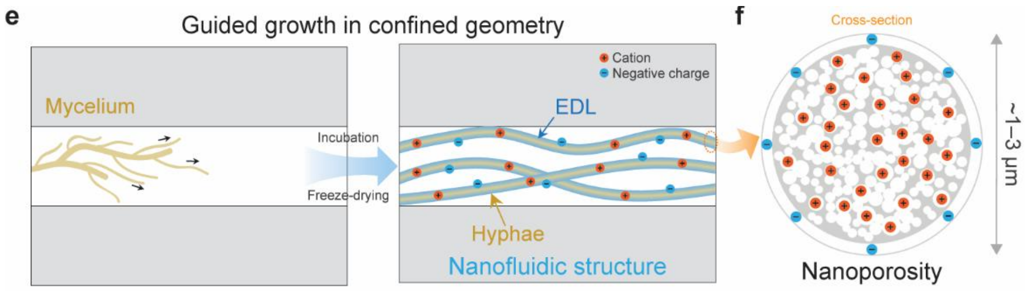

Figure 1. Ubiquitous and scalable mycelium and its guided growth in confined geometries. (e) Schematic of guided mycelium growth in a confined geometry, driven by growth resources such as nutrients and oxygen. The extended self-grown hyphae emerge as nanofluidic structures after freeze-drying, forming electric double layers (EDLs) that regulate cations, thus enabling nanofluidics. (f) Schematic of a cross-sectional hypha, showing its nanoporosity. The mycelial cell wall is inherently negatively charged due to the dissociation of functional groups, with nanopores within the hypha serving as pathways for cations.

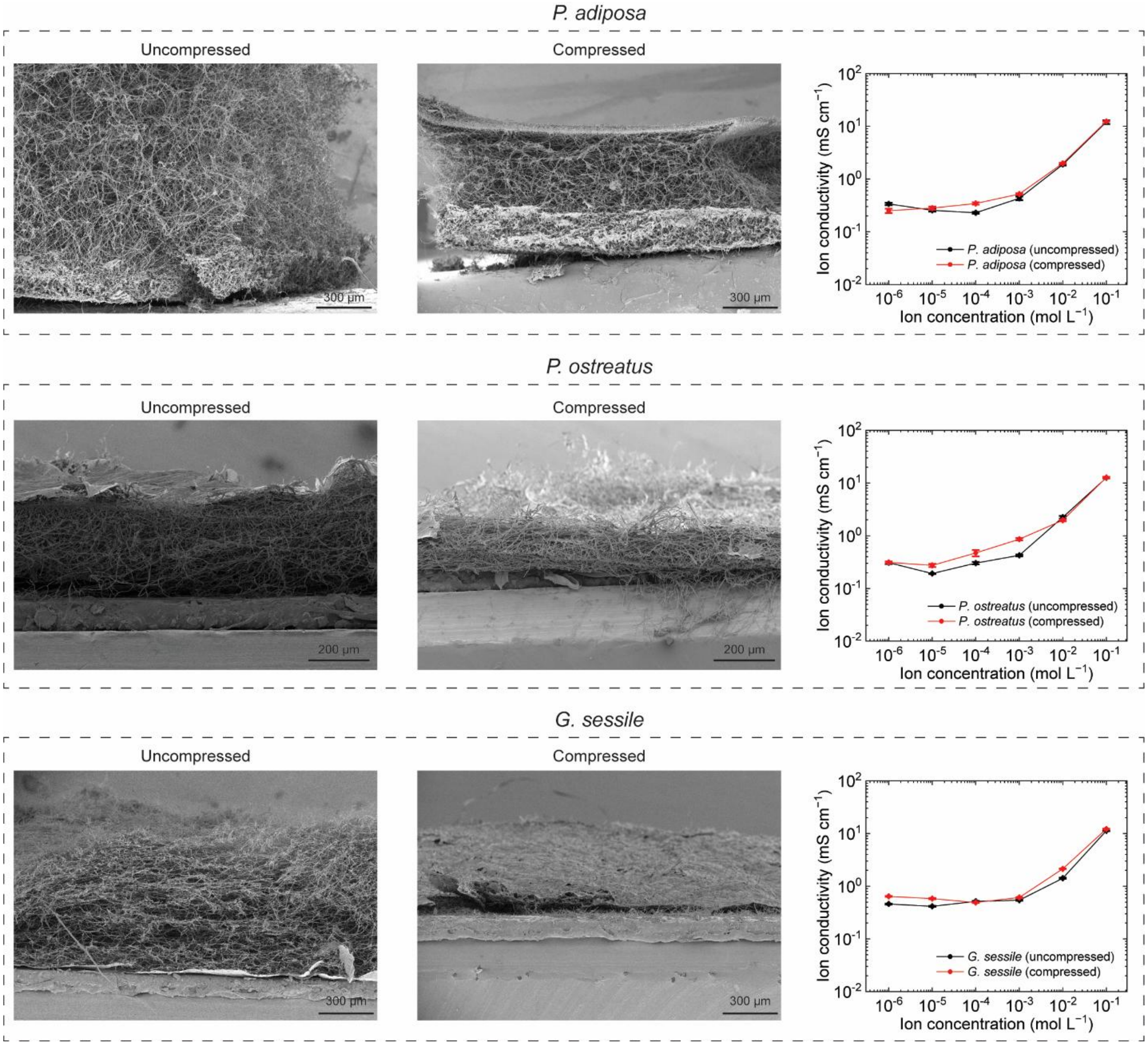

Supplementary Figure 11. SEM images of original (uncompressed) and compressed mycelium samples, together with the corresponding ion conductivity measurements. The compressed samples were subjected to a pressure of 6.9 MPa for 1 min, resulting in reduced void volume between hyphae. The comparable conductivity observed for uncompressed and compressed samples suggests that the ion transport primarily occurs through the nanoconfined hyphal network rather than through microscale void spaces between adjacent hyphae.

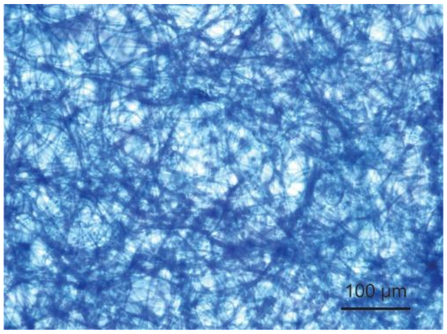

Supplementary Figure 12. Optical microscope image of the *P. adiposa* hyphal network stained with 1×10^{-4} mol L^{-1} methylene blue solution for dye-tracing experiments. The uniform staining indicates that the mycelial hyphae form an interconnected network with continuous transport pathways.

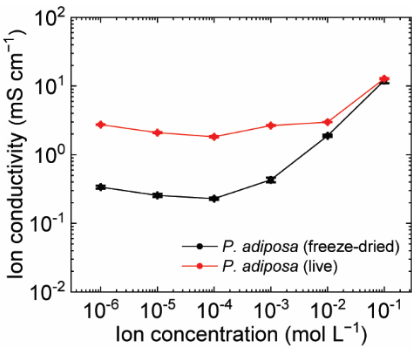

Supplementary Figure 13. Ion conductivity of *P. adiposa* mycelium measured in live and freeze-dried states. Freeze-drying preserves the nanostructured porosity of the hyphal network while removing intracellular fluids, ruling out metabolically driven transport processes and enabling clearer observation of nanofluidic ion transport through the mycelial network.

3. In the section describing mycelium-integrated device performance (line 245), the authors state that the self-growing and self-adaptable nature of mycelium enables filling of confined spaces and opens opportunities for nanofluidic devices. However, the degree of channel filling is not clearly quantified. Since the manuscript positions this system as a self-adaptable material platform for nanofluidics, more detailed characterization would be valuable. For example, how do variations in filling density, incubation time, or growth direction affect ionic conductivity and ICR for the system? In Figure 4, does the direction of mycelium growth influence rectification behavior?

Additionally, were these measurements performed on live or freeze-dried samples? It would also be important to clarify whether ion transport and nanofluidic properties change during different stages of the biological life cycle or after freeze-drying. These experimental details should be clarified.

Response: We thank the reviewer for these valuable comments. We agree that quantifying the degree of channel filling would strengthen the characterization of this material platform. In the revised manuscript, **we estimate the filling extent** based on optical images and correlate it with incubation time, which provides a semi-quantitative indicator of filling density within the microchannels.

We have **expanded discussion on the effect of filling** on the conductivity, and we have also **supplemented rectification measurements** with respect to the growth direction of the mycelium, showing that the rectification behavior is largely independent of growth direction because the mycelial network forms a relatively uniform fibrous structure within the channel.

In addition, we **compared live and freeze-dried mycelium** to clarify the origin of ion transport. Clear nanofluidic ion transport is observed in the freeze-dried state, which rules out metabolically driven transport processes. Because the nanofluidic measurements in this work are performed on freeze-dried mycelium, the transport behavior is governed by the structural nanoconfined channels rather than biological activity. Therefore, variations associated with different stages of the biological life cycle do not affect the nanofluidic properties characterized in this study.

Regarding "*However, the degree of channel filling is not clearly quantified. Since the manuscript positions this system as a self-adaptable material platform for nanofluidics, more detailed characterization would be valuable. For example, how do variations in filling density, incubation time affect ionic conductivity and ICR for the system*"

We thank the reviewer for this comment, and we agree that filling behavior is important for this self-adaptable material platform. The filling density at the cross-section is difficult to characterize as cross-sectional cutting would disturb the hyphal network. This effect is evident in the SEM images (Supplementary Figure 18), where part of the hyphal network was disturbed during sample preparation for SEM imaging.

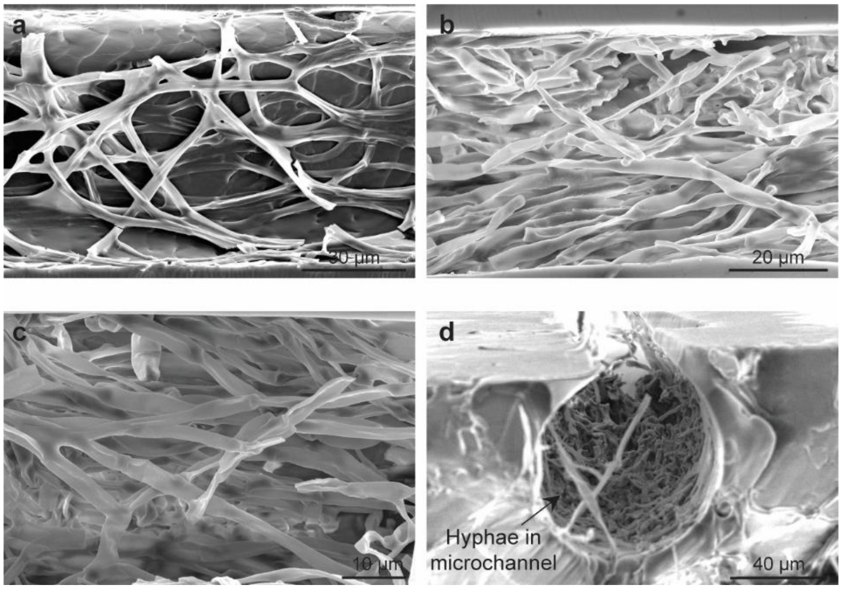

Supplementary Figure 18. SEM images of self-grown *P. adiposa* in the microchannel, forming an interwoven hyphal network that enables functional nanofluidic ion transport. The hyphal network fills the microchannel, as shown by the optical microscope images (Figure 3d). It is noted that the SEM images here do not clearly show the fully filled structure because part of the hyphal network can be mechanically disturbed or removed when the PDMS microchannel is detached from the glass substrate for SEM imaging.

Instead, we analyzed the filling behavior as a function of incubation time using grayscale values from optical microscope images. A fixed region of interest (ROI) within the microchannel was selected, and the grayscale intensity within this region was tracked over time. The results (Supplementary Figure 16) show that it takes ~30 hours for mycelium to fill the cross-sectional area of the microchannel. The final filling fraction is estimated to be on the order of ~5–20% by comparing the apparent density of the porous mycelial network with that of individual mycelial hyphae, as discussed in Supplementary Note 3.

Regarding the effect of filling density on ion conductivity and rectification, in this work we kept sufficiently long incubation times to ensure the formation of a dense mycelial hyphal network within the microchannel, thereby maintaining measurement consistency. At present, precise control of the filling density for a specific mycelium species (e.g., *P. adiposa*) remains challenging, as growth is primarily governed by biological processes under nutrient supply. Achieving

controlled filling densities would require additional strategies, such as introducing porous physical architectures to restrict hyphal growth, which is beyond the scope of this study. Nevertheless, qualitatively, a lower filling density would correspond to fewer hyphae within the microchannel, reducing the number of available ion conduction pathways. This would decrease the measured ion current and thus the apparent conductivity based on the microchannel geometry (diameter and length). This effect has been discussed in the revised manuscript.

We have expanded the discussion as follows:

"It takes ~30 hours for aerial mycelium to fill a cross-sectional plane in the microchannel (Supplementary Figure 16) and several days to traverse the entire microchannel, while submerged mycelium grows ~9 times faster (Supplementary Figure 17), because the nutrients are supplied directly from the surrounding liquid medium rather than being transported through the hyphal network as in aerial mycelium." (page 9 line 223)

"The conductivity may also depend on the filling density of the hyphal network within the microchannel, which varies with mycelium species, incubation time, and nutrient supply. A lower filling density leads to fewer available ion transport pathways and consequently lower effective conductivity of the microchannel. Precise control of the filling density for biologically grown networks remains challenging and requires further investigation." (page 10 line 248)

"The samples were cultivated for a sufficiently long incubation time (>10 days) to ensure a dense hyphal network within the microchannel, thereby maintaining measurement consistency." (page 20 line 484)

"Previous studies4, 5 report bulk densities of mycelium networks on the order of 50–200 kg m^{-3}, while individual hyphae—containing 80–90% water—have densities close to that of water (1000–1100 kg m^{-3}). This suggests that the mycelium network has a filling ratio f around 5–20%." (Supplementary Note 3)

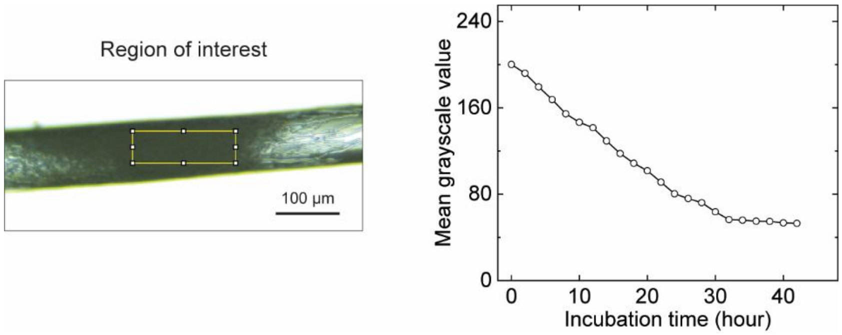

Supplementary Figure 16. Mean grayscale value measured within a fixed region of interest (ROI) in the microchannel to track *P. adiposa* hyphal growth over incubation time, analyzed using ImageJ. As incubation time increases, the denser hyphal network results in lower grayscale intensity. The filling ratio of the hyphal network is estimated to be around 5–20% by comparing the apparent density of the porous mycelial network with that of individual mycelial hyphae, as discussed in Supplementary Note 3. This filling ratio corresponds to an estimated ~50–170 hyphae spanning the microchannel cross-section. Direct experimental determination is challenging because cross-sectional cutting would disrupt the interconnected hyphal network and alter its native structure, thereby affecting accurate characterization.

Regarding "*how do growth direction affect ionic conductivity and ICR for the system? In Figure 4, does the direction of mycelium growth influence rectification behavior?*"

We thank the reviewer for this insightful comment. The growth direction does not affect conductivity measurement, as evidenced by the I–V curves (Supplementary Figure 20), where the current remains the same when the bias direction is reversed. The reason is that mycelium forms a fibrous network composed of numerous interconnected hyphae that serve as ion transport pathways, which reduces structural anisotropy and eliminates directional asymmetry in ion transport along the growth direction.

Regarding the rectification, we supplemented a test in which the ion concentration gradient was applied either along or opposite to the growth direction, and the results show negligible differences in the rectification ratio (Supplementary Figure 28). The reason is again attributed to the fibrous network of numerous interconnected hyphae, which provides multiple ion transport pathways and minimizes directional asymmetry.

We have expanded discussion in the manuscript:

"Moreover, the conductivity does not depend on the growth direction as indicated by the symmetric I–V curves (Supplementary Figure 20), and is also not expected to depend strongly on the channel geometry. This is because the mycelium serves as the primary ion conduction medium and forms a fibrous network composed of numerous interconnected hyphae, which reduces structural anisotropy and minimizes directional asymmetry in ion transport." (page 10 line 252)

"The rectification also does not show a dependence on the growth direction (Supplementary Figure 28), which is attributed to the fibrous network of numerous interconnected hyphae that provide multiple ion transport pathways." (page 15 line 346)

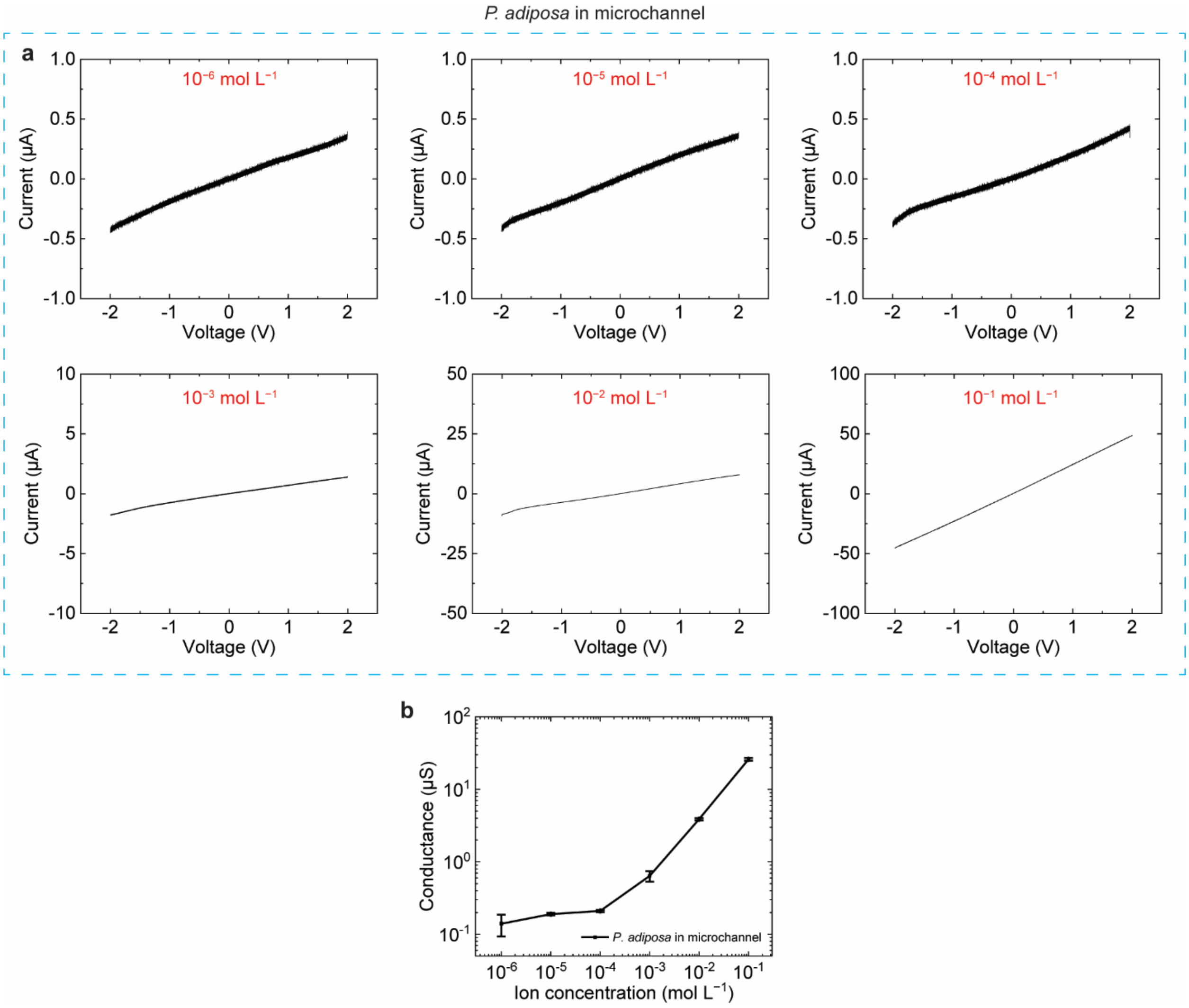

Supplementary Figure 20. I–V characterization of the *P. adiposa* hyphal network in the microchannel, clearly demonstrating nonlinearity due to nanofluidic properties. (a) Representative

I–V curves. (b) Conductance derived from the I–V curves. Full raw data are provided in the source data file. The I–V curves are symmetric under forward and reverse bias, indicating that the measured conductivity is independent of the mycelial growth direction within the microchannel.

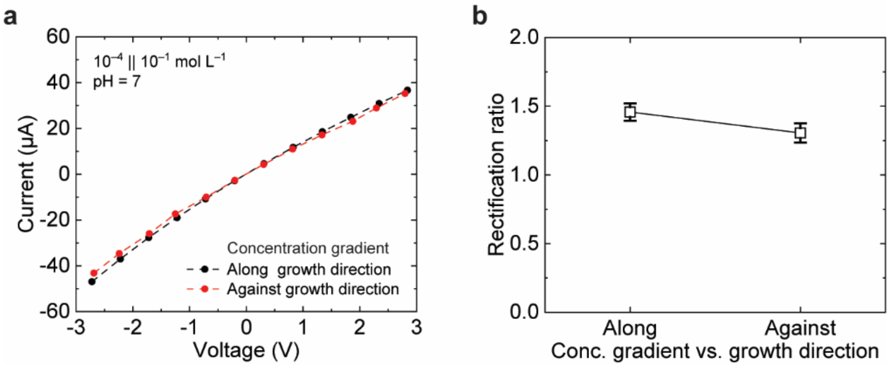

Supplementary Figure 28. Rectification behavior for concentration gradients applied along or opposite to the mycelial growth direction. (a) Current–voltage response. (b) Corresponding rectification ratio, showing negligible difference between the two directions. This behavior is consistent with the fibrous network structure of mycelium, composed of numerous interconnected hyphae that reduce structural anisotropy and directional asymmetry in ion transport.

Regarding "*Additionally, were these measurements performed on live or freeze-dried samples? It would also be important to clarify whether ion transport and nanofluidic properties change during different stages of the biological life cycle or after freeze-drying. These experimental details should be clarified.*"

We thank the reviewer for this question. All nanofluidic measurements reported in this work were performed on freeze-dried mycelium samples. Freeze-drying removes intracellular fluids and metabolic activity while preserving the nanostructured porosity of the hyphal network, thereby allowing the ion transport behavior to be attributed to structural nanoconfined pathways rather than biologically driven processes. The results show that clear nanofluidic ion transport behavior is observed in the freeze-dried state, which rules out metabolically driven transport processes associated with live cells. Because the nanofluidic measurements in this study are conducted on

freeze-dried samples, the ion transport properties are governed primarily by the structural features of the hyphal network rather than by different stages of the biological life cycle.

The corresponding clarification has been added to the revised manuscript:

"Comparisons between live and freeze-dried mycelium further highlight the role of nanoporosity generated during freeze-drying in enabling nanofluidic ion transport (Supplementary Figure 13)." (page 7 line 176)

"To measure ion conductivity, all samples were freeze-dried to preserve the nanostructured porosity of the hyphal network and eliminate metabolic activity associated with living cells." (page 22 line 522)

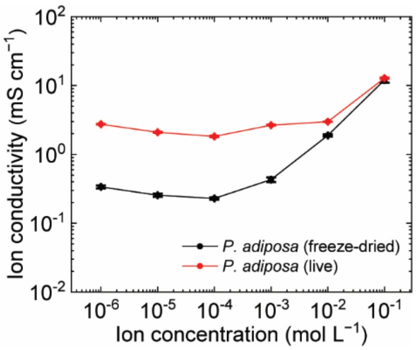

Supplementary Figure 13. Ion conductivity of *P. adiposa* mycelium measured in live and freeze-dried states. Freeze-drying preserves the nanostructured porosity of the hyphal network while removing intracellular fluids, ruling out metabolically driven transport processes and enabling clearer observation of nanofluidic ion transport through the mycelial network.

4. The authors fabricate a relatively large microchannel (~88 µm, defined by the copper wire), which is filled by a network of multiple fibers. Can the authors estimate approximately how many individual hyphae span the channel cross-section? Furthermore, how sensitive is the ionic current or ICR changes to the confined channel geometry itself? If the channel width is reduced, does the ion transport or ICR behavior change significantly? Is there a minimum channel dimension below which mycelium cannot bridge the gap? Could a single-hypha device even be made possible? Addressing these questions would help define the scalability and practical limitations of this approach.

Response: We thank the reviewer for the valuable comments. We have answered them one-by-one below.

Regarding "*The authors fabricate a relatively large microchannel (~88 μm, defined by the copper wire), which is filled by a network of multiple fibers. Can the authors estimate approximately how many individual hyphae span the channel cross-section?*"

We thank the reviewer for this question. From the estimated filling ratio f of 5–20% (Supplementary Note 3), the number of hyphae spanning the microchannel cross-section would be $f \times (D_{channel}/D_{hyphae})^2$, which corresponds to ~50–170 hyphae. This estimate assumes that the hyphae are randomly distributed within the channel cross-section. Direct experimental determination is challenging because cross-sectional cutting would disrupt the interconnected hyphal network and alter its native structure, thereby affecting the characterization result. Therefore, we provide this order-of-magnitude estimate based on geometric considerations and the measured filling fraction. **We have expanded the discussion:** "This filling ratio corresponds to an estimated ~50–170 hyphae spanning the microchannel cross-section. Direct experimental determination is challenging because cross-sectional cutting would disrupt the interconnected hyphal network and alter its native structure, thereby affecting accurate characterization." (Supplementary Figure 16 caption)

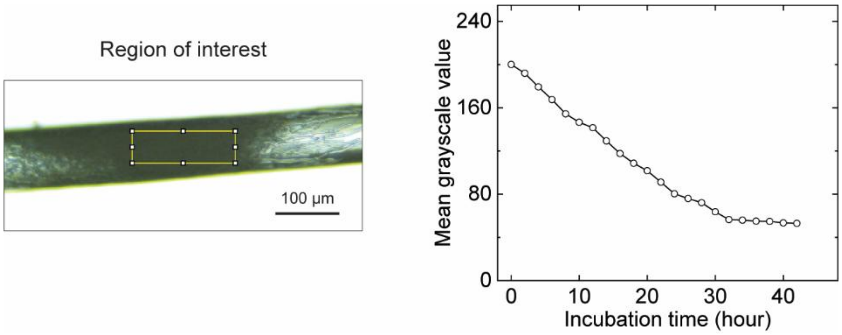

Supplementary Figure 16. Mean grayscale value measured within a fixed region of interest (ROI) in the microchannel to track *P. adiposa* hyphal growth over incubation time, analyzed using ImageJ. As incubation time increases, the denser hyphal network results in lower grayscale intensity. The filling ratio of the hyphal network is estimated to be around 5–20% by comparing the apparent density of the porous mycelial network with that of individual mycelial hyphae, as discussed in Supplementary Note 3. This filling ratio corresponds to an estimated ~50–170 hyphae spanning the microchannel cross-section. Direct experimental determination is challenging because cross-

sectional cutting would disrupt the interconnected hyphal network and alter its native structure, thereby affecting accurate characterization.

Regarding "*Furthermore, how sensitive is the ionic current or ICR changes to the confined channel geometry itself? If the channel width is reduced, does the ion transport or ICR behavior change significantly?*"

We thank the reviewer for this insightful question. The ionic transport in the present device is primarily governed by the nanoconfined pathways within the mycelial hyphae rather than by the microscale geometry of the enclosing channel. The microchannel defined by the copper wire (~88 μm) mainly serves as a macroscale scaffold that hosts the interconnected hyphal network. Once the channel is densely filled with hyphae, the effective ion transport pathways are determined by the nanoporous structures within the hyphae and the interconnected hyphal network, which exhibit characteristic dimensions on the order of tens of nanometers (Supplementary Note 4). For any confined geometry that mycelium can grow into, the channel size must be comparable to or larger than the hyphal diameter (on the order of several micrometers). In this case, the nanofluidic ion transport arising from the nanoporous structures within the hyphae still dominates. Reducing the channel width would mainly reduce the number of parallel ion transport pathways formed by hyphae, which may decrease the overall current magnitude but is not expected to fundamentally change the nanofluidic transport mechanism. Therefore, **the overall ionic conductivity and rectification behavior are expected to be relatively insensitive to moderate variations in the microchannel diameter, as long as a sufficiently dense hyphal network is established within the channel.** However, when the channel size is significantly reduced and the mycelial network contains only a few hyphae, the transport behavior may change, enabling future studies of single-hypha or few-hypha nanofluidic devices.

The similar conductivity values obtained from bulk mycelium (Figure 2e) and the mycelium-integrated microchannel device (Figure 3g) further support this interpretation. In addition, we supplemented a rectification comparison between bulk mycelium (Supplementary Figure 27) and the microchannel device (Figure 4e–f), which also shows comparable performance, indicating that the influence of the microchannel geometry on ion transport is limited.

We have expanded the discussion in the revised manuscript:

“This interpretation is further supported by the observation that the device performance is consistent with that of bulk mycelium materials (Figure 2e).” (page 10 line 245)

“Moreover, the conductivity does not depend on the growth direction as indicated by the symmetric I–V curves (Supplementary Figure 20), and is also not expected to depend strongly on the channel geometry. This is because the mycelium serves as the primary ion conduction medium and forms a fibrous network composed of numerous interconnected hyphae, which reduces structural anisotropy and minimizes directional asymmetry in ion transport. When the channel size is significantly reduced and the mycelial network contains only a few hyphae, however, the transport behavior may change, enabling future studies of single-hypha or few-hypha nanofluidic devices.” (page 10 line 252)

“The forward and reverse conductances exhibit a modest rectification ratio of 1.46±0.06 (Figure 4f and Supplementary Figure 26), which is consistent with that observed in bulk mycelium (Supplementary Figure 27).” (page 14 line 337)

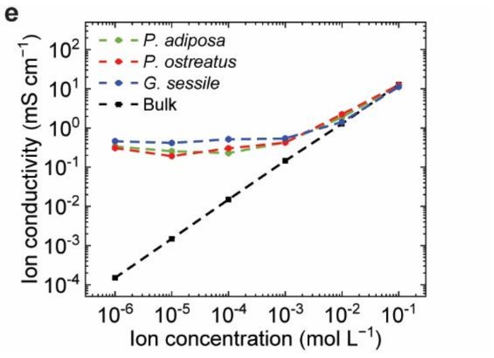

Figure 2. Universal micro-nanoporous structure and nanofluidic properties of multispecies mycelium. (e) Ion conductivity measurements (Supplementary Figure 14) of the three mycelium membranes show substantially higher conductivity than bulk values at KCl concentrations below 1×10^{-3} mol L^{-1}, illustrating their universal ion regulation capability.

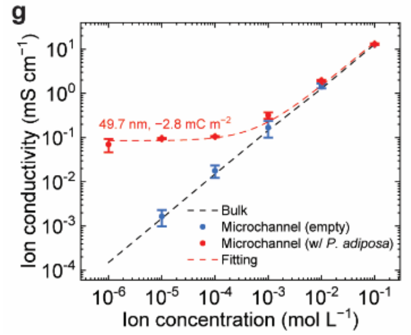

Figure 3. Self-grown mycelium-enabled nanofluidics. (g) Ion conductivity of the microchannel when empty versus with *P. adiposa* hyphae, showing that the integration of mycelium introduces nanofluidic ion regulation performance.

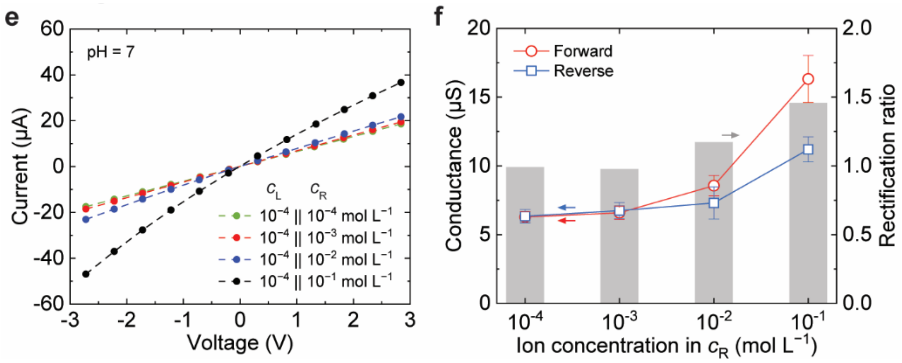

Figure 4. Performance and visualization of self-grown nanofluidic devices. (e) current–voltage response, and (f) the resultant rectification ratio induced by the bath concentration gradient.

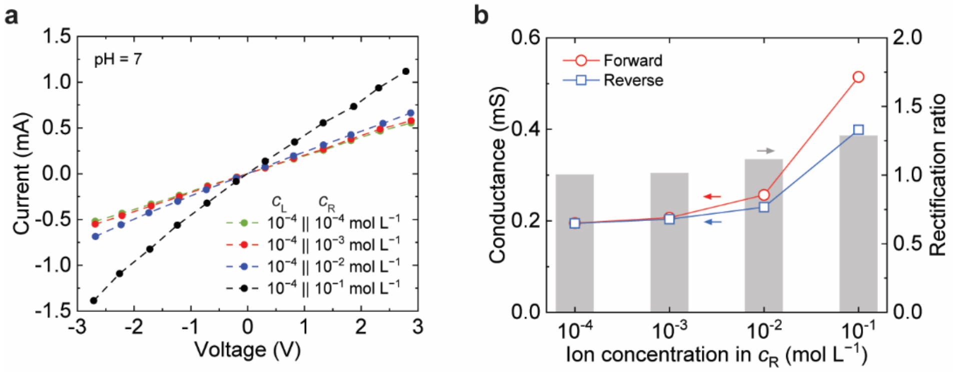

Supplementary Figure 27. Current–voltage measurements of bulk *P. adiposa* mycelium under asymmetric bath concentrations. (a) Current–voltage response. (b) Corresponding rectification

ratio, reaching up to 1.3. This behavior is consistent with the device measurements shown in Figure 4e–f.

Regarding "*Is there a minimum channel dimension below which mycelium cannot bridge the gap? Could a single-hypha device even be made possible? Addressing these questions would help define the scalability and practical limitations of this approach.*"

We thank the reviewer for this insightful comment, which raises an important perspective on the scalability and potential of the proposed approach. We agree that the development of single-hypha devices would be of great importance. We have fabricated a device with a cross-sectional aera of ~5 μm × 5 μm, and mycelial hyphae are still observed to pass through the channel (Supplementary Figure 37). However, such a small cross-section results in ionic currents on the order of nA in conductivity measurements, which goes beyond the lower detection limit of our measurement setup, making reliable characterization challenging.

Based on these observations, our current understanding is that hyphae can grow through confined channels as long as the channel dimension is comparable to or larger than the hyphal diameter, although a systematic study of the minimum channel dimension is beyond the scope of the present work. Therefore, with carefully designed channel geometries and controlled incubation conditions, a single-hypha device could be realized in principle. Such a single-fiber nanofluidic device could enable precise studies of ion transport within individual hyphae and provide a platform for highly sensitive nanofluidic sensing applications.

To further address the reviewer's comment on the scalability and future possibilities of this approach, **we have expanded the discussion and outlined the following vision in the revised manuscript:**

"Looking forward, the growth-driven nature of mycelium opens opportunities for programmable nanofluidic architectures guided by channel geometry, nutrient gradients, or environmental stimuli. By tailoring microchannel dimensions comparable to the hyphal diameter, it may become possible to guide the growth of individual hyphae through confined geometries (Supplementary Figure 37), enabling single-hypha nanofluidic devices for studying ion transport within individual biological conduits. Furthermore, such ion transport networks could be designed using microfluidic platforms

to guide mycelial growth, enabling pathways with tunable connectivity and hierarchical structure. These capabilities suggest a new paradigm in which nanofluidic systems are not only fabricated but biologically grown and spatially organized, allowing adaptive ion transport pathways to emerge within complex microfluidic or biological environments." (page 19 line 436)

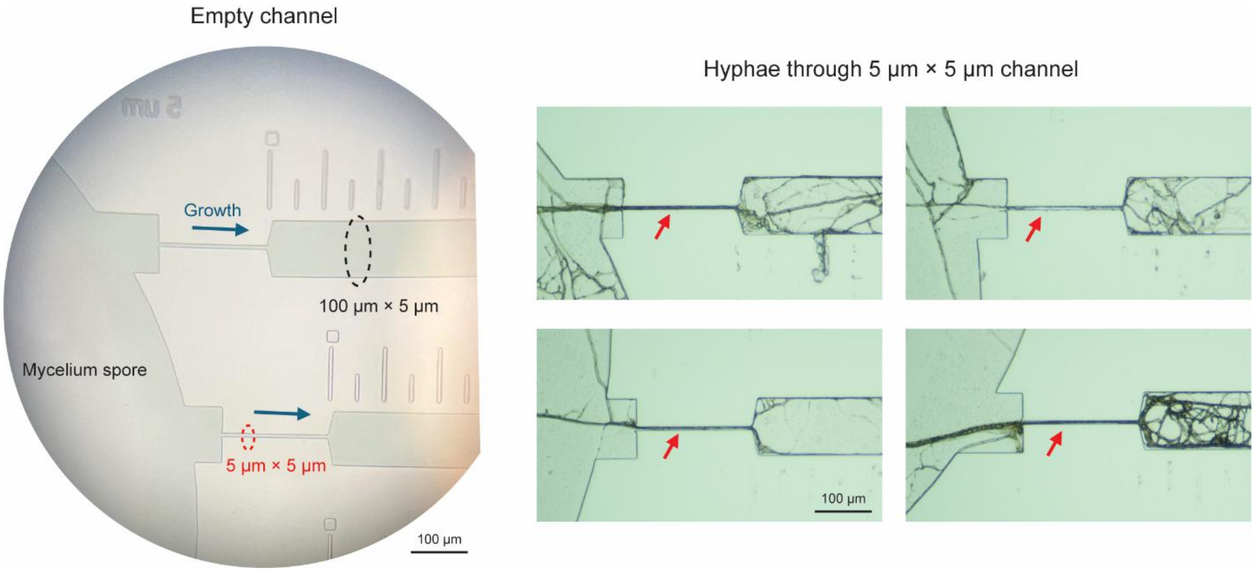

Supplementary Figure 37. Demonstration of *Lactarius* mycelium hyphae growing through a 5 μm × 5 μm microchannel. Mycelium spores were inoculated into the left chamber of the microfluidic device, and the resulting hyphae extended through the 5 μm × 5 μm channel and subsequently proliferated in the microchannel of 100 μm width. This result demonstrates that mycelium can successfully grow through single-digit micrometer-scale channels, supporting the feasibility of single-hypha nanofluidic devices.

References in Response Letter

1. Si L*, et al.* A superstable, flexible, and scalable nanofluidic ion regulation composite membrane. *Science Bulletin* **68**, 2344-2353 (2023).
2. Li T*, et al.* A nanofluidic ion regulation membrane with aligned cellulose nanofibers. *Science Advances* **5**, (2019).
3. Qin S*, et al.* High and Stable Ionic Conductivity in 2D Nanofluidic Ion Channels between Boron Nitride Layers. *Journal of the American Chemical Society* **139**, 6314-6320 (2017).
4. Glass NL, Jacobson DJ, Shiu PKT. The Genetics of Hyphal Fusion and Vegetative Incompatibility in Filamentous Ascomycete Fungi. *Annual Review of Genetics* **34**, 165-186 (2000).
5. Riquelme M*, et al.* Fungal Morphogenesis, from the Polarized Growth of Hyphae to Complex Reproduction and Infection Structures. *Microbiology and Molecular Biology Reviews* **82**, (2018).
6. Ai Y, Zhang M, Joo SW, Cheney MA, Qian S. Effects of Electroosmotic Flow on Ionic Current Rectification in Conical Nanopores. *The Journal of Physical Chemistry C* **114**, 3883-3890 (2010).
7. Vlassiouk I, Kozel TR, Siwy ZS. Biosensing with Nanofluidic Diodes. *Journal of the American Chemical Society* **131**, 8211-8220 (2009).
8. Kovarik ML, Zhou K, Jacobson SC. Effect of Conical Nanopore Diameter on Ion Current Rectification. *The Journal of Physical Chemistry B* **113**, 15960-15966 (2009).
9. Money NP. Measurement of pore size in the hyphal cell wall ofAchlya bisexualis. *Experimental Mycology* **14**, 234-242 (1990).
